# Tris(tetramethylguanidinyl)phosphine: The Simplest Non‐ionic Phosphorus Superbase and Strongly Donating Phosphine Ligand

**DOI:** 10.1002/chem.202104021

**Published:** 2021-12-09

**Authors:** Florenz Buß, Maike B. Röthel, Janina A. Werra, Philipp Rotering, Lukas F. B. Wilm, Constantin G. Daniliuc, Pawel Löwe, Fabian Dielmann

**Affiliations:** ^1^ Institut für Anorganische und Analytische Chemie Westfälische Wilhelms-Universität Münster Corrensstraße 28–30 48149 Münster Germany; ^2^ Institute of General, Inorganic and Theoretical Chemistry University of Innsbruck, Center for Chemistry and Biomedicine Innrain 80–82 A-6020 Innsbruck Austria; ^3^ Organisch-Chemisches Institut Westfälische Wilhelms-Universität Münster Corrensstraße 40 48149 Münster Germany

**Keywords:** carbon dioxide, phosphine ligands, phosphorus, strong donors, superbases

## Abstract

We report the synthesis and properties of the much sought‐after tris(1,1,3,3‐tetramethylguanidinyl) phosphine P(tmg)_3_, a crystalline, superbasic phosphine accessible through a short and scalable procedure from the cheap and commercially available bulk chemicals 1,1,3,3‐tetramethylguanidine, tris(dimethylamino)‐phosphine and phosphorus trichloride. The new phosphine exhibits exceptional electron donor properties and readily forms transition metal complexes with gold(I), palladium(II) and rhodium(I) precursors. The formation of zwitterionic Lewis base adducts with carbon dioxide and sulfur dioxide was explored. In addition, the complete series of phosphine chalcogenides was prepared from the reaction of P(tmg)_3_ with N_2_O and the elemental chalcogens.

## Graphical abstract

**The crystalline phosphorus** superbase tris(1,1,3,3‐tetramethylguanidinyl)phosphine is accessible through an easy and scalable procedure starting from cheap and commercially available bulk chemicals. The properties of the strongly electron‐donating phosphine in coordination chemistry and the reactivity with small molecules (CO_2_, SO_2_, N_2_O and elemental chalcogens) were studied.

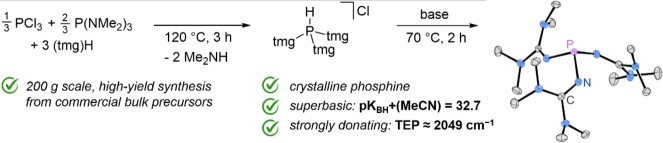

## Introduction

Trivalent phosphorus(III) compounds are well established in many fields of academic research and industrial applications. The possibility of rational tuning of their steric and electronic properties had a decisive influence on the ever‐growing use of P(III) donors in the last decades.^[1]^ Recently, our group and others expanded the spectrum of available stereoelectronic properties by synthesizing and characterizing a family of highly electron‐rich phosphines bearing imidazolin‐2‐ylidenamino,^[2]^ pyridinylidenamino,^[3]^ phosphazenyl[^4^, ^5^] and phosphoniumylidyl groups.^[4]^ The resulting very basic phosphines have shown the capacity to react with particularly inert small molecules, including CO_2_, SO_2_, N_2_O and SF_6_,[^6^, ^7^, ^8^] and they have been successfully applied as ligands in coordination chemistry and catalysis.[^2^, ^4^, ^9^, ^10^, ^11^, ^12^, ^13^, ^14^, ^15^, ^16^, ^17^, ^18^, ^19^] Prospective widespread application of superbasic phosphines as ligands, but more importantly as nucleophiles in stoichiometric reactions, will require synthetic protocols that enable their easy and scalable preparation from cheap starting materials. While imidazolin‐2‐ylidenaminophosphines (IAP) have the advantage that their steric and electronic properties can be varied over a wide range by modification of the imidazole backbone, their synthesis often involves several reaction steps.[^2^, ^6^, ^13^]

**Figure 1:**
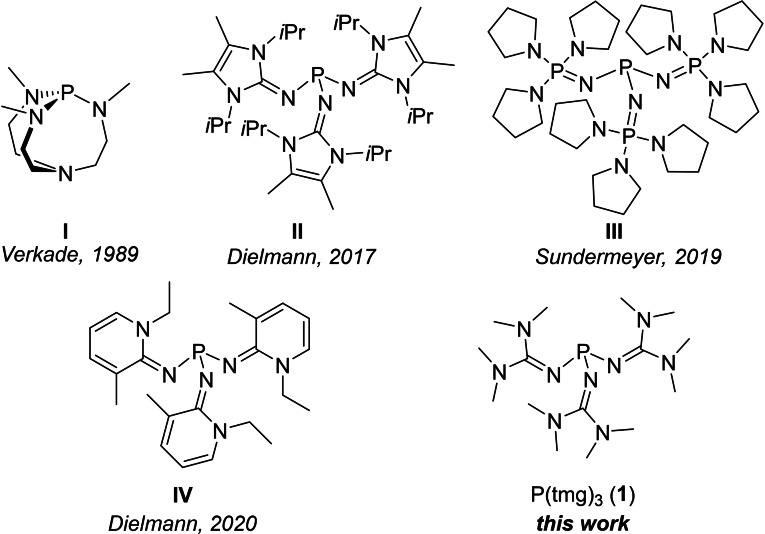
Selected superbasic phosphines.

For example, phosphine **II** (Figure 1), one of the strongest nonionic superbases with a p*K*_BH_^+^ value in THF of 31.0, can be synthesized in 5 steps with an overall yield of 46 %.^[20]^ The currently strongest known isolable nonionic phosphorus superbase **III** [p*K*_BH_^+^(THF)=37.2)] is accessible in 4 steps in 67 % yield. The pyridine‐2‐ylidenamino phosphine **IV** is more readily accessible in 3 steps starting from *ortho*‐aminopyridine with an overall yield of 64 %.^[3]^ From a rational perspective, the simplest phosphorus superbase would be P(tmg)_3_ (**1**) which carries three acyclic 1,1,3,3‐tertramethylguanidyl (tmg) substituents at the phosphorus atom. According to calculations by Leito and co‐workers, the gas‐phase basicity of P(tmg)_3_ (267.1 kcal/mol) is expected to exceed the phosphatrane superbase **I** of Verkade et al.[^21^, ^22^, ^23^] by more than 12 kcal/mol.^[24]^ Schmutzler and co‐workers attempted to synthesize P(tmg)_3_ via several reaction pathways, noting that the phosphine should possess extraordinary basicity.[^25^, ^26^, ^27^, ^28^] Although they succeeded in isolating the P‐protonated form, the free phosphine P(tmg)_3_ could not be liberated. In further studies, the same group prepared a series of phosphines with one and two tmg substituents and investigated their ligand properties.[^25^, ^26^] They observed that the tricarbonylnickel complexes of P(tmg)Ph_2_ and P(tmg)_2_Me show A_1_ carbonyl stretching frequencies at 2080 cm^−1^ and 2060 cm^−1^, respectively. According to the substituent additivity rule proposed by Tolman^[29]^ a substituent parameter for tmg of 15.3 and 0.7 can be derived from these vibrations, respectively. However, these values are clearly inconsistent and rather classify the tmg substituent as electron‐withdrawing than electron‐donating. This stands in marked contrast to the imidazolin‐2‐ylidenamino and pyridinylidenamino substituents, which both have negative substituent parameters and obey the additivity rule.[^2^, ^6^, ^11^, ^13^, ^30^] To shed light on these inconsistencies, we seized on Schmutzler's work and report herein on the synthesis and properties of the hitherto unknown phosphine P(tmg)_3_ (**1**).

## Results and Discussion

**Figure 2:**
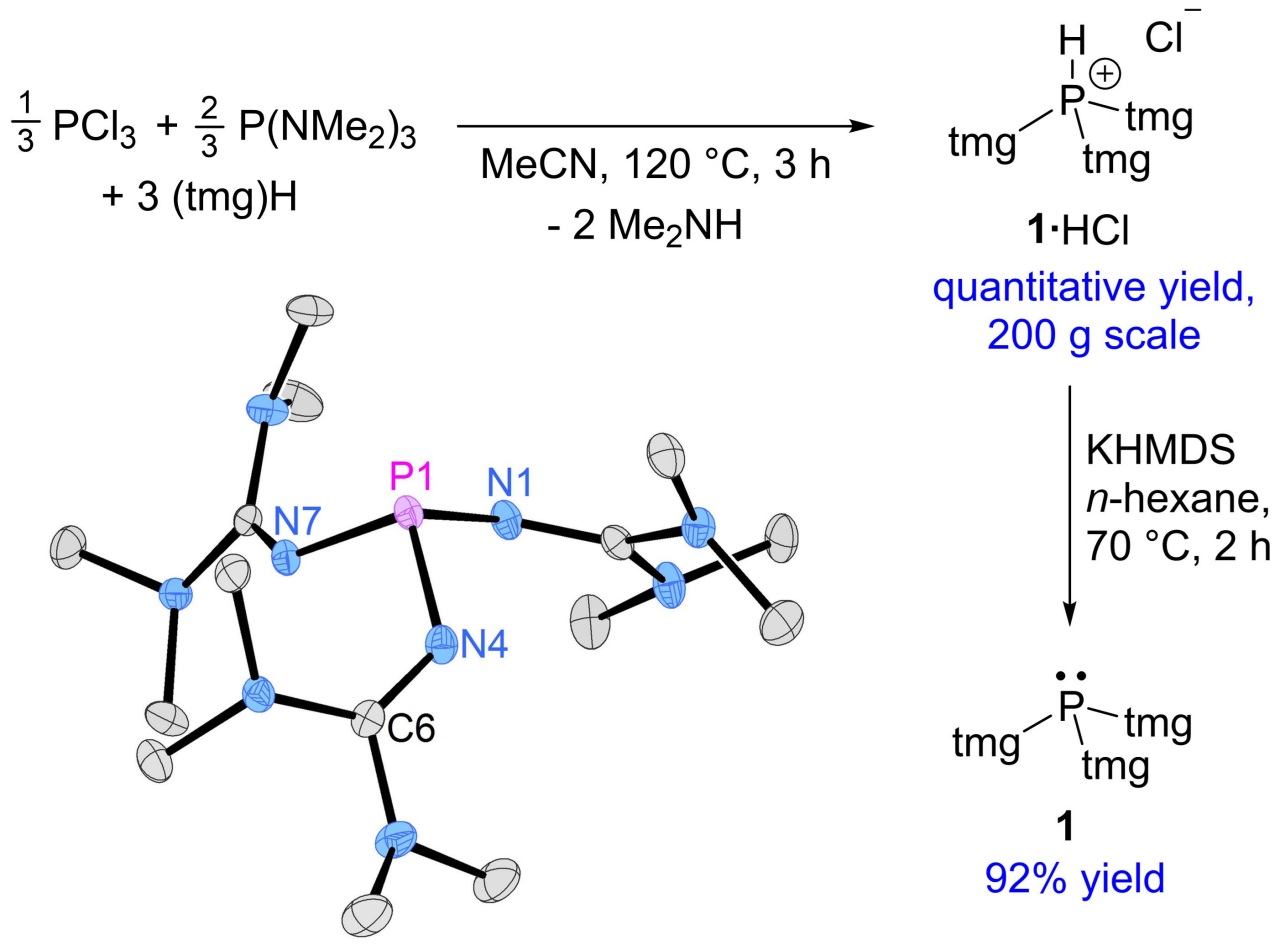
Synthesis of phosphonium salt **1**⋅HCl and phosphine **1**. Molecular structure of **1**. Hydrogen atoms are omitted for clarity; thermal ellipsoids are set at 50 % probability. Selected bond lengths [Å] and angles [°]: P1−N1 1.7076(8), P1−N7 1.7036(8), P1−N4 1.7073(9), N4−C6 1.2805(13), N7−P1−N1 98.31(4), N7−P1−N4 98.76(4), N4−P1−N1 99.15(4).

We have previously shown that the exocyclic imine N atoms of type **II** and **IV** phosphines form stable coordination compounds with alkali metal salts, especially when lithium salts are involved and the imine N atoms are sterically accessible.[^6^, ^13^] Since this property can make the isolation of superbases prohibitively difficult, we decided to avoid lithium bases in the synthesis of tris(tetramethylguanidinyl)phosphine (**1**), and adopted the synthetic procedure used for **I** and **III** by employing PCl(NMe_2_)_2_ as phosphorus precursor.[^5^, ^31^, ^32^] The electrophile PCl(NMe_2_)_2_ has a built‐in auxiliary base and is readily accessible by mixing PCl_3_ and P(NMe_2_)_3_ in 1 : 2 ratio. Thus, heating a mixture of PCl_3_, P(NMe_2_)_3_ and excess (tmg)H in acetonitrile at 120 °C for 3 h afforded the protonated phosphine P(tmg)_3_⋅HCl (**1**⋅HCl) as a yellowish solid after removing the volatiles by distillation in quantitative yield (Figure 2). The reaction can be easily performed on a large scale and is highly atom economical, assuming that the gaseous dimethylamine eliminated during the reaction is used for the regeneration of P(NMe_2_)_3_, and that the distillate containing (tmg)H and acetonitrile is re‐used for the next batch. Notably, the phosphonium salt **1**⋅HCl can be prepared without acetonitrile with the same purity, as shown by ^1^H and ^31^P NMR spectroscopy and elemental analysis, but it was obtained as a sticky substance that is difficult to handle.

The deprotonation of **1**⋅HCl was optimized by varying the reaction conditions including the base and solvent. Challenging aspects in this reaction are the low solubility of **1**⋅HCl in nonpolar solvents, the high reactivity of the free phosphine **1** and the fact that **1** tends to form stable coordination compounds with the alkali metal salts that are generated during the reaction. Best results were thus obtained by using potassium bis(trimethylsilyl)amide (KHMDS) as the base and *n*‐hexane as the solvent. Phosphine **1** was isolated as a white, crystalline solid in 92 % yield. It is soluble in *n*‐hexane, toluene, Et_2_O and THF, but decomposes in chloroform, dichloromethane and acetonitrile due to the high basicity of the phosphorus atom. Phosphine **1** is sensitive towards hydrolysis and oxidation with molecular oxygen,^[33]^ but is stable for months in the absence of air and moisture. The corresponding hydrochloride **1**⋅HCl is highly hygroscopic and slowly hydrolyses under formation of the guanidinium phosphonate in the presence of water. In the ^31^P NMR spectrum of the free phosphine, a sharp singlet appears at δ=83.5 ppm. When **1** is contaminated with KCl, the ^31^P resonance is broadened and shifted to higher frequency. The solid structure of **1** was established by a single‐crystal X‐ray diffraction (XRD) study (Figure 2),^[34]^ which confirms the successful isolation of the salt‐free phosphine. The average P−N bond length of **1** (1.706 Å) is in the range of that of **IV** (1.701 Å) and longer than that of **II** (1.689 Å).[^3^, ^13^] The same trend is observed for the pyramidalization of the phosphorus atom gauged by the sum of NPN angles (**1**: 296.2°, **II**: 294.5°, **IV**: 307.1°).

To evaluate the electron‐donating ability of the new phosphine, we determined the A_1_ CO stretching vibration of complex [(**1**)Ni(CO)_3_] in dichloromethane, revealing a Tolman electronic parameter (TEP)^[35]^ of 2049.1 cm^−1^. Previous work showed that the interaction of the basic imine‐N atoms of type **II** and **IV** phosphines with electrophiles can significantly affect the donor properties of the phosphine.[^3^, ^4b^, ^30^, ^36^] We therefore recorded the IR spectra of [(**1**)Ni(CO)_3_] and [(PPh_3_)Ni(CO)_3_] in different solvents to elucidate the influence of solvent interactions on the donor strength of **1** (Table 1). The A_1_ CO vibration of complex [(PPh_3_)Ni(CO)_3_] is little affected by the solvent and is shifted to lower values by 2.4 cm^−1^ for the solid sample, confirming that the interaction of the carbonyl ligands with the solvent has a negligible effect on the TEP value. By contrast, the A_1_ CO stretching vibration of complex [(**1**)Ni(CO)_3_] is strongly solvent dependent and extends over a range of 17.6 cm^−1^. The highest wave number was obtained in methanol that can form hydrogen bonding interactions with the nitrogen donor atoms of **1**, thereby reducing the overall donor ability of the phosphine. The TEP value of **1** in dichloromethane (2049.1 cm^−1^) is similar to that of the N‐heterocyclic carbene IPent (3‐Bis(2,6‐bis(1‐ethylpropyl)phenyl)imidazol‐2‐ylidene) prepared by Nolan and co‐workers (2049.3 cm^−1^)^[37]^ and higher than that of **VI** (2040.7 cm^−1^),^[3]^ **II** (2029.7 cm^−1^)^[20]^ and **III** (2017.3 cm^−1^, determined in neat substance).^[5]^

**Table 1:** TEP values of PPh_3_ and phosphine **1** neat, and in selected solvents.

Entry	Solvent	TEP in cm^−1^ [Ni(CO)_3_P(tmg)_3_]	TEP in cm^−1^ [Ni(CO)_3_PPh_3_]
1	Neat	2036.5	2066.1
2	Toluene	2041.1	2068.8
4	Tetrahydrofuran	2048.6	2068.0
3	Dichloromethane	2049.1	2068.7
5	Methanol	2054.1	2068.3

The TEP values were calculated for phosphines **1**, P(tmg)Ph_2_, P(tmg)_2_Me and P(NIMe)_3_ (NIMe=1,3‐dimethylimidazolin‐2‐ylidenamino) using the method reported by Gusev to shed light on the abovementioned inconsistencies (Table 2).^[38]^ The calculated TEP value of **1** (2044.5 cm^−1^) agrees with the experimental values of **1** obtained in THF and toluene and ranks **1** in the range of IAPs (TEP of P(NIMe)_3_: 2041.9 cm^−1^). Moreover, the single substituent parameter of the tmg group derived from the calculated TEP values are similar (**1**: −3.9, P(tmg)Ph_2_: −2.8, P(tmg)_2_Me: −5.2). Such deviations from additivity rule are in an expected range.^[39]^ The calculated proton affinities (PA) and gas‐phase basicities (GB) correlate with the TEP values and show the expected trend of increasing phosphine basicity with the number of tmg substituents (Table 2). Collectively, our experimental and computational results show that phosphines with tmg substituents and with the cyclic counterparts (IAPs) have similar electronic properties. However, the nitrogen atoms in tmg‐substituted phosphines are more accessible for interactions with electrophiles. Presumably, this property led to the high TEP values obtained experimentally for P(tmg)Ph_2_ and P(tmg)_2_Me.^[17]^

**Table 2:** Calculated proton affinities (PA) and gas‐phase basicities (GB) and TEP values of **1**, P(tmg)Ph_2_, P(tmg)_2_Me and P(NIMe)_3_ (NIMe=1,3‐dimethylimidazolin‐2‐ylidenamino).

Entry	Phosphine	PA [kcal/mol]	GB [kcal/mol]	TEP in cm^−1^ [calculated]
1	P(tmg)_3_ (**1**)	284.4	275.6	2044.5
2	P(tmg)_2_Me	273.1	264.7	2048.4
3	P(tmg)Ph_2_	254.0	246.6	2061.9
4	P(NIMe)_3_	283.9	276.8	2041.9

We next decided to determine the basicity of phosphine **1** experimentally using the procedure from Morris et al. based on ^31^P NMR analysis of a 1 : 1 mixture of two phosphorus species with similar basicity.^[40]^ The phosphonium salt [(pyrr)_3_PCH_2_Ph](OTf) [p*K*_BH_^+^(THF)=24.3; p*K*_BH_^+^(MeCN)=32.5] was selected as reference.^[41]^ However, apart from the expected species **1**, **1**⋅HOTf, [(pyrr)_3_PCH_2_Ph](OTf) and (pyrr)_3_PCHPh, the phosphinophosphonium salt [P_2_(tmg)_5_]Cl and (tmg)H were detected in the reaction mixture which indicate a second equilibrium. [P_2_(tmg)_5_]Cl was identified by its characteristic doublets at 92.4 and 1.0 ppm (^1^ *J*_PP_=226.2 Hz) in the ^31^P NMR spectrum. The equilibrium **1**+**1**⋅HCl ⇋ P_2_(tmg)_5_Cl+(tmg)H was confirmed by NMR analysis of a 1 : 1 mixture of **1** and **1**⋅HCl in an independent experiment (Figure S56). Moreover, the addition of KHMDS to this mixture led to the formation of phosphine **1**. Taking this second equilibrium into account, the pK_BH_^+^ value of **1** in acetonitrile is determined p*K*_BH_^+^(MeCN)=32.7.

**Scheme 1:**
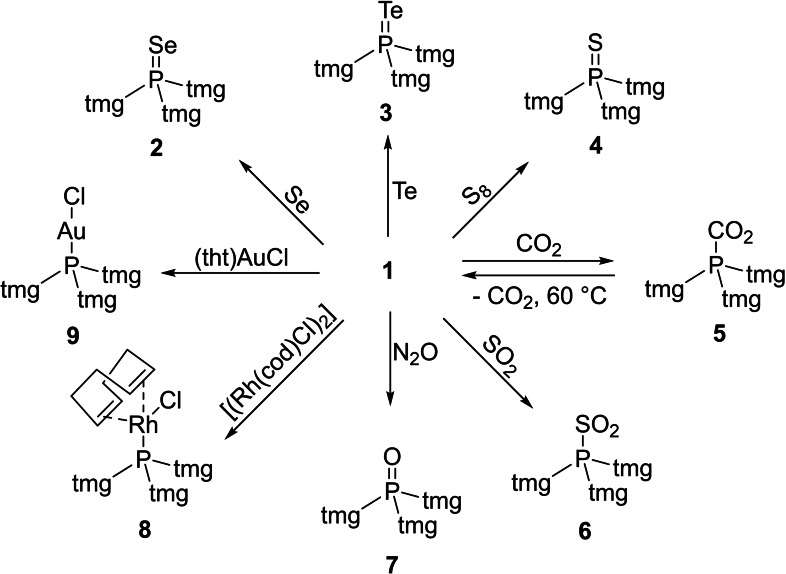
Reaction of phosphine **1** with elemental chalcogens (S, Se and Te), CO_2_, SO_2_, N_2_O as well as with common metal precursors. For reagents and conditions see the Supporting Information.

An alternative method to indirectly assess the phosphine basicity uses the ^1^ *J*_PSe_ coupling constant of the corresponding phosphine selenides showing the trend of decreasing ^1^ *J*_PSe_ with increasing basicity of the phosphine.^[42]^ The phosphine selenide **2** was prepared from the reaction of P(tmg)_3_ with elemental selenium (Scheme 1). In agreement with the gas‐phase basicities, the ^1^ *J*_PSe_ coupling constant of **2** (703 Hz) is smaller than that of Verkade's base **I** (754 Hz) and larger than that of phosphazenylphosphine **III** (628 Hz).[^4^, ^5^] According to the postulated correlation between ^1^ *J*_PSe_ and pK_b_ values for organophosphines,^[42]^ the basicity of **1** is ranked in the range of alkylphosphines and of **I** in the range of aryl phosphines, which indicates that this correlation is not applicable for phosphines with nitrogen substituents.

**Figure 3:**
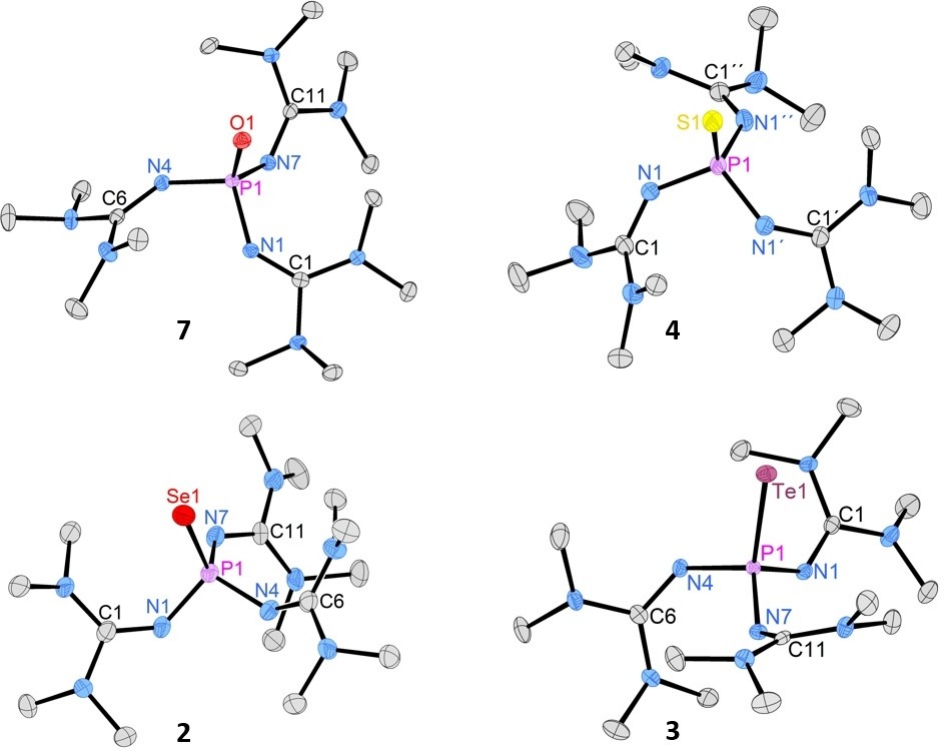
Molecular structure of phosphine chalcogens **2**, **3**,**4** and **7**. Hydrogen atoms are omitted for clarity; thermal ellipsoids are set at 50 % probability. Selected bond length [Å] and angles [°]: **7**: P1−O1 1.4915(9), P1−N1 1.6475(11), P1−N4 : 1.6432(11), P1−N7: 1.6487(11), N4−C6: 1.2842(17), N1−C1: 1.2897(17), N7−C11: 1.2954(17); **4**: P1−S1: 1.994(3), P1−N1: 1.644(4), N1−C1: 1.291(6); **2**: P1−Se1: 2.1527(9), P1−N1: 1.658(3), P1−N4: 1.638(3), P1−N7: 1.632(3), N1−C1: 1.285(4), N4−C6: 1.299(4), N7−C11: 1.209(4); **3**: P1−Te1: 2.4197(6), P1−N1: 1.6475(19), P1−N4: 1.6463(19), P1−N7: 1.6320(19), N1−C1: 1.309(3), N4−C6: 1.304(3), N7−C11: 1.306(3).

To study structural features of the new phosphine, we prepared the complete series of phosphine chalcogenides by reacting **1** with N_2_O and with elemental sulfur and tellurium (Scheme 1). The phosphine chalcogenides **3**, **4** and **7** were obtained as crystalline solids in quantitative yield. Phosphine telluride **3** is formed already at ambient temperature, which corroborates the exceptional basicity of **1** because the oxidation of phosphines with elemental tellurium usually requires elevated temperatures and becomes more difficult with decreasing phosphine basicity.^[43]^ Moreover, **3** is a crystalline solid with a melting point of 123 °C and exhibits enhanced thermal, light and air stability compared with aryl, alkyl or amino phosphine tellurides.^[44]^ In the ^125^Te NMR spectrum of **3** appears a doublet at δ=−79.9 ppm with a ^1^ *J*(^31^P,^125^Te) coupling constant of 1699 Hz. This coupling constant is smaller than those of (*n*Bu)_3_PTe (1732 Hz) and (Me_2_N)_3_PTe (2095 Hz).^[45]^ Phosphine tellurides usually exhibit substantial negative ^125^Te NMR shifts of −492 to −837 ppm,^[41]^ while the ^125^Te resonance of **3** (−79.9 ppm) appears in the range of anionic tellurium compounds such as diphenylselenotellurophosphinate Ph_2_PSeTe^−^ (−73.4 ppm),^[46]^ which agrees with the exceptional electron‐donating ability of phosphine **1**. XRD studies of **2**–**4** and **7** revealed that the phosphine chalcogenides are structurally very similar with regard to the PN_3_ moiety (Figure 3, Table 3). The P−N bond lengths are identical, within experimental error. Most notably, the sum of PNP angles increases in the order oxide<sulfide<selenide<telluride towards the value of 328.5° for an ideal tetrahedral coordination environment at phosphorus.

**Table 3:** Selected ^31^P NMR spectroscopic and XRD structural data of compounds **1**–**10**.

compound	δ [ppm]	^1^ *J*_PX_ [Hz]	av. PN bond length [Å]	Sum of N−P−N angles [°]
(tmg)_3_P (**1**)	83.5^[a]^		1.706(1)	296.2(1)
(tmg)_3_PSe (**2**)	13.2^[b]^	703^[b]^	1.643(3)	320.5(2)
(tmg)_3_PTe (**3**)	−51.3^[b]^	1699^[b,d]^	1.642(2)	323.9(1)
(tmg)_3_PS (**4**)	30.6^[b]^		1.644(4)	319.7(2)
(tmg)_3_PCO_2_ (**5**)	−15.1^[c]^	181.9^[c]^	1.628(3)	332.3(2)
(tmg)_3_PSO_2_ (**6**)	−10.4^[c]^			
(tmg)_3_PO (**7**)	−6.5^[b]^		1.646(1)	314.6(1)
[(**1**)RhCl(cod)] (**8**)	49.9^[a]^	179.5^[a]^	1.666(2)	314.5(1)
[(**1**)AuCl] (**9**)	48.8^[a]^		1.654(2)	314.0(1)
[(**1**)PdCl(allyl)] (**10**)	60.0^[a]^

[a] measured in C_6_D_6_. [b] measured in THF‐d_8_. [c] measured in CD_2_Cl_2_. [d] ^1^ *J*(^31^P,^125^Te) coupling constant.

**Figure 4:**
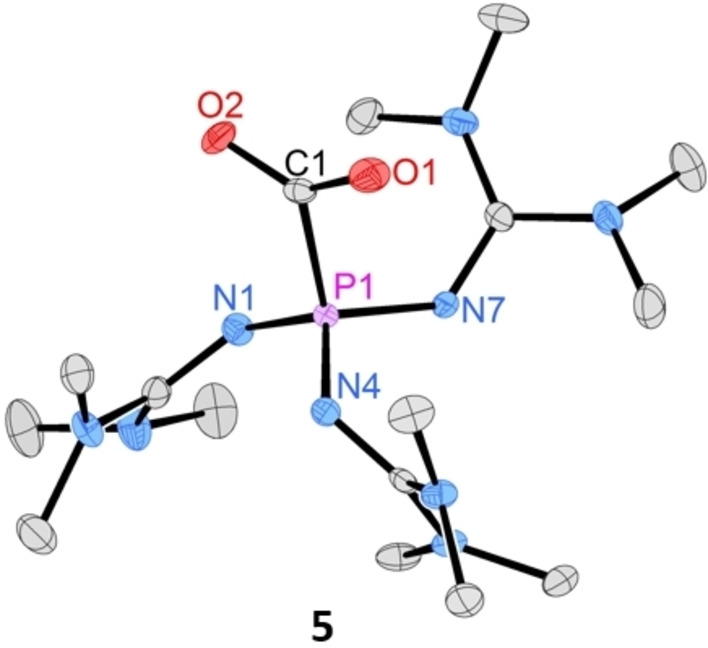
Molecular structure of phosphine‐CO_2_ adduct **5**. Hydrogen atoms are omitted for clarity; thermal ellipsoids are set at 50 % probability. Selected bond length [Å] and angles [°]: **5**: P1−N1: 1.628(3) P1−N4: 1.622(3), P1−N7: 1.633(3), P1−C1: 1.878(3), O2−C1: 1.241(4), O1−C1: 1.240(4), O1−C1−O2: 129.7(3).

Having the free phosphine **1** in hand, we revisited our recent studies on phosphine‐CO_2_ and phosphine‐SO_2_ adducts, where a close correlation between the stability of those adduct and the donor‐ability of the phosphine was identified.[^6^, ^7^, ^13^] Pressurizing a THF solution of phosphine **1** with 2 bar CO_2_ at room temperature resulted in the immediate precipitation of the phosphine‐CO_2_ adduct **5** as a white, crystalline solid in quantitative yield (Scheme 1). The ^31^P NMR spectrum of the phosphine‐CO_2_ adduct **5** shows a singlet at δ=−14.2 ppm, which is markedly upfield shifted compared to the resonance of **1** (δ=83.5 ppm) and the characteristic carboxylate signal appears at δ=171.3 ppm (^1^ *J*_PC_=181.9 Hz) in the ^13^C{^1^H} NMR spectrum. An XRD study of **5** confirmed the complexation of CO_2_ by the phosphorus atom with a P−C bond of 1.878(3) Å (Figure 4), which is in the same range to that of the CO_2_ adducts of **II** (1.882 Å) and **IV** (1.874 Å).[^3^, ^13^] The zwitterionic CO_2_ adduct **5** is stable at room temperature and does not carboxylate under reduced pressure (10^−3^ mbar). An ATR‐IR experiment confirmed the loss of CO_2_ at 60 °C under ambient pressure. Heating **5** to 60 °C under vacuum resulted in complete decarboxylation to give phosphine **1**, which was confirmed by ^31^P NMR spectroscopy. The reaction of **1** with 0.5 equivalents of DABSO (1,4‐Diazabicyclo[2.2.2]octane bis(sulfur dioxide) adduct)^[47]^ gave the SO_2_ adduct **6** in quantitative yield (Scheme 1). The ^31^P NMR spectrum of **6** shows a singlet at δ=−10.4 ppm, which is upfield shifted compared to the resonance of **1**. While **6** is stable in the solid state for days, storing a solution of **6** in dichloromethane for 3 h results in a mixture of different products, including phosphine oxide **7**, which suggests that sulfur monoxide is eliminated from the phosphine‐SO_2_ adduct (Figure S40).^[7]^

**Figure 5:**
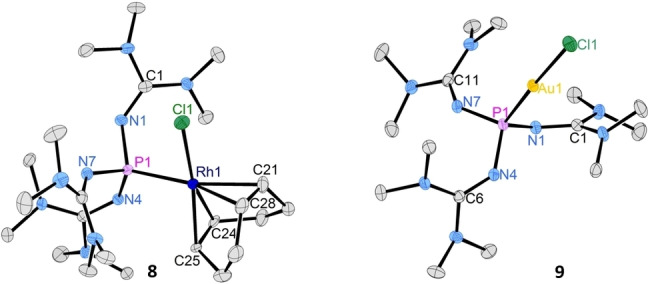
Molecular structures of phosphine‐rhodium complex **8** and phosphine‐gold complex **9**. Hydrogen atoms are omitted for clarity; thermal ellipsoids are set at 50 % probability. Selected bond length [Å] and angles [°]: **8**: P1−Rh1: 2.3198(5), Rh1−Cl1: 2.3829(5), P1−N1: 1.6497(15), P1−N4: 1.6779(15), P1−N7: 1.6710(16), N1−C1: 1.301(2), Rh1−C21: 2.2324(19), Rh1−C24: 2.108 (4), Rh1−C25: 2.094(4), Rh1−C28: 2.229(3); **9**: P1−Au1: 2.2524(5), Au1−Cl1: 2.3256(5), P1−N1: 1.6692(15), P1−N4: 1.6405(15), P1−N7: 1.6532(15), N1−C1: 1.304(2), N4−C6: 1.297(2), N7−C11: 1.301(2).

To explore the potential of phosphine **1** in coordination chemistry, we synthesized the Rh(I) complex [Rh(**1**)(cod)Cl] (**8**), the Au(I) complex [Au(**1**)Cl] (**9**) and Pd(II) complex [Pd(**1**)(allyl)Cl] (**10**) from common metal precursors (Scheme 1). The complexes were formed selectively and isolated as (off)‐white (**9**, **10**) or orange (**8**) solids in quantitative yield. Although the free phosphine **1** hydrolyses rapidly in the presence of water, a solution of complex **9** in wet THF shows no decomposition within 14 h at ambient conditions. As an alternative synthetic access to complexes **9** and **10**, the reaction of the stable phosphine‐CO_2_ adduct **5** with the respective metal precursors proceeds upon CO_2_ elimination. The ^31^P NMR resonances of complexes **8**–**9** appear at lower frequency than that of the free phosphine **1** (Table 3). The molecular structures of **8** and **9** were established by a single crystal XRD studies confirming that the new phosphine binds to the metal atoms by the phosphorus atom (Figure 5).

## Conclusion

In conclusion, a convenient and high‐yielding synthesis for tris(tetramethylguanidinyl)phosphine **1** has been developed. Previous difficulties associated with the isolation of the superbase were circumvented by using a phosphorus precursor with a built‐in auxiliary base, PCl(NMe_2_)_2_, and by carrying out the deprotonation step in a nonpolar solvent. The electron‐releasing character of the free phosphine is considerably affected by secondary interactions of the nitrogen atoms with Lewis acid sites. Therefore, the influence of solvents or other reactants must be taken into account when **1** is used as ligand in coordination chemistry and could also provide a means to remotely tune the ligand properties. Although several nitrogen donors are sterically accessible, the phosphorus atom represents the most reactive site in the coordination chemistry of **1**, as shown by the selective formation of Au^I^, Rh^I^ and Pd^II^ complexes, and the formation of zwitterionic Lewis base adducts with carbon dioxide and sulfur dioxide. In addition, the complete series of phosphine chalcogenides was prepared from the reaction of P(tmg)_3_ with N_2_O and the elemental chalcogens. In view of the easy synthesis from cheap and commercially available bulk precursors, the new phosphine has the potential to become a workhorse in stoichiometric reactions requiring extremely basic phosphorus(III) species.

## Experimental Section

**Synthesis of tris(tetramethylguanidinyl)phosphonium chloride 1⋅HCl**: A 1‐L two‐neck Schlenk flask equipped with an air‐cooled reflux condenser (*Dieldenser*)^[48]^ was charged with phosphorus trichloride (18.0 g, 131.0 mmol). The flask was cooled to 0 °C and tris(dimethylamino)phosphine (42.8 g, 262.0 mmol) was added to the stirred solution under permanent cooling, which resulted in the formation of PCl(NMe_2_)_2_ (^31^P NMR resonance in CDCl_3_: δ=164.2 ppm).^[49]^ To the same flask acetonitrile (180 mL) and subsequently 1,1,3,3‐Tetramethylguanidine (146.5 g, 1.29 mol) were added at room temperature. Note that the excess H(tmg) is necessary to achieve complete conversion. The reaction mixture was stirred for 3 hours at 120 °C. During that time, gaseous dimethylamine was formed, which was passed through an aqueous solution of hydrochloric acid connected to the upper outlet of the reflux condenser. After cooling the mixture to room temperature, the acetonitrile was removed *in vacuo* at ambient temperature. Subsequently, the excess H(tmg) was distilled from the residue at 120 °C under vacuum. The recovered H(tmg) is analytically pure and can be directly reused for a second batch if desired. **1**⋅HCl was obtained as an off‐white solid in quantitative yield (159.7 g, 389.6 mmol). m.p. 118–119 °C; ^1^H NMR (400 MHz, [D_3_]ACN): *δ*=7.83 (d, 1H, ^1^ *J*_P,H_=539 Hz; PH), 2.84 (s, 36 H; CH_3_); ^13^C NMR (101 MHz, [D_3_]ACN): *δ*=163.5 (d; N_2_CN), 40.5 (s; CH_3_); ^31^P NMR (162 MHz, [D_3_]ACN): *δ*=−17.6 (d, ^1^ *J*_PH_=539 Hz); HRMS (ESI): m/z calcd for C_15_H_37_N_9_P^+^: 374.29041 [M+H]^+^; found: 374.29026; elemental analysis calcd (%) for C_15_H_37_N_9_PCl: C 43.95, H 9.10, N 30.75; found: C 43.62, H 9.05, N 30.34.

**Synthesis of tris(tetramethylguanidinyl)phosphine 1**: Powdered **1**⋅HCl (2.92 g, 7.133 mmol) and KHMDS (1.42 g, 7.133 mmol) were suspended in *n*‐hexane (40 mL). The reaction mixture was stirred for 2 hours at 70 °C to achieve complete deprotonation. The precipitated KCl was filtered off and extracted once with *n*‐hexane (20 mL). The volatiles of the combined *n*‐hexane solutions were removed *in vacuo* to afford phosphine **1** as a beige oil that solidifies at room temperature (2.46 g, 6.587 mmol, 92 %). Note: If necessary, the phosphine can be purified by sublimation (100 °C, 1×10^−3^ mbar) or recrystallization from diethylether at −40 °C. m.p.: 74–75 °C; ^1^H NMR (400 MHz, [D_6_]benzene): *δ*=2.78 (s; CH_3_); ^13^C NMR (101 MHz, [D_6_]benzene): *δ*=157.4 (d; N_2_CN), 40.3 (s); ^31^P NMR (162 MHz, [D_6_]benzene): *δ*=83.5 (s); HRMS (ESI): m/z calcd for C_15_H_37_N_9_P^+^: 374.29041 [M+H]^+^; found 374.29020; elemental analysis: calcd (%) for C_15_H_36_N_9_P: C 48.24, H 9.72, N 33.75; found: C 47.62, H 9.47, N 32.75.

## Conflict of interest

The authors declare no conflict of interest.

1

## Supporting information

As a service to our authors and readers, this journal provides supporting information supplied by the authors. Such materials are peer reviewed and may be re‐organized for online delivery, but are not copy‐edited or typeset. Technical support issues arising from supporting information (other than missing files) should be addressed to the authors.

Supporting Information

## Data Availability

The data that support the findings of this study are available in the supplementary material of this article.
