# Supplementary material for: Tris(tetramethylguanidinyl)phosphine: The Simplest Non‐ionic Phosphorus Superbase and Strongly Donating Phosphine Ligand
Source: Chemistry. 2021 Dec 9;28(3):e202104021. doi: 10.1002/chem.202104021 (PMC9300019; doi:10.1002/chem.202104021)
Supplement: Supplementary file 1 — Supporting Information [file CHEM-28-0-s001.pdf]

Chemistry–A European Journal

Supporting Information

Tris(tetramethylguanidinyl)phosphine: The Simplest Non-ionic Phosphorus Superbase and Strongly Donating Phosphine Ligand

Florenz Buß, Maike B. Röthel, Janina A. Werra, Philipp Rotering, Lukas F. B. Wilm, Constantin G. Daniliuc, Pawel Löwe, and Fabian Dielmann*

CONTENTS:

Synthetic Details **3**

Preparation of compounds 1 – 10 **4**

One-pot procedure for tris(tetramethylguanidinyl)phosphonium chloride 1·HCl 4

Tris(tetramethylguanidinyl)phosphine 1 9

Procedure for the preparation of the [(1)Ni(CO)$_3$] complex 13

Tris(tetramethylguanidinyl)phosphine telluride 3 17

Tris(tetramethylguanidinyl)phosphine sulfide 4 21

Tris(tetramethylguanidinyl)phosphine–CO_2 adduct 5 23

Tris(tetramethylguanidinyl)phosphine–SO_2 adduct 6 29

Tris(tetramethylguanidinyl)phosphine oxide 7 32

Tris(tetramethylguanidinyl)phosphine–Rh complex 8 35

Tris(tetramethylguanidinyl)phosphine–Au complex 9 38

Tris(tetramethylguanidinyl)phosphine–Pd complex 10 41

Attempt for the determination of $pK_{a(MeCN)}$ of 1 43

X-ray Diffraction Studies **46**

Single-crystal X-ray structure analysis of 1: 47

Single-crystal X-ray structure analysis of 2: 48

Single-crystal X-ray structure analysis of 3: 49

Single-crystal X-ray structure analysis of 4: 50

Single-crystal X-ray structure analysis of 5: 51

Single-crystal X-ray structure analysis of 7: 52

Single-crystal X-ray structure analysis of 8: 53

Single-crystal X-ray structure analysis of 9: 54

DFT Calculations **55**

XYZ Data of the optimized structures **56**

References **67**

Synthetic Details

General remarks: Unless otherwise noted, all manipulations were performed under an inert atmosphere of dry argon, using standard Schlenk and drybox techniques. Dry and oxygen-free solvents were employed. All glassware was oven-dried at 160 °C prior to use. ^{1}H, ^{13}C, ^{77}Se, ^{125}Te and ^{31}P NMR spectra were recorded at 300 K on Agilent DD2 600, Bruker AVANCE I 400, Bruker AVANCE III 400 or Bruker AVANCE II 200 spectrometers. Chemical shifts are given in parts per million (ppm) relative to $SiMe_4$ (^{1}H, ^{13}C), 85% H_3PO_4 (^{31}P), Me_2Se (^{77}Se), Me_2Te (90% in C_6D_6, ^{125}Te) and they were referenced to the residual solvent signals (C_6D_6: ^{1}H δ_H = 7.16, ^{13}C δ_C = 128.06; CD_3CN: δ_H = 1.94, ^{13}C δ_C = 118.26; toluene-d_8: ^{1}H δ_H = 2.09, ^{13}C δ_C = 20.40; THF-d_8: ^{1}H δ_H = 1.73, ^{13}C δ_C = 67.57) or internally by the instrument after locking and shimming to the deuterated solvent (^{31}P, ^{77}Se, ^{125}Te). Chemical shifts (δ) are reported in ppm. NMR multiplicities are abbreviated as follows: s = singlet, d = doublet, t = triplet, p = pentet, sept = septet, m = multiplet, br = broad signal. Mass spectrometry was recorded using an Orbitrap LTQ XL (Thermo Scientific) spectrometer. IR spectra were obtained on a Bruker Alpha Spectrometer. Melting points were determined on a Stuart SMP20.

Reagents and Handling: All compounds were purchased from commercial sources (Sigma Aldrich, Alfa Aesar, abcr GmbH, Strem Chemicals) and used as received if not stated otherwise. 1,1,3,3-Tetramethylguanidine was dried over CaH_2 prior to distillation. Phosphorous trichloride was distilled prior use. Carbon dioxide was purchased from Westfalen AG (Münster) as CO_2 4.5 (99.995%). Nitrous oxide was purchased from Westfalen AG (Münster) as N_2O 4.5 (99.995%). [(pyrr)$_3$PCH$_2$Ph][OTf],[1] [AuCl(tht)][2] and [{Pd(allyl)Cl}$_2$][3] were prepared following literature procedures.

Preparation of compounds **1** – **10**

Synthesis of tris(tetramethylguanidinyl)phosphonium chloride 1·HCl: A 1-L two-neck Schlenk flask equipped with an air-cooled reflux condenser (*Dieldenser*, German patent No. DE 102017124711 A1 20190425) was charged with phosphorus trichloride (18.0 g, 131.0 mmol). The flask was cooled to 0 °C and tris(dimethylamino)phosphine (42.8 g, 262.0 mmol) was added to the stirred solution under permanent cooling, which resulted in the formation of $PCl(NMe_2)_2$ (^{31}P NMR resonance in $CDCl_3$: δ = 164.2 ppm)[4]. To the same flask acetonitrile (180 mL) and subsequently 1,1,3,3-Tetramethylguanidine (146.5 g, 1.29 mol) were added at room temperature. Note that the excess H(tmg) is necessary to achieve complete conversion. The reaction mixture was stirred for 3 hours at 120 °C. During that time, gaseous dimethylamine is formed, which was passed through an aqueous solution of hydrochloric acid connected to the upper outlet of the reflux condenser. After cooling the mixture to room temperature, the acetonitrile was removed *in vacuo* at ambient temperature. Subsequently, the excess H(tmg) was distilled from the residue at 120 °C under vacuum. The recovered H(tmg) is analytically pure and can be directly reused for a second batch if desired. **1**·HCl was obtained as an off-white solid in quantitative yield (159.7 g, 389.6 mmol). **1**·HCl is soluble in acetonitrile and moderatly soluble in THF. It must be stored in the absence of moisture because it is highly hygroscopic and slowly decomposes in the presence of water.

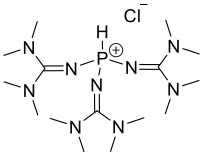

^{1}H NMR (400 MHz, 300 K, MeCN-d_3): δ = 7.83 (d, 1 H, $^1J_{PH}$ = 539 Hz, PH), 2.84 (s, 36 H, CH_3).

^{13}C{^{1}H} NMR (100.6 MHz, 300 K, MeCN-d_3): δ = 163.5 (d, N_2CN), 40.5 (s, CH_3).

^{31}P NMR (161.9 MHz, 300 K, MeCN-d_3): δ = –17.6 (d, $^1J_{PH}$ = 539 Hz).

^{31}P{^{1}H} NMR (161.9 MHz, 300 K, MeCN-d_3): δ = –17.6 (s).

HRMS (ESI): m/z calculated for $[C_{15}H_{37}N_9P]^+$ $(M+H)^+$ 374.29041, found 374.29026.

CHN analysis: calcd. for $C_{15}H_{37}N_9PCl$: C, 43.95; H, 9.10; N, 30.75. Found: C, 43.62; H, 9.05; N, 30.34.

Melting point: 118–119 °C.

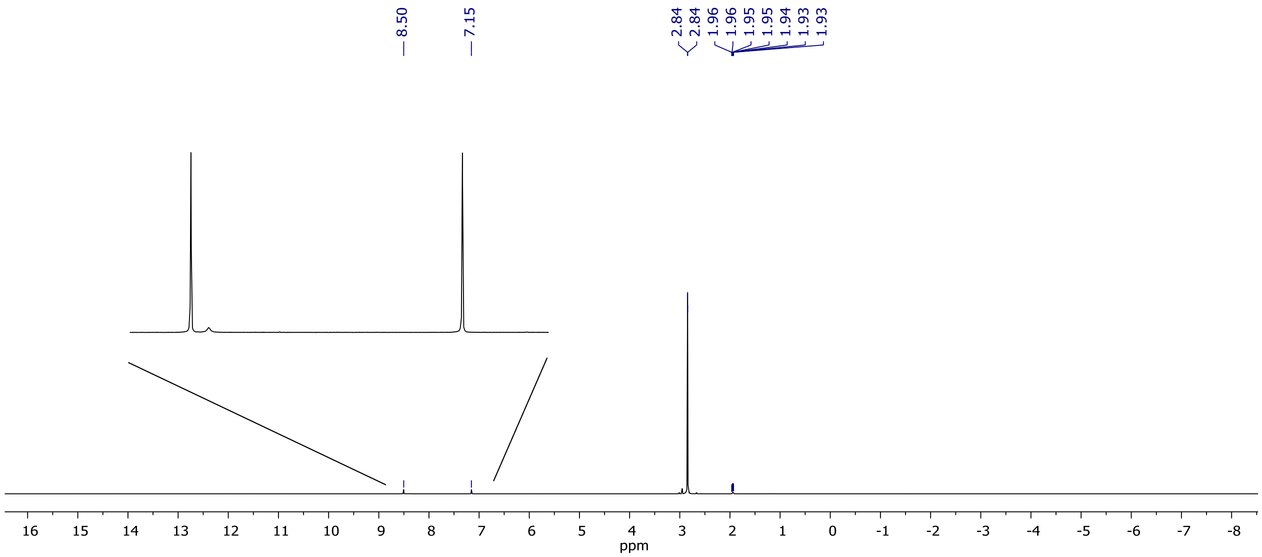

Figure S1: ^{1}H NMR spectrum (MeCN-d_3, 300 K, 400 MHz) of **1**·HCl.

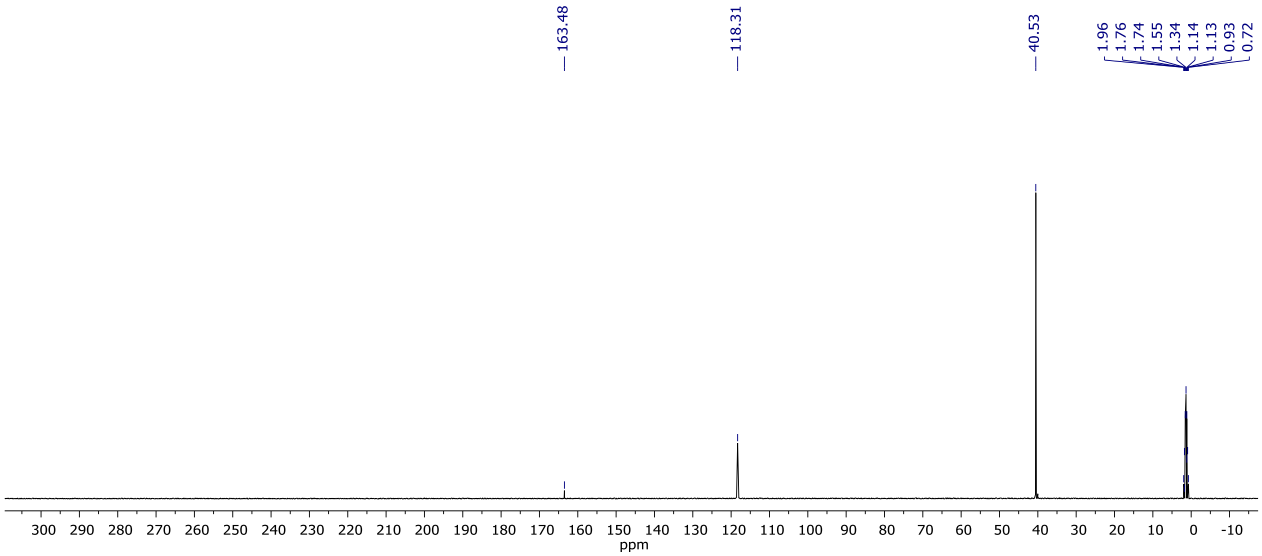

Figure S2: ^{13}C{^{1}H} NMR spectrum (MeCN-d_3, 300 K, 101 MHz) of **1**·HCl.

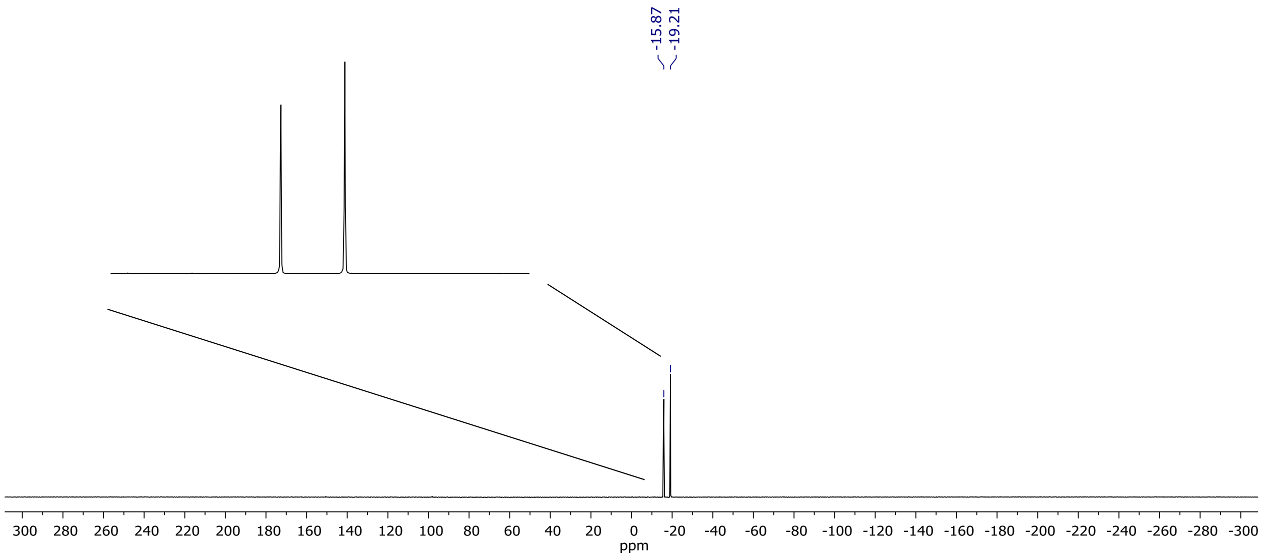

Figure S3: ^{31}P NMR spectrum (MeCN-d_3, 300 K, 162 MHz) of **1**·HCl.

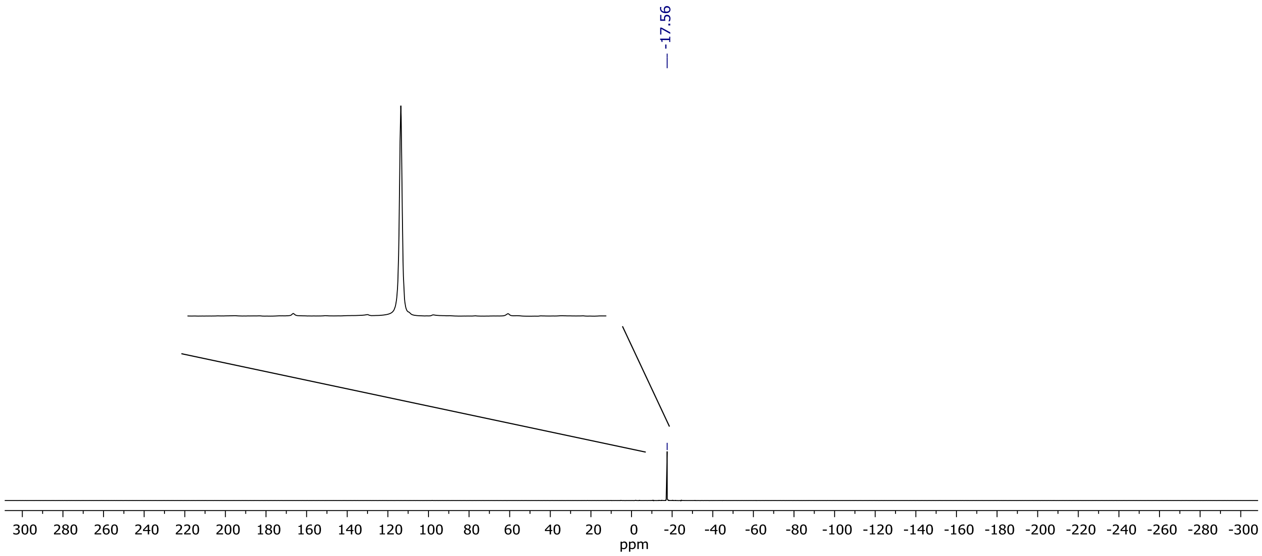

Figure S4: ^{31}P{^{1}H} NMR spectrum (MeCN-d_3, 300 K, 162 MHz) of **1**·HCl.

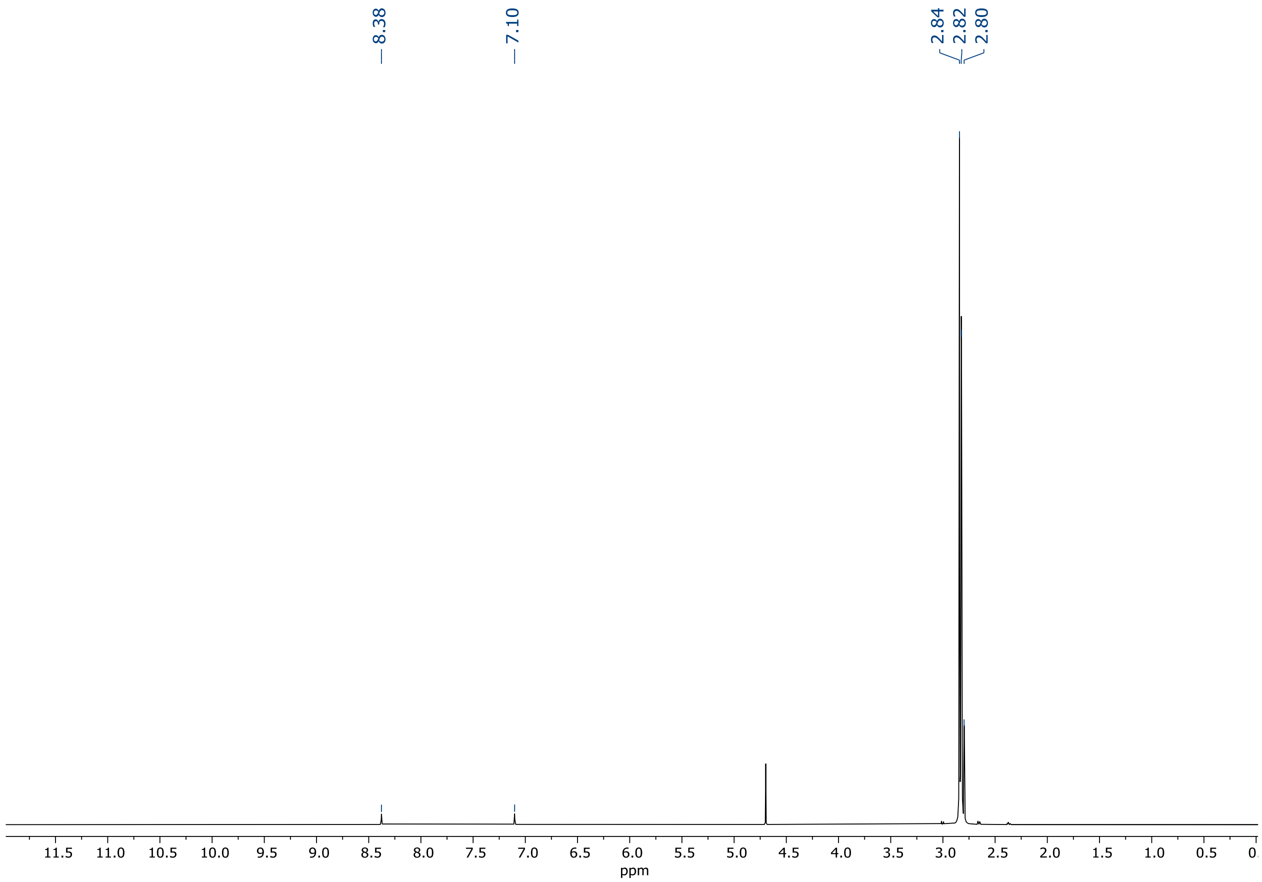

Figure S 5: ^{1}H NMR spectrum (D_2O, 300 K, 400 MHz) measured after 30 minutes showing the hydrolysis of **1**·HCl.

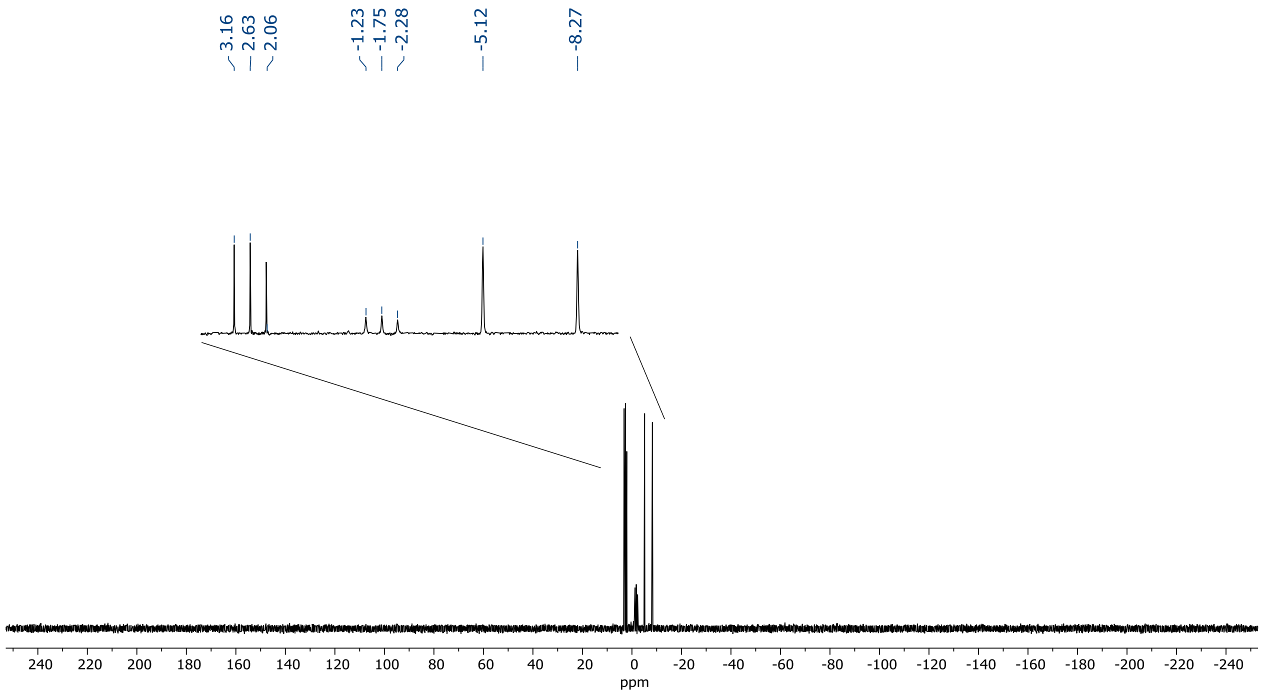

Figure S 6: ^{31}P NMR spectrum (D_2O, 300 K, 162 MHz) measured after 30 minutes showing the hydrolysis of **1**·HCl.

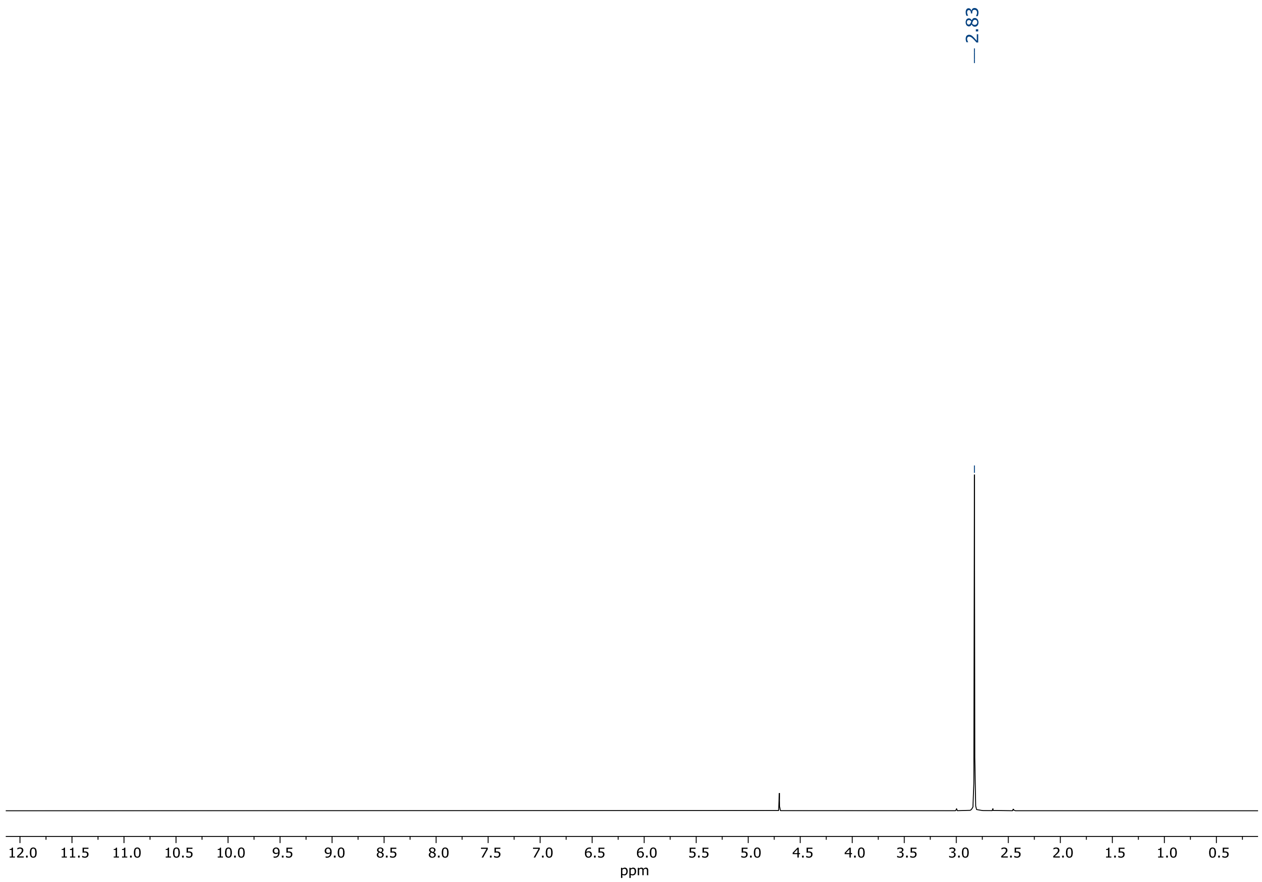

Figure S 7: ^{1}H NMR spectrum (D_2O, 300 K, 400 MHz) measured after 20 hours showing the hydrolysis of **1**·HCl.

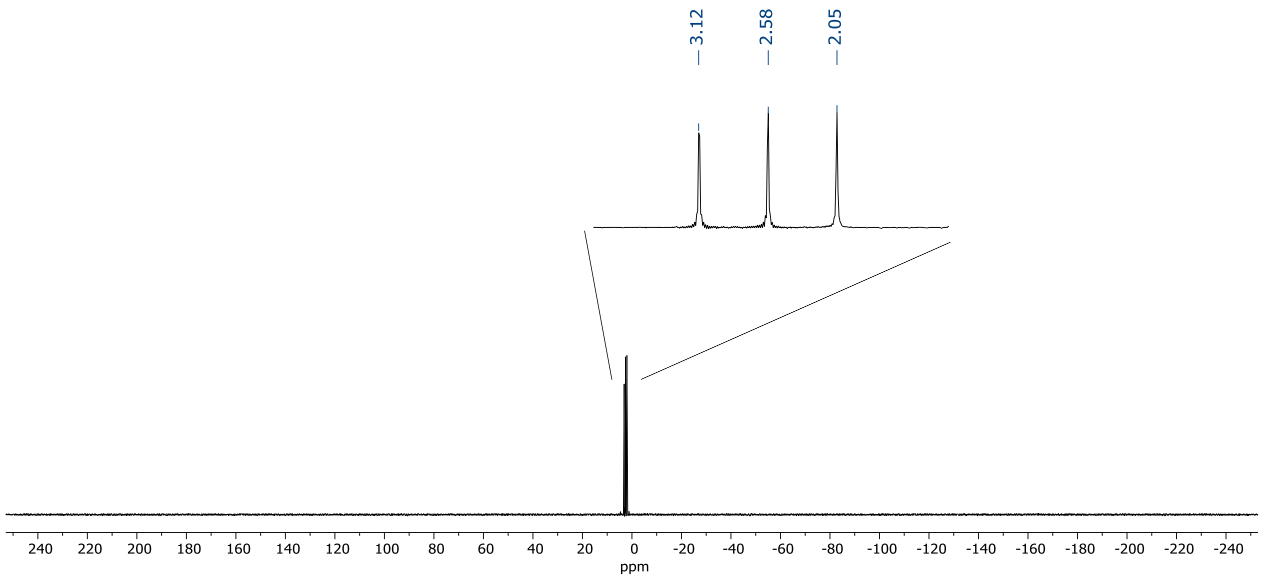

Figure S 8: ^{31}P NMR spectrum (D_2O, 300 K, 162 MHz) measured after 20 hours showing the hydrolysis of **1**·HCl.

Tris(tetramethylguanidinyl)phosphine 1:

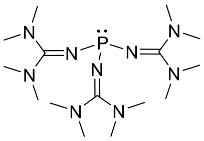

Powdered **1**·HCl (2.92 g, 7.133 mmol) and KHMDS (1.42 g, 7.133 mmol) were suspended in *n*-hexane (40 mL). The reaction mixture was stirred for 2 hours at 70 °C to achieve complete deprotonation. The precipitated KCl was filtered off and extracted once with *n*-hexane (20 mL). The volatiles of the combined *n*-hexane solutions were removed *in vacuo* to afford phosphine **1** as a beige oil that solidifies at room temperature (2.46 g, 6.587 mmol, 92%). *Note: If necessary, the phosphine can be purified by sublimation (100 °C, 1x10^{-3} mbar) or recrystallization from diethylether at -40 °C.*

Phosphine **1** is soluble in diethyl ether, *n*-hexane, *n*-pentane, toluene, THF, decomposes slowly in acetonitrile and decomposes rapidly in dichloromethane and chloroform. Single crystals were obtained by storing an *n*-hexane solution of **1** at –40 °C (*vide infra*). Phosphine **1** can be stored in the absence of air and moisture for months.

^{1}H NMR (400 MHz, 300 K, C_6D_6): δ = 2.78 (s, CH_3).

^{13}C{^{1}H} NMR (100.6 MHz, 300 K, C_6D_6): δ = 157.4 (d, N_2CN), 40.3 (s).

^{31}P NMR (161.9 MHz, 300 K, C_6D_6): δ = 83.5 (s).

^{31}P{^{1}H} NMR (161.9 MHz, 300 K, C_6D_6): δ = 83.5 (s).

^{31}P NMR (161.9 MHz, 300 K, MeCN-d_3): δ = 93.1 (s).

HRMS (ESI): m/z calculated for $[C_{15}H_{37}N_9P]^+$ $(M+H)^+$ 374.29041, found 374.29020.

CHN analysis: calcd. for $C_{15}H_{36}N_9P$: C, 48.24; H, 9.72; N, 33.75. Found: C, 47.62; H, 9.47; N, 32.75.

Melting point: 74–75 °C.

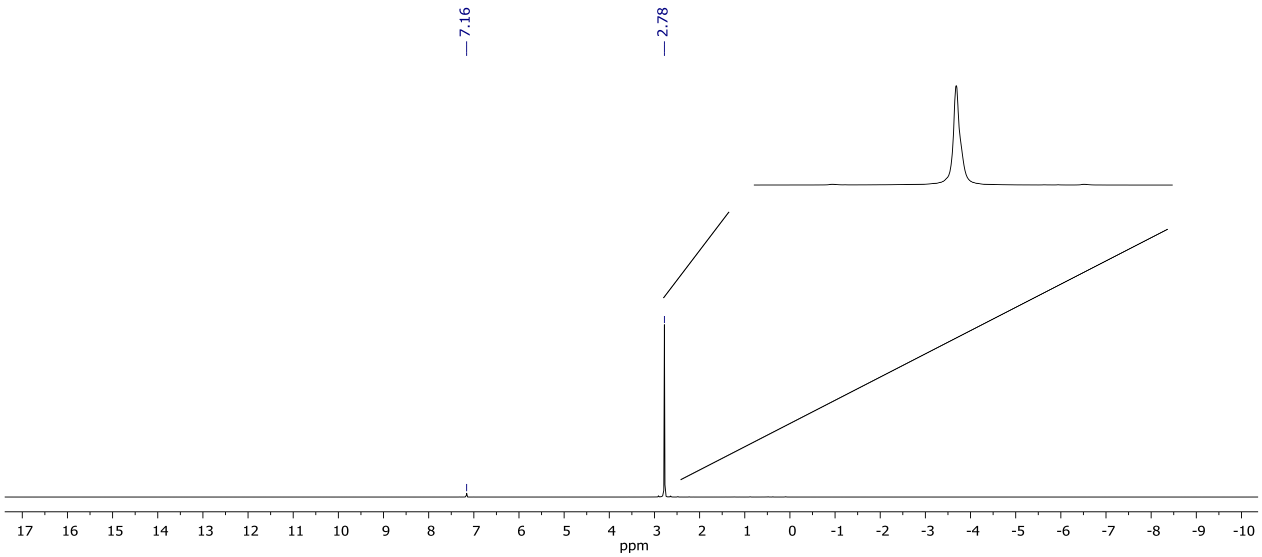

Figure S9: ^{1}H NMR spectrum (C_6D_6, 300 K, 400 MHz) of **1**.

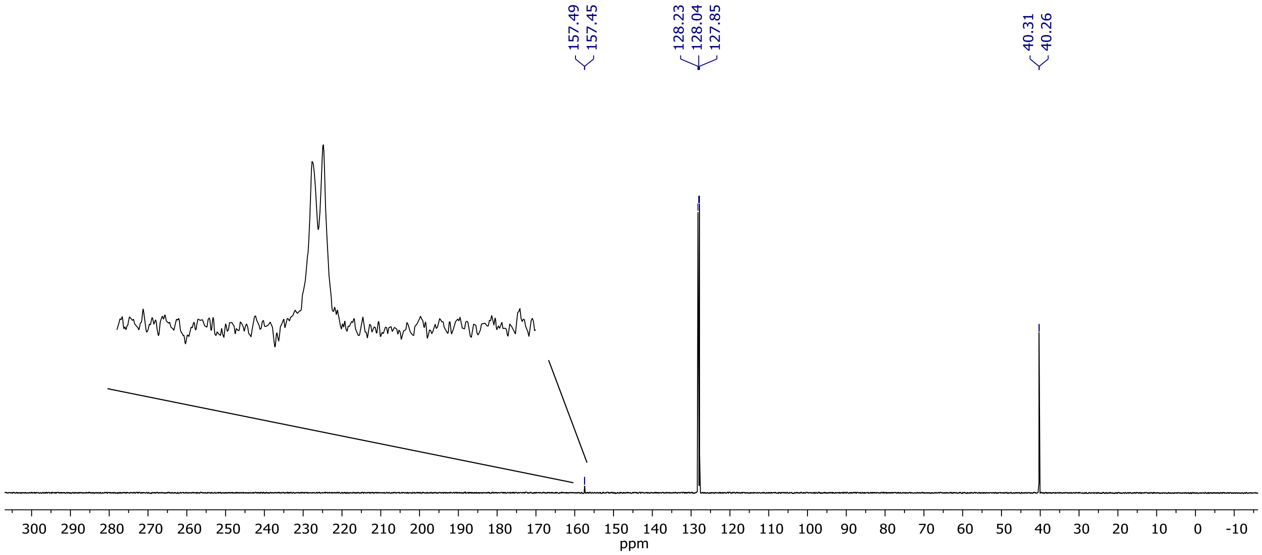

Figure S10: ^{13}C{^{1}H} NMR spectrum (C_6D_6, 300 K, 101 MHz) of **1**.

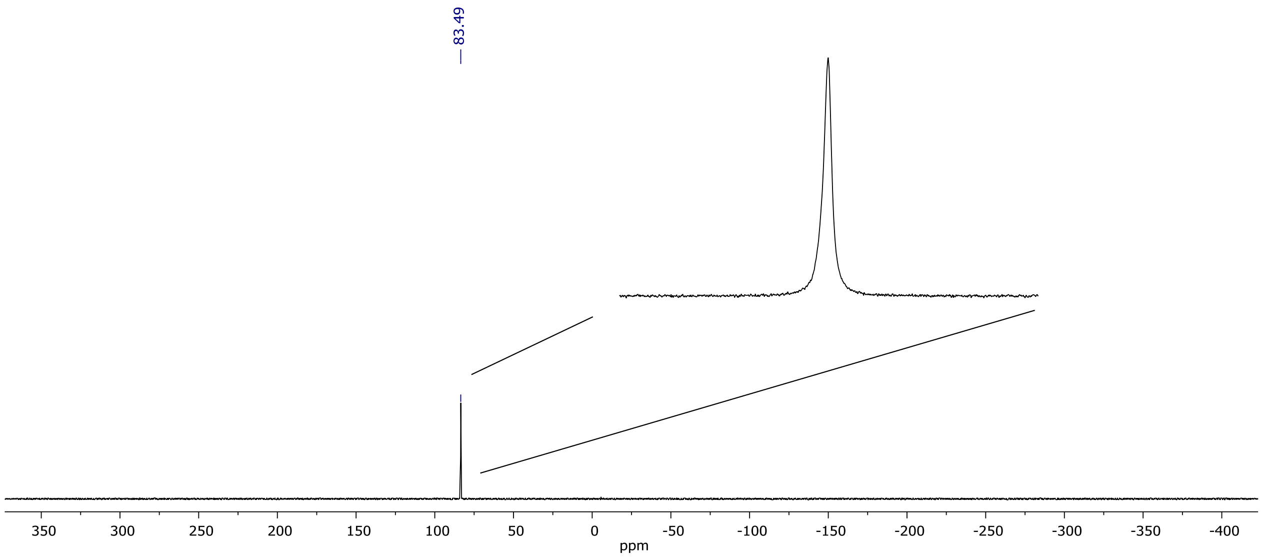

Figure S11: ^{31}P NMR spectrum (C_6D_6, 300 K, 162 MHz) of **1**.

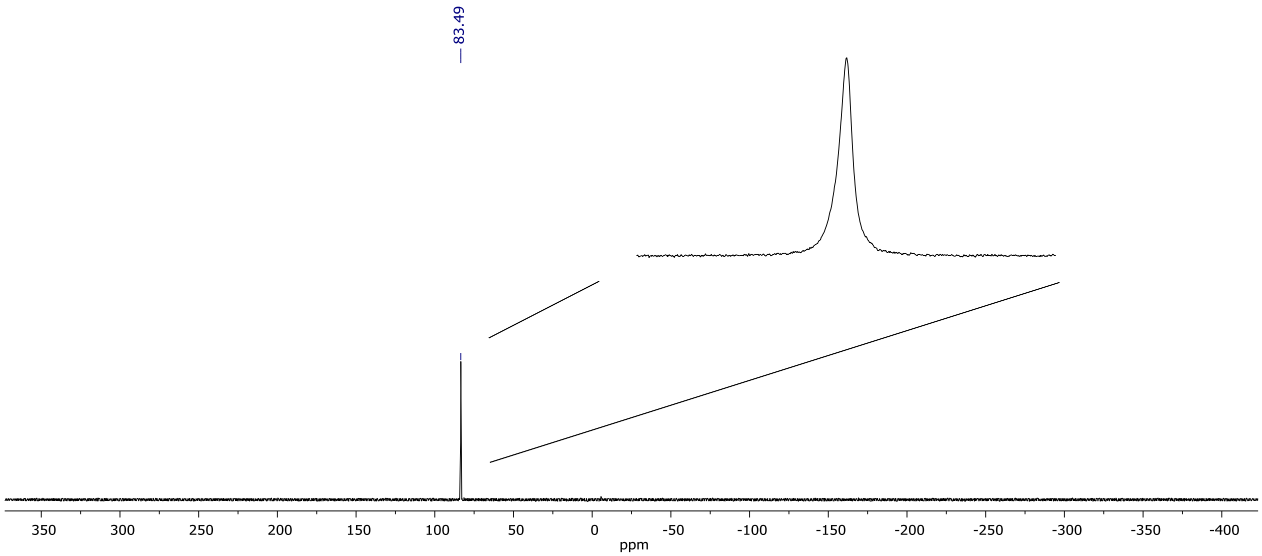

Figure S12: ^{31}P{^{1}H} NMR spectrum (C_6D_6, 300 K, 162 MHz) of **1**.

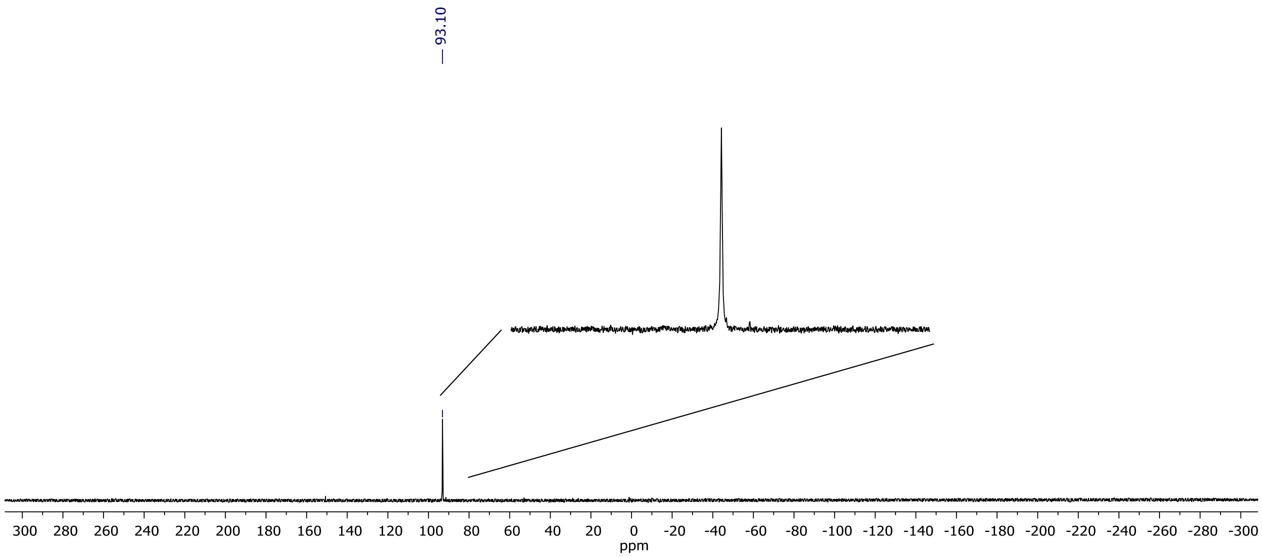

Figure S13: $^{31}P\{^{1}H\}$ NMR spectrum (MeCN-d_3, 300 K, 162 MHz) of **1** measured after 1 hour.

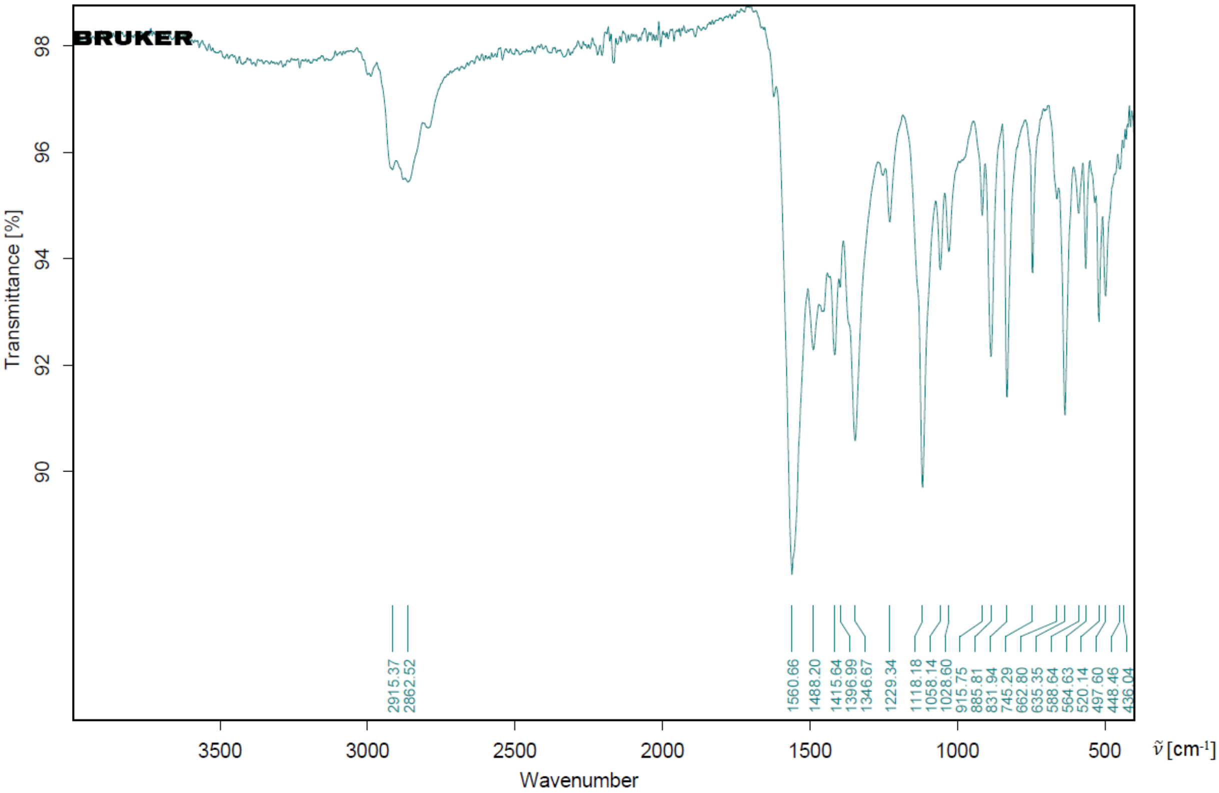

Figure S 14: FT-IR spectrum of **1** (solid).

Procedure for the preparation of the [(1)Ni(CO)$_3$] complex:

A standard solution of Ni(CO)$_4$ (2 mL, 0.2 M in toluene, 0.4 mmol) was added to phosphine **1** (145 mg, 0.39 mmol) and the resulting solution was stirred for 10 minutes at room temperature. The volatiles were removed *in vacuo*. The solid residue was analyzed by IR spectroscopy: in neat form, and dissolved in toluene, THF, dichloromethane and methanol, respectively.

A_1 CO stretching frequency of [(**1**)Ni(CO)$_3$] neat: 2036.5 cm^{-1}

A_1 CO stretching frequency of [(**1**)Ni(CO)$_3$] in toluene: 2041.1 cm^{-1}

A_1 CO stretching frequency of [(**1**)Ni(CO)$_3$] in THF: 2048.6 cm^{-1}

A_1 CO stretching frequency of [(**1**)Ni(CO)$_3$] in dichloromethane: 2049.1 cm^{-1}

A_1 CO stretching frequency of [(**1**)Ni(CO)$_3$] in methanol: 2054.1 cm^{-1}

Tris(tetramethylguanidinyl)phosphine selenide 2: Phosphine **1** (500 mg, 1.34 mmol, 1 eq.) and selenium (126 mg, 1.74 mmol, 1.3 eq.) were suspended in THF (8 mL). After stirring for 4 hours at room temperature the excess of selenium was filtered off. After evaporation to dryness *in vacuo*, **2** was isolated as a greenish solid in quantitative yield. Single crystals as colorless plates were obtained by heating **2** for 3 hours in diethyl ether at 80 °C in a pressure tube and slowly cool down the mixture to room temperature (*vide infra*).

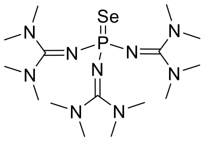

^{1}H NMR (400 MHz, 300 K, THF-d_8): δ = 2.86 (s, CH_3).

^{13}C{^{1}H} NMR (100.6 MHz, 300 K, THF-d_8): δ = 159.7 (d, N_2CN), 40.5 (s).

^{31}P NMR (161.9 MHz, 300 K, THF-d_8): δ = 13.2 ($^{1}J_{PSe}$ = 703 Hz).

^{77}Se NMR (76.3 MHz, 300 K, THF-d_8): δ = 14.5 (d, $^{1}J_{PSe}$ = 703 Hz).

HRMS (ESI): m/z calculated for $[C_{15}H_{37}N_9PSe]^+$ $(M+H)^+$ 454.20698, found 454.20692.

CHN analysis: calcd. for $C_{15}H_{36}N_9PSe$: C, 39.82; H, 8.02; N, 27.86. Found: C, 39.96; H, 7.74; N, 27.58.

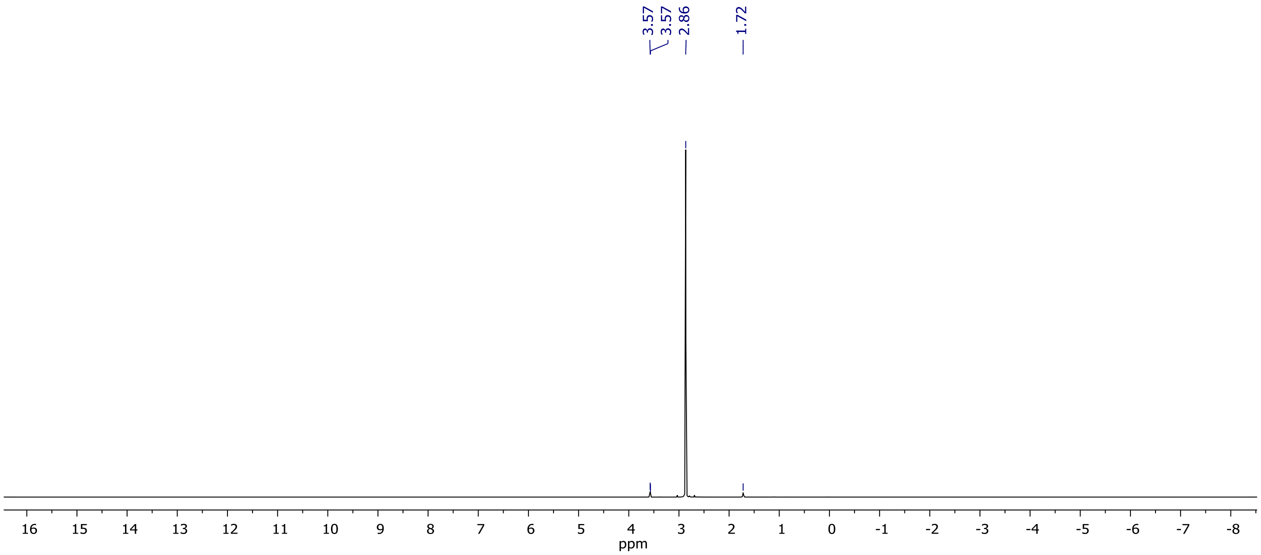

Figure S15: ^{1}H NMR spectrum (THF-d_8, 300 K, 400 MHz) of **2**.

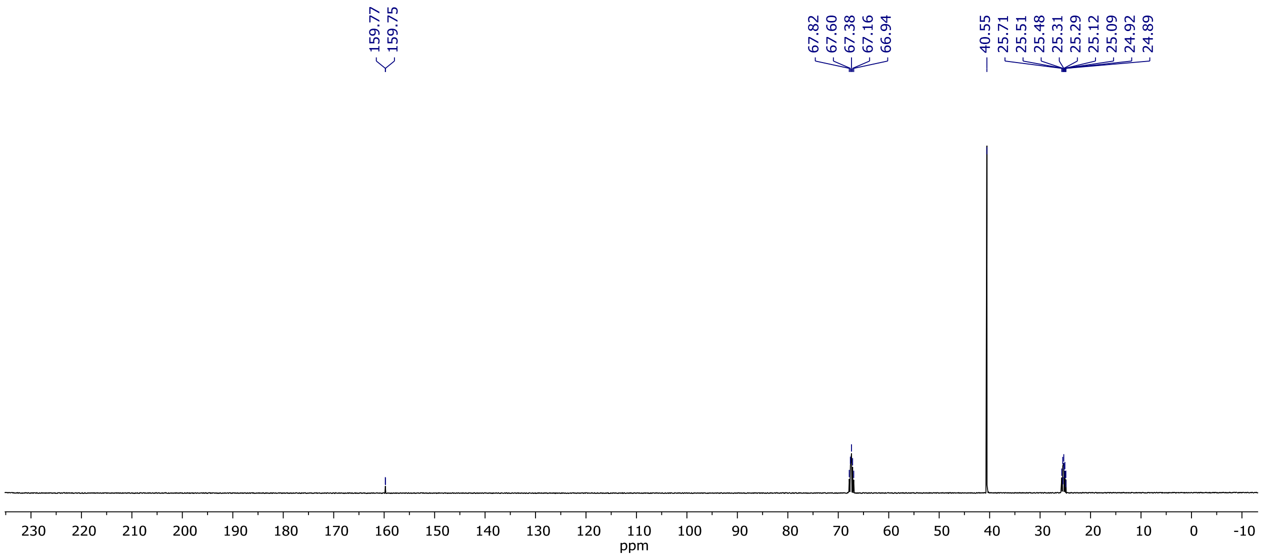

Figure S16: $^{13}C\{^{1}H\}$ NMR spectrum (THF-d_8, 300 K, 101 MHz) of **2**.

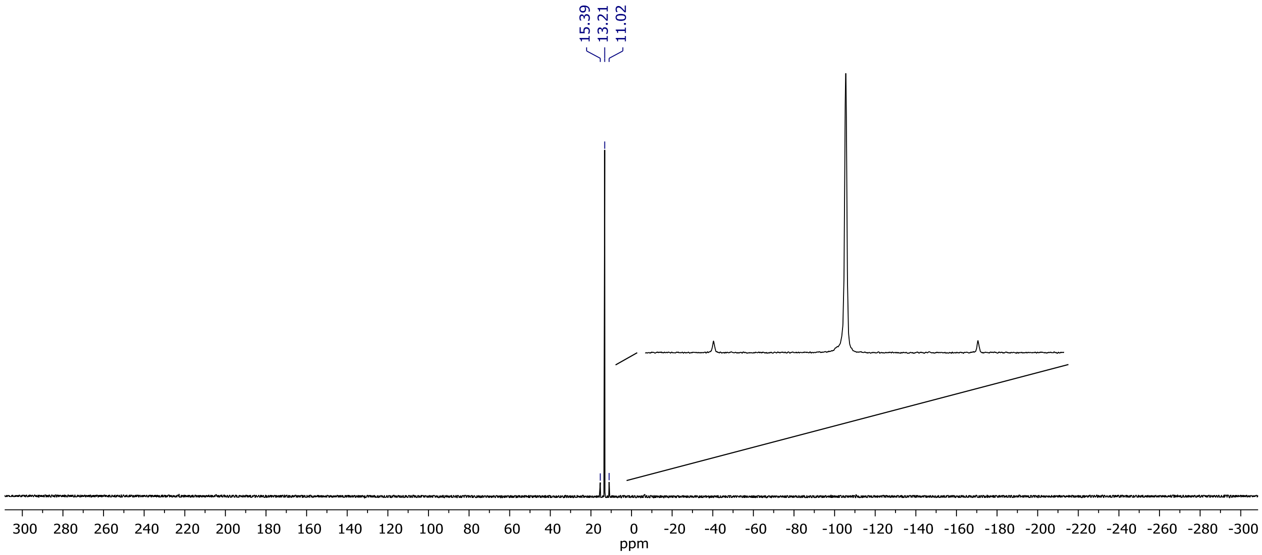

Figure S17: ^{31}P NMR spectrum (THF-d_8, 300 K, 162 MHz) of **2**.

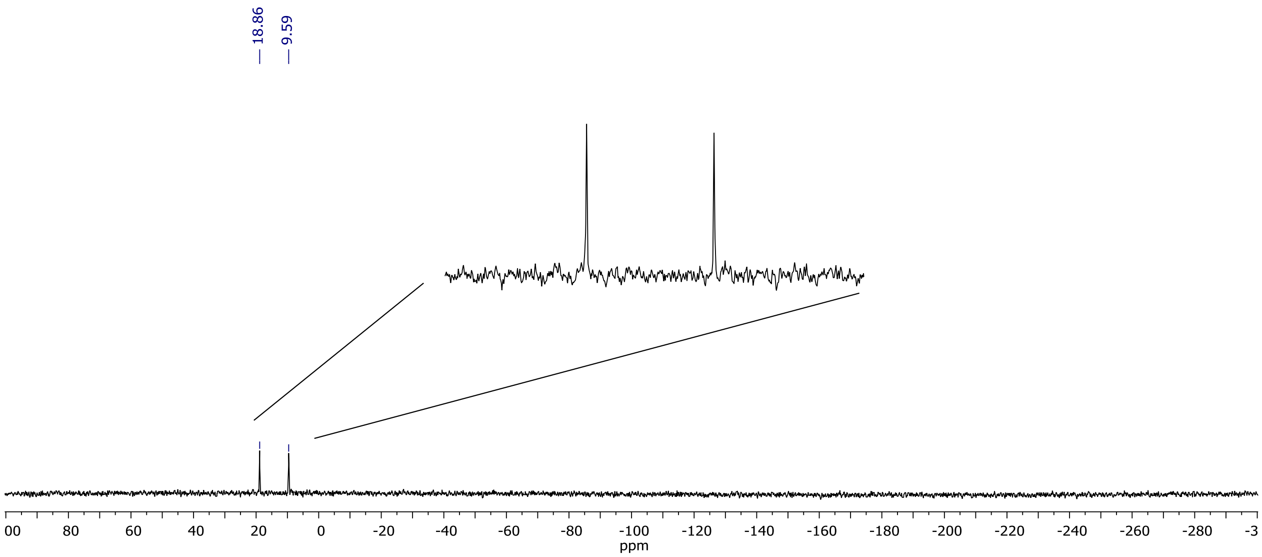

Figure S18: ^{77}Se NMR spectrum (THF-d_8, 300 K, 76.3 MHz) of **2**.

Tris(tetramethylguanidinyl)phosphine telluride 3

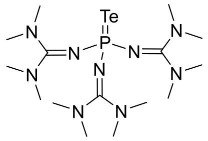

Phosphine **1** (250 mg, 0.67 mmol) and tellurium (95 mg) were suspended in THF (8 mL). After stirring for 2 hours at 80 °C the excess of tellurium was filtered off. After evaporation to dryness *in vacuo*, **3** was isolated as a yellowish solid in quantitative yield. Single crystals as yellow plates were obtained by diffusion of *n*-hexane into a THF solution of **3** (*vide infra*).

^{1}H NMR (400 MHz, 300 K, THF-d_8): δ = 2.89 (s, CH_3).

^{13}C{^{1}H} NMR (100.6 MHz, 300 K, THF-d_8): δ = 160.2 (d, N_2CN), 40.9 (s).

^{31}P NMR (161.9 MHz, 300 K, THF-d_8): δ = –51.3 ($^{1}J_{PTe}$ = 1699 Hz).

^{125}Te NMR (76.3 MHz, 300 K, THF-d_8): δ = –79.9 (d, $^{1}J_{PTe}$ = 1699 Hz).

HRMS (ESI): m/z calculated for $[C_{10}H_{24}N_6PTe]^+$ (M-tmg)$^+$ 389.0857, found 389.0855.

Melting Point: 123.0 °C

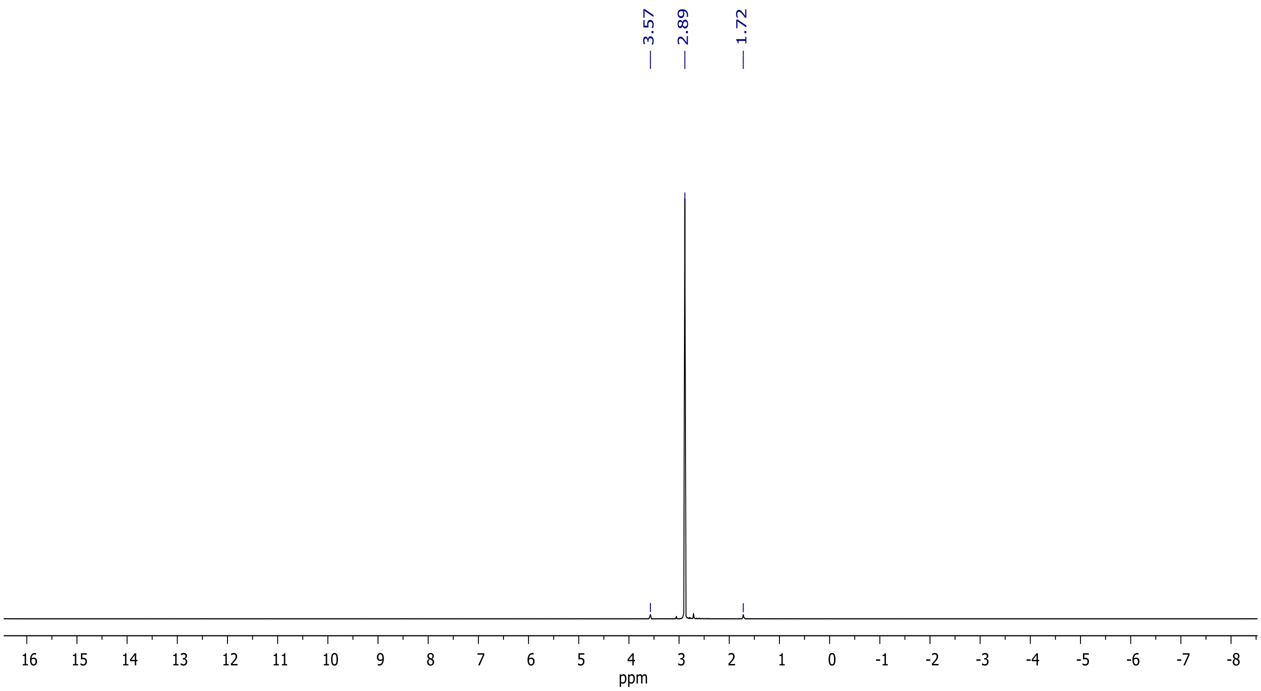

Figure S19: ^{1}H NMR spectrum (THF-d_8, 300 K, 400 MHz) of **3**.

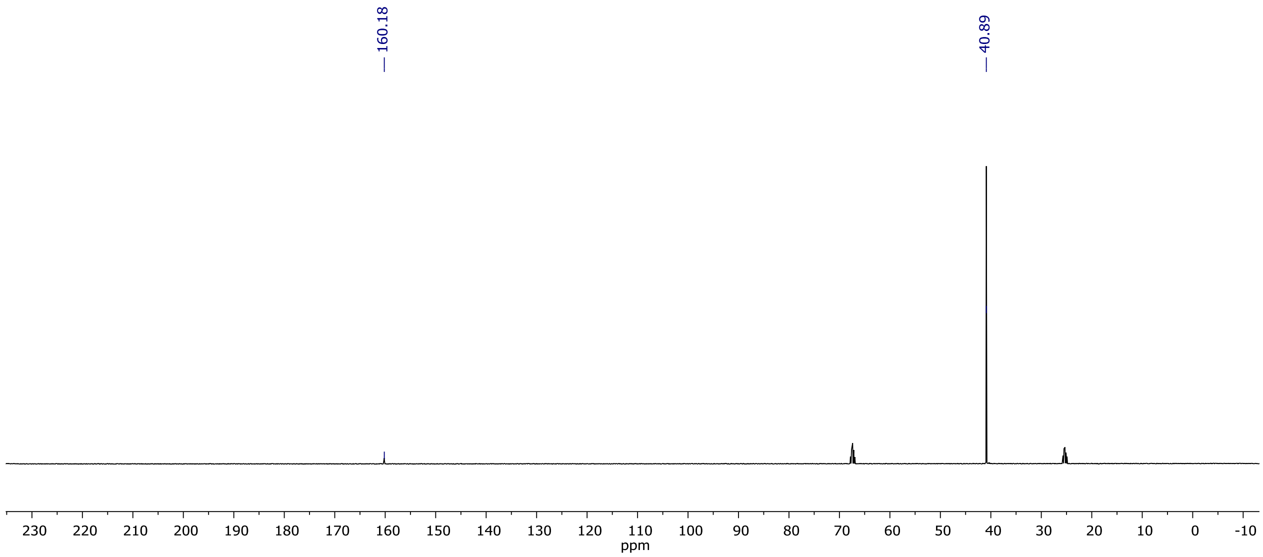

Figure S20: $^{13}C\{^{1}H\}$ NMR spectrum (THF-d_8, 300 K, 101 MHz) of **3**.

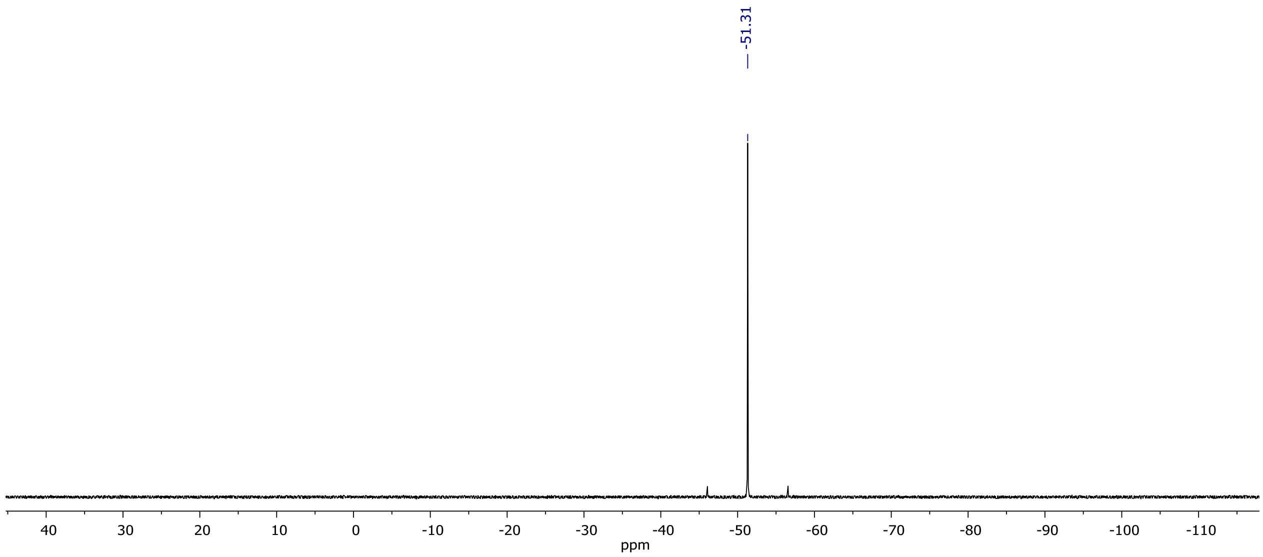

Figure S21: ^{31}P NMR spectrum (THF-d_8, 300 K, 162 MHz) of **3**.

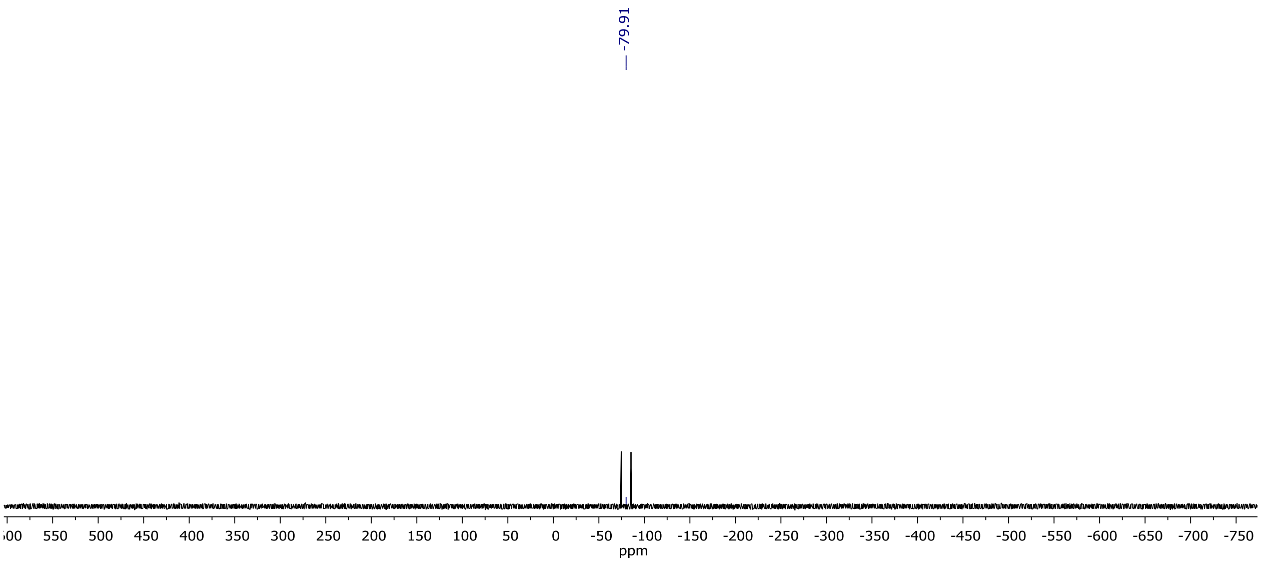

Figure S22: ^{125}Te NMR spectrum (THF-d_8, 300 K, 76.3 MHz) of **3**.

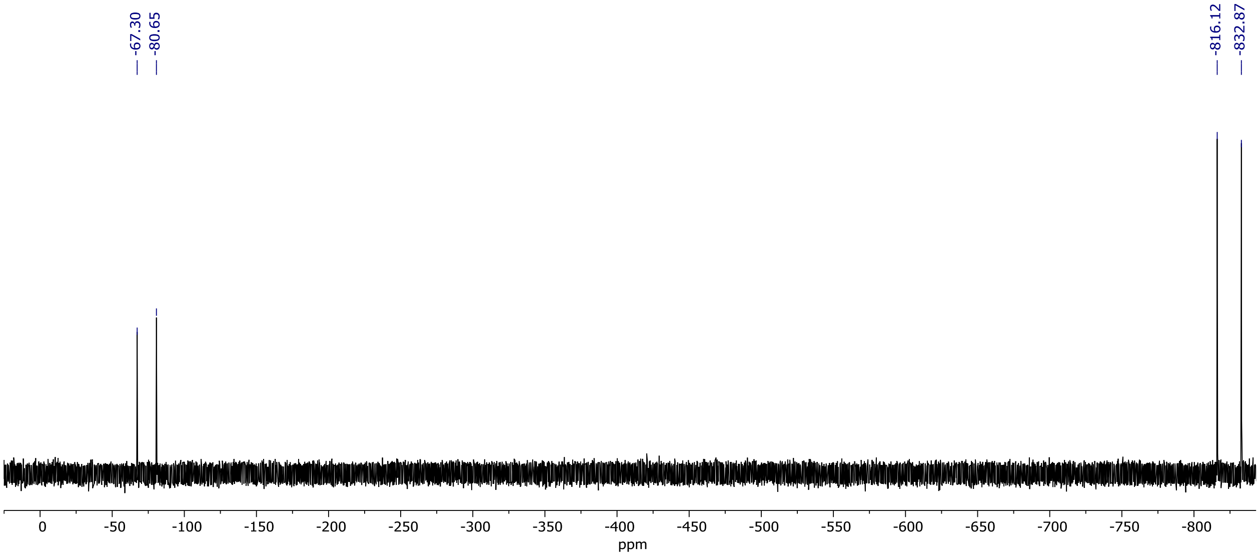

Figure S 23: ^{125}Te NMR spectrum (C_6D_6, 300 K, 126.2 MHz) of **3** and $TeP(NMe_2)_3$ as internal reference.

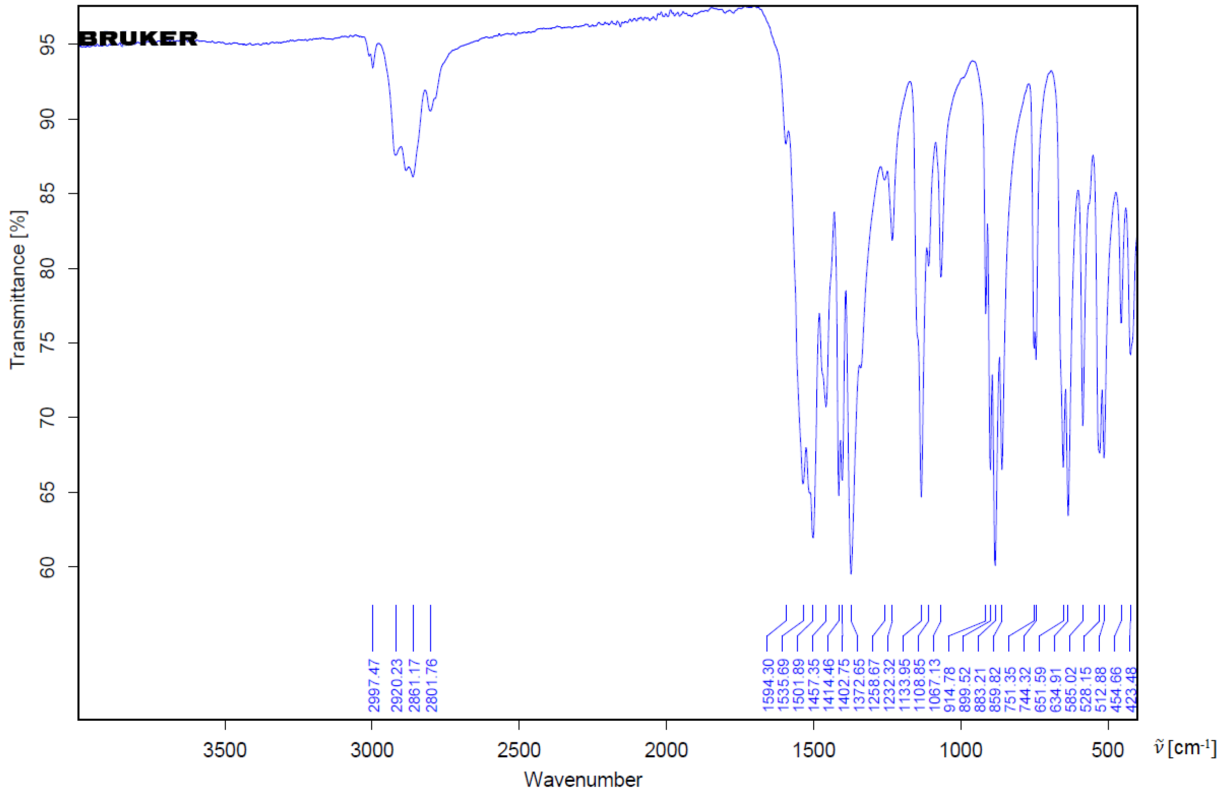

Figure S 24: FT-IR spectrum of **3** (solid).

Tris(tetramethylguanidinyl)phosphine sulfide 4

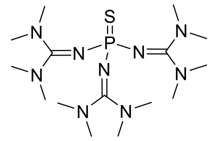

Phosphine **1** (250 mg, 0.67 mmol) and sulfur (35 mg) were suspended in THF (8 mL). After stirring for 5 hours at room temperature *n*-hexane was added to the mixture and the resulting white precipitate was filtered off. After evaporation to dryness *in vacuo*, **4** was isolated as a colorless solid in quantitative yield. Single crystals as colorless plates were obtained by diffusion of *n*-hexane into a THF solution of **4** (*vide infra*).

^{1}H NMR (400 MHz, 300 K, THF-d_8): δ = 2.84 (s, CH_3).

^{13}C{^{1}H} NMR (100.6 MHz, 300 K, THF-d_8): δ = 159.3 (d, N_2CN), 40.5 (s).

^{31}P NMR (161.9 MHz, 300 K, THF-d_8): δ = 30.6 (s).

HRMS (ESI): m/z calculated for $[C_{15}H_{37}N_9PS]^+$ $(M+H)^+$ 406.26248, found 406.25795.

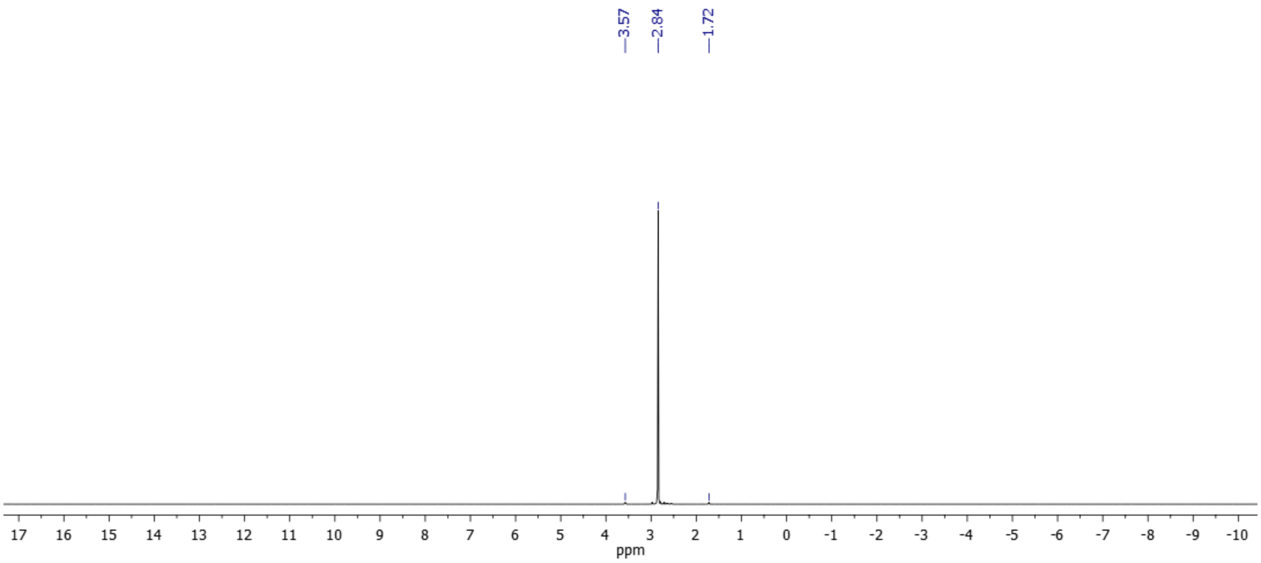

Figure S25: ^{1}H NMR spectrum (THF-d_8, 300 K, 400 MHz) of **4**.

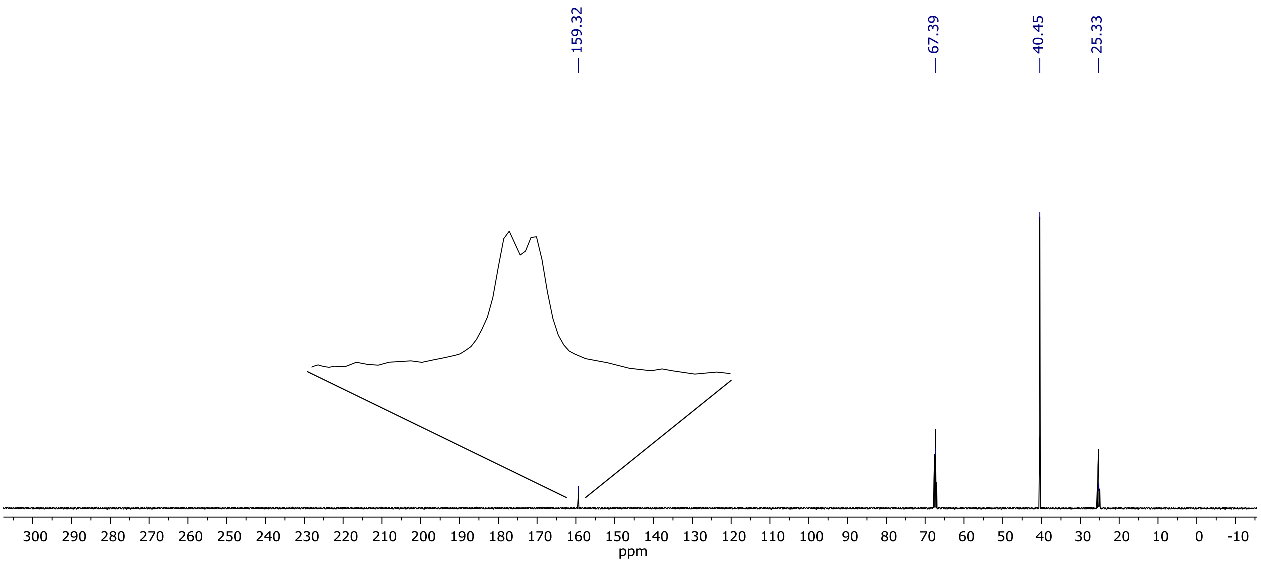

Figure S26: $^{13}C\{^1H\}$ NMR spectrum (THF-d_8, 300 K, 101 MHz) of **4**.

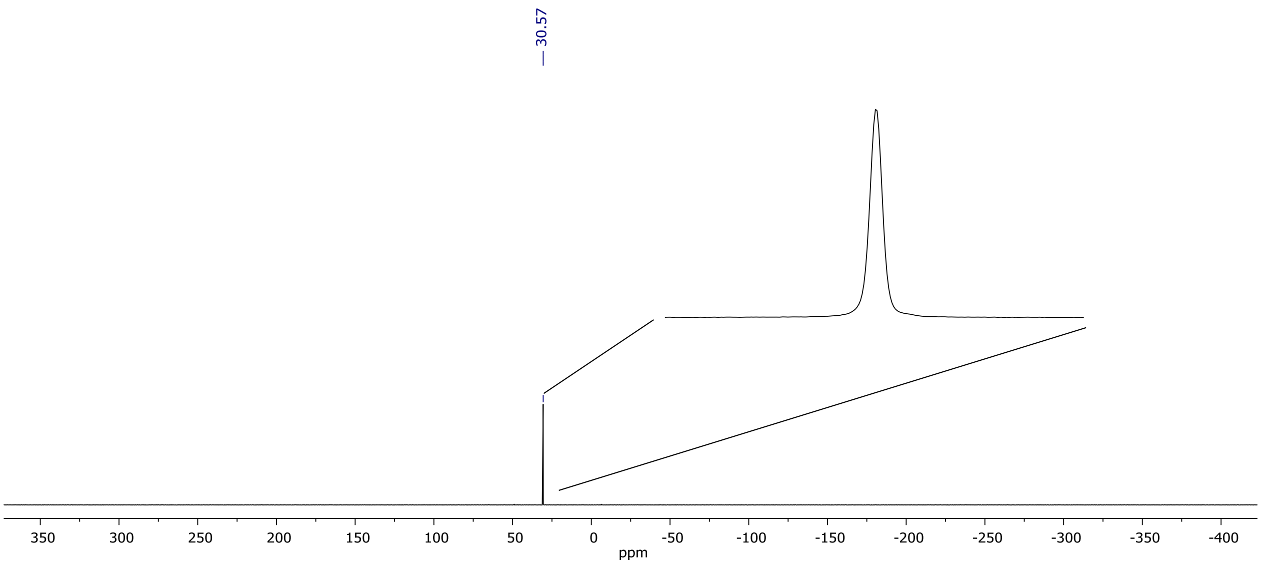

Figure S27: ^{31}P NMR spectrum (THF-d_8, 300 K, 162 MHz) of **4**.

Tris(tetramethylguanidinyl)phosphine–CO_2 adduct 5: A stirred solution of phosphine **1** (500 mg, 1.34 mmol) in diethyl ether (15 mL) was pressurized with 2 bar carbon dioxide for 15 minutes in a Schlenk flask as follows: A Schlenk flask containing the solution of **1** was frozen in liquid nitrogen and the argon atmosphere in the Schlenk flask was removed *in vacuo*. After warming the solution up to room temperature, the Schlenk flask was pressurized with 2 bar CO_2. After 15 minutes the pressure was released, and the precipitated white solid component was filtered off. The filtrate was evaporated to dryness. Compound **5** was isolated as white powder in quantitative yield. Single crystals were obtained by pressurizing a THF solution of **1** with 1 bar CO_2 for 3 hours (*vide infra*). Solid compound **5** can be stored under an atmosphere of dry argon for more than one month without decomposition. **5** is stable in dichloromethane and difluorobenzene, but only stable for >20 minutes in acetonitrile at temperatures below 0 °C (see NMR spectra in MeCN-d_3 below). **5** is insoluble in THF, benzene, toluene, *n*-hexane, and diethyl ether. In addition, an NMR reaction of an acetonitrile solution of phosphine **1** under 1 bar ^{13}C-labeled carbon dioxide pressure at 0 °C confirmed the immediate conversion of **1** in the phosphine–CO_2 adduct **3** in solution (see NMR spectra below).

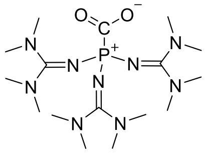

^{1}H NMR (400 MHz, 273 K, dichloromethane-d_2): δ = 2.86 (s, CH_3).

^{13}C{^{1}H} NMR (100.6 MHz, 273 K, dichloromethane-d_2): δ = 171.9) (d, $^1J_{PC}$ = 181.9 Hz, PCO_2), 162.1 (d, $^2J_{PC}$ = 2.4 Hz, N_2CN), 40.3 (s, CH_3).

^{31}P NMR (161.9 MHz, 273 K, dichloromethane-d_2): δ = –15.1 (s).

^{1}H NMR (400 MHz, 273 K, MeCN-d_3): δ = 2.82 (s, CH_3).

^{13}C{^{1}H} NMR (100.6 MHz, 273 K, MeCN-d_3): δ = 171.3 (d, $^1J_{PC}$ = 181.9 Hz, PCO_2), 162.3 (d, $^2J_{PC}$ = 2.4 Hz, N_2CN), 40.2 (s, CH_3).

^{31}P NMR (161.9 MHz, 273 K, MeCN-d_3): δ = –14.2 (s).

^{31}P NMR (161.9 MHz, 273 K, difluorobenzene, C_6D_6 lock): δ = –13.7 (s).

HRMS (ESI): m/z calculated for $[C_{15}H_{37}N_9P]^+$ $(M–CO_2+H)^+$ 374.29041, found 374.29050.

CHN analysis: calcd. for $C_{16}H_{36}N_9PO_2$: C, 46.03; H, 8.69; N, 30.19. Found: C, 45.84; H, 8.74; N, 29.51.

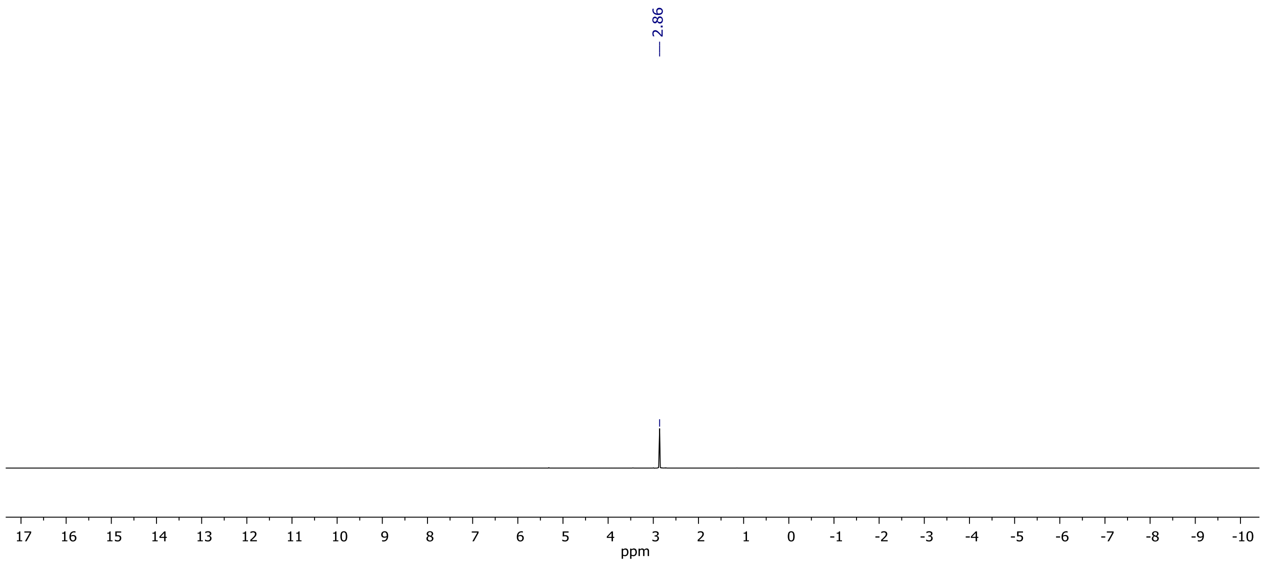

Figure S28: ^{1}H NMR spectrum (dichloromethane-d_2, 273 K, 400 MHz) of **5**.

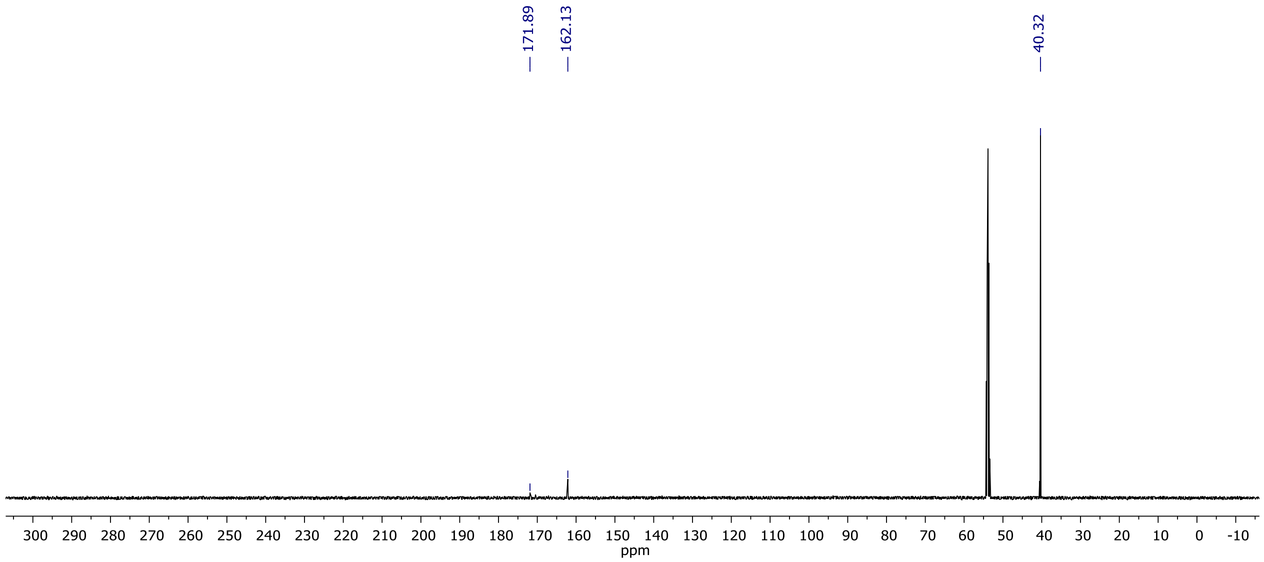

Figure S29: $^{13}C\{^{1}H\}$ NMR spectrum (dichloromethane-d_2, 273 K, 101 MHz) of **5**.

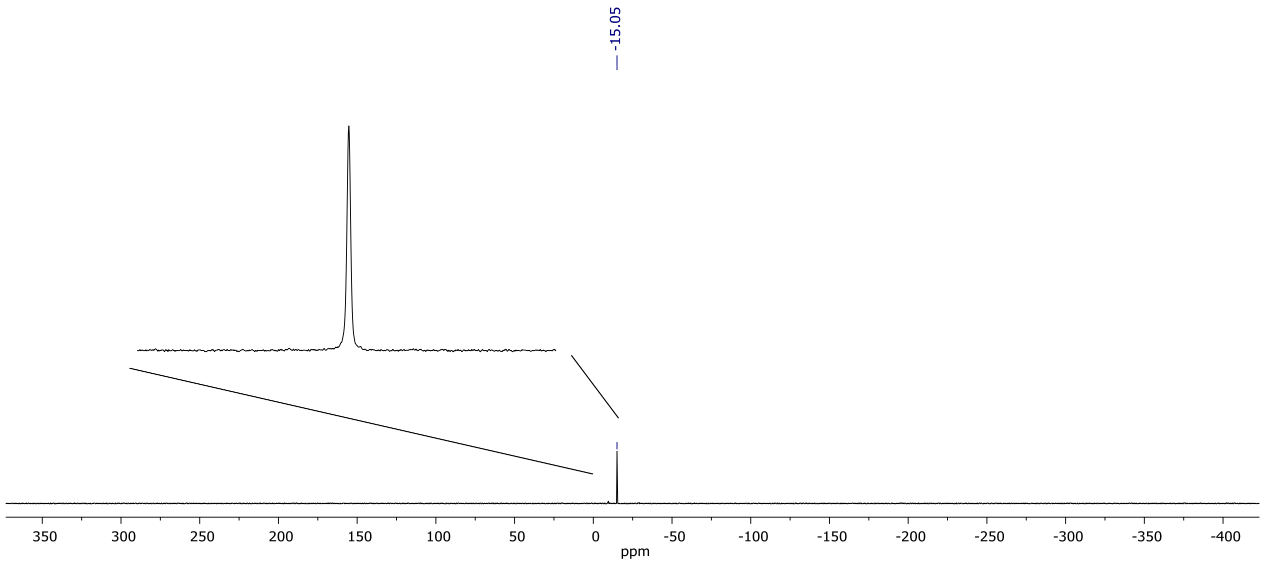

Figure S30: ^{31}P NMR spectrum (dichloromethane-d_2, 273 K, 162 MHz) of **5**.

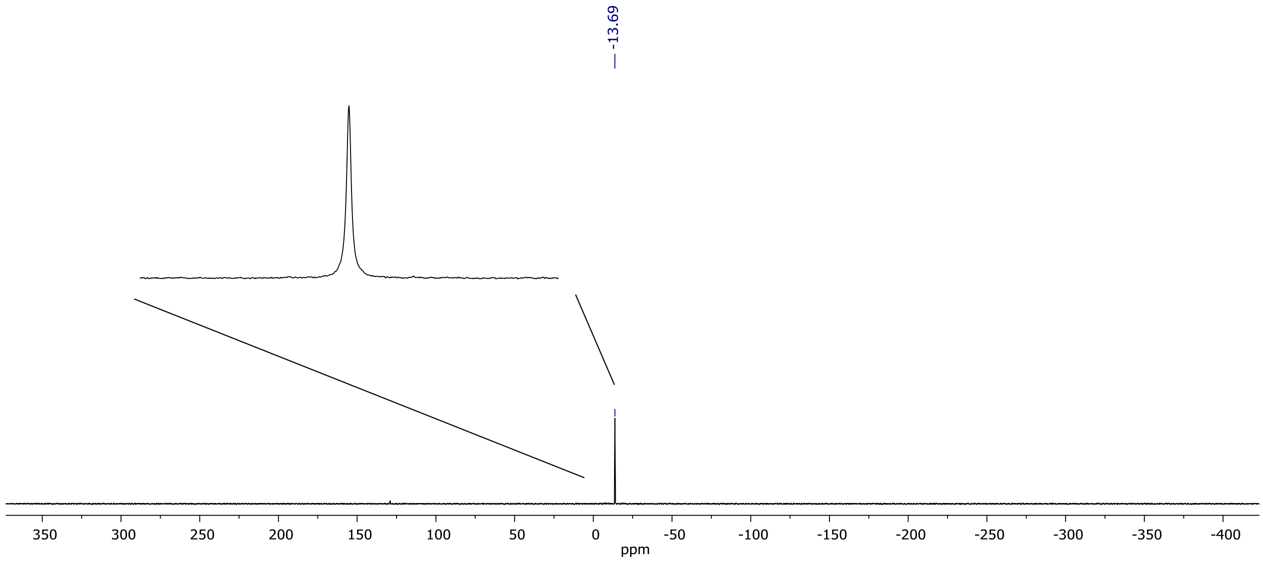

Figure S31: ^{31}P NMR spectrum (difluorobenzene, 273 K, 162 MHz) of **5**.

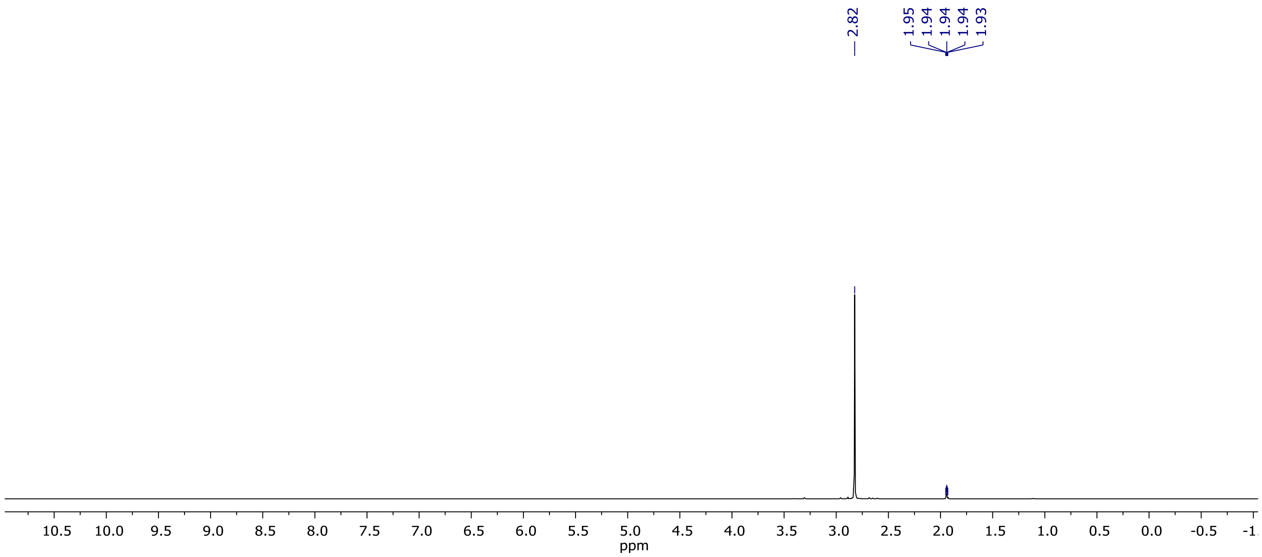

Figure S32: ^{1}H NMR spectrum (MeCN-d_3, 273 K, 400 MHz) of **5**.

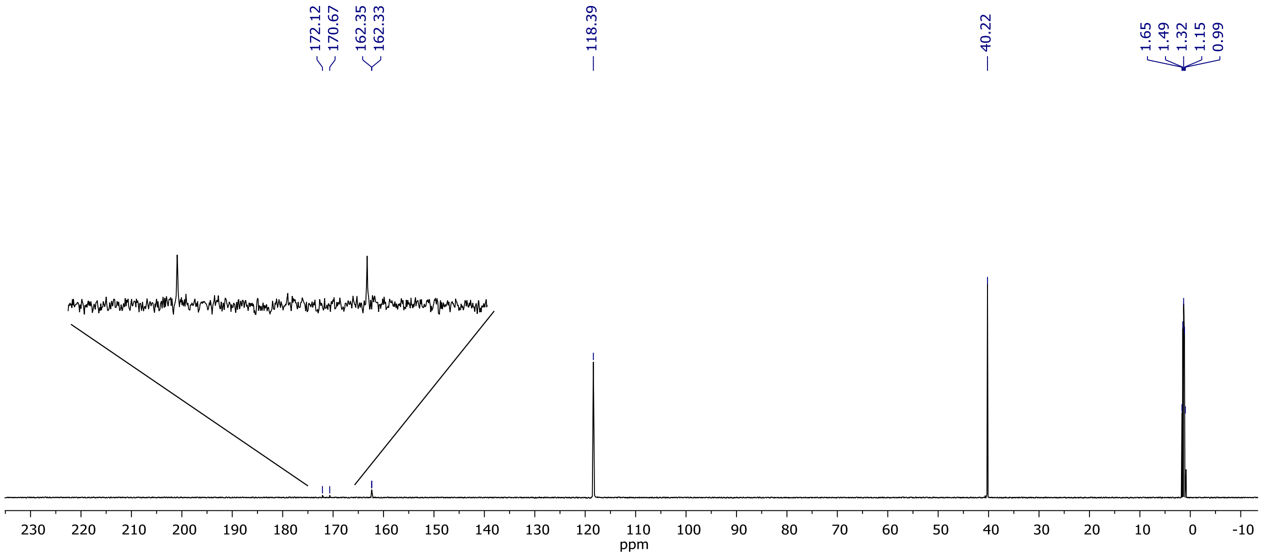

Figure S33: ^{13}C{^{1}H} NMR spectrum (MeCN-d_3, 273 K, 101 MHz) of **5**.

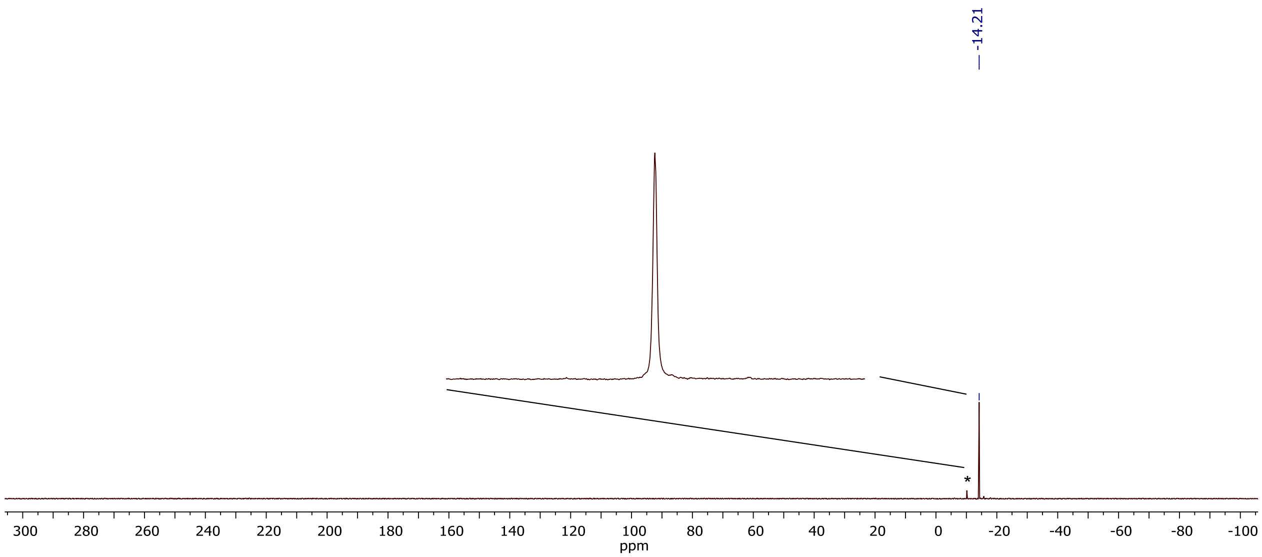

Figure S34: ^{31}P NMR spectrum (MeCN-d_3, 273 K, 162 MHz) of **5**. *decomposition product

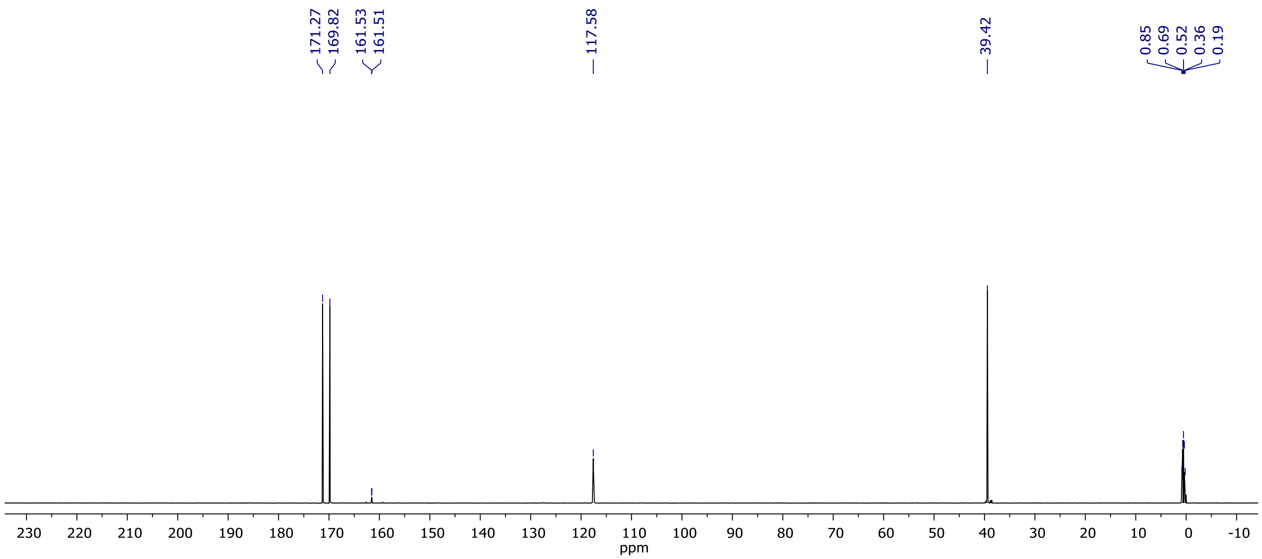

Figure S35: $^{13}C\{^1H\}$ NMR spectrum (MeCN-d_3, 273 K, 101 MHz) of (tmg)P-$^{13}CO_2$ at 0 °C.

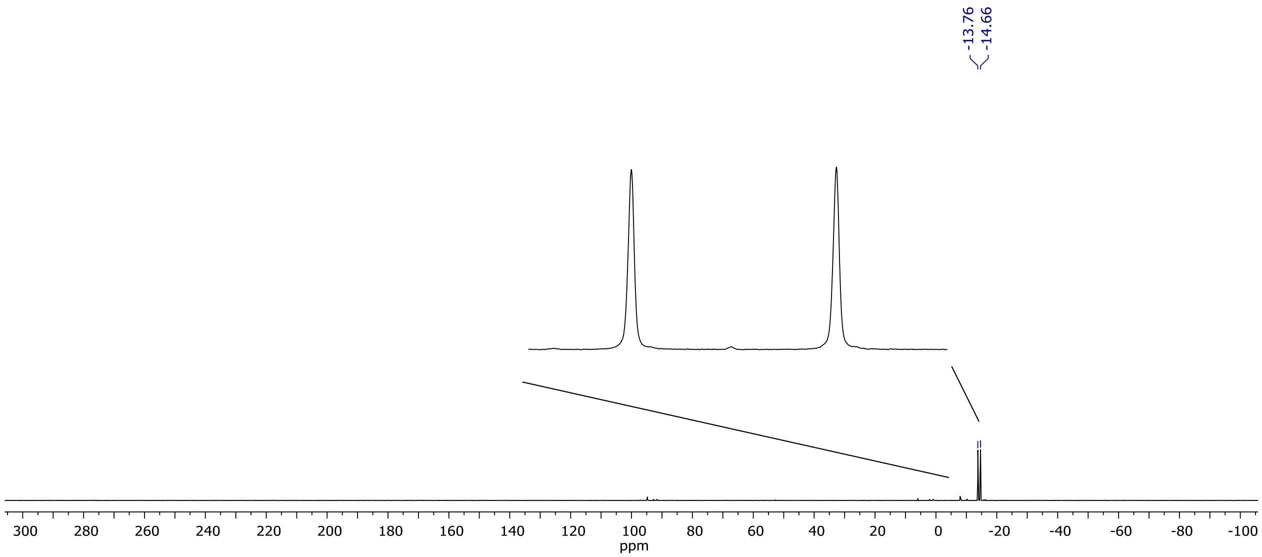

Figure S36: ^{31}P NMR spectrum (MeCN-d_3, 273 K, 162 MHz) of (tmg)P-$^{13}CO_2$ at 0 °C.

Tris(tetramethylguanidinyl)phosphine–SO_2 adduct 6: Phosphine **1** (500 mg, 1.34 mmol, 2 eq.) and DABSO (161 mg, 0.67 mmol, 1 eq.) were suspended in THF (15 mL) at room temperature. The mixture was stirred for 40 minutes and the formed white precipitate was filtered off. To remove DABCO the precipitate was washed with *n*-hexane (4 x 5 mL). After evaporation to dryness *in vacuo*, **6** was isolated as a white solid in 95% yield (556 mg, 1.27 mmol). The phosphine–SO_2 adduct **6** is insoluble in toluene, benzene, and *n*-hexane and soluble in acetonitrile and dichloromethane, but decomposes within 3 hours.

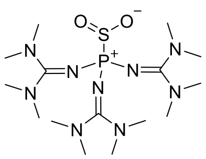

^{1}H NMR (400 MHz, 300 K, CD_2Cl_2): δ = 2.86 (s, CH_3).

^{13}C{^{1}H} NMR (100.6 MHz, 300 K, CD_2Cl_2): δ = 162.1 (d, N_2CN), 40.5 (s, CH_3).

^{31}P NMR (161.9 MHz, 300 K, CD_2Cl_2): δ = –10.4 (s).

HRMS (ESI): m/z calculated for $[C_{15}H_{37}N_9OP]^+$ $(M–SO_2+OH)^+$ 390.28532, found 390.28541.

CHN analysis: calcd. for $C_{15}H_{36}N_9PSO_2$: C, 41.18; H, 8.29; N, 28.81. Found: C, 40.82; H, 8.35; N, 28.36.

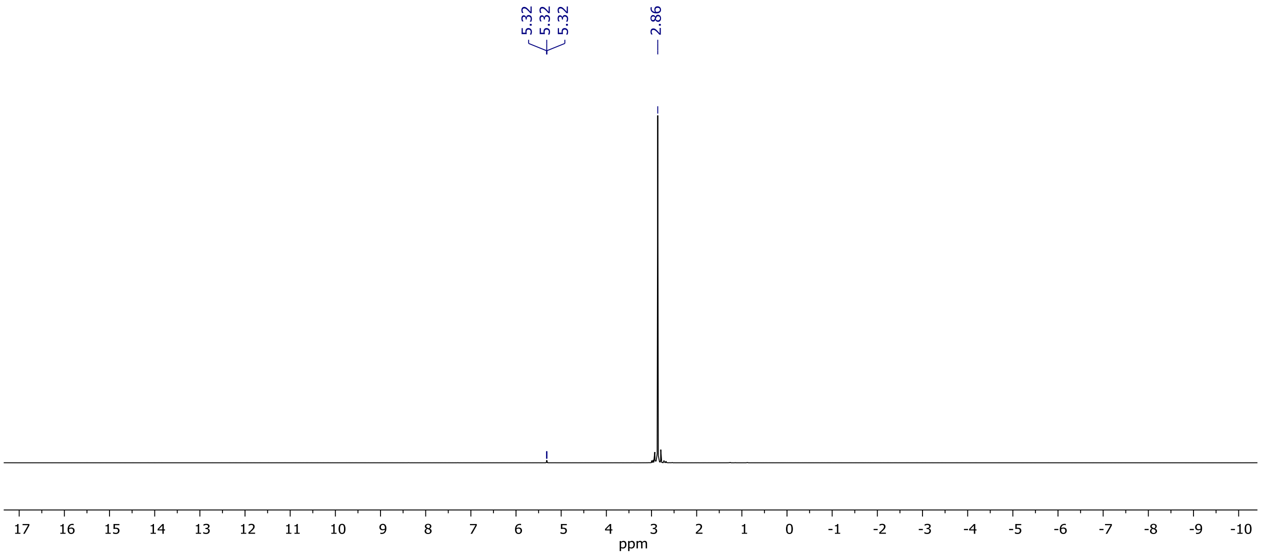

Figure S37: ^{1}H NMR spectrum (CD_2Cl_2, 300 K, 400 MHz) of **6**.

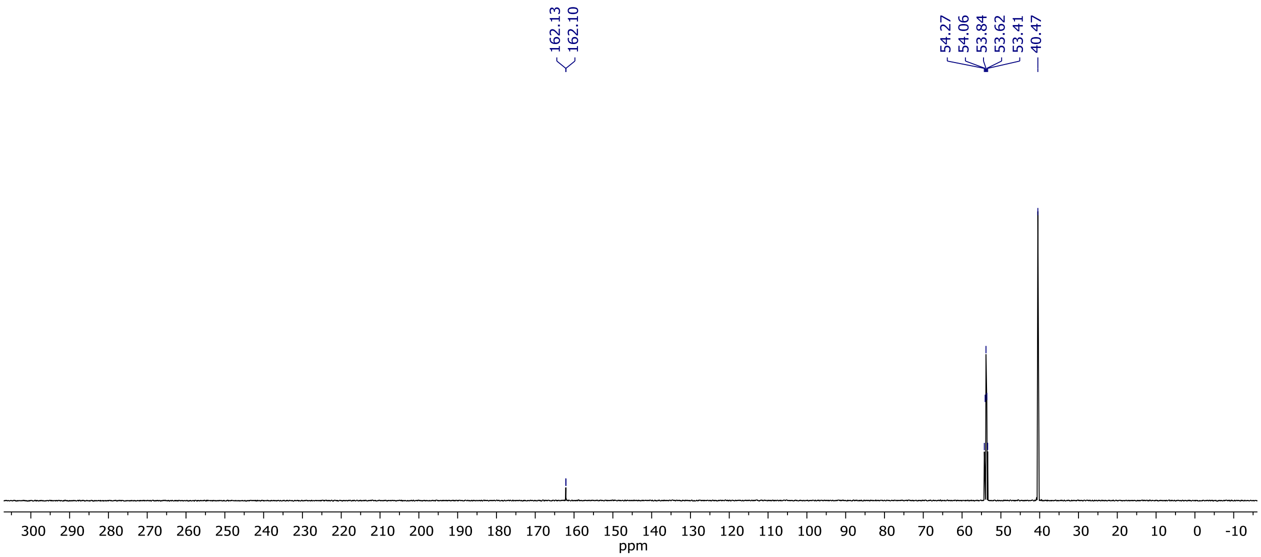

Figure S38: $^{13}C\{^{1}H\}$ NMR spectrum (CD_2Cl_2, 300 K, 101 MHz) of **6**.

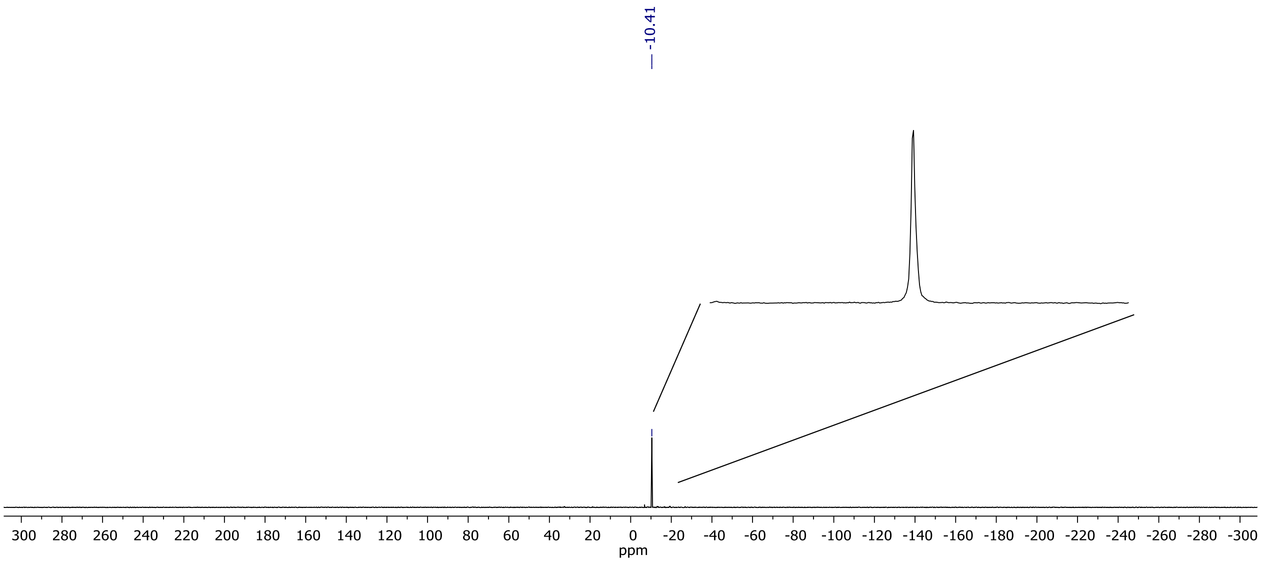

Figure S39: ^{31}P NMR spectrum (CD_2Cl_2, 300 K, 162 MHz) of **6**.

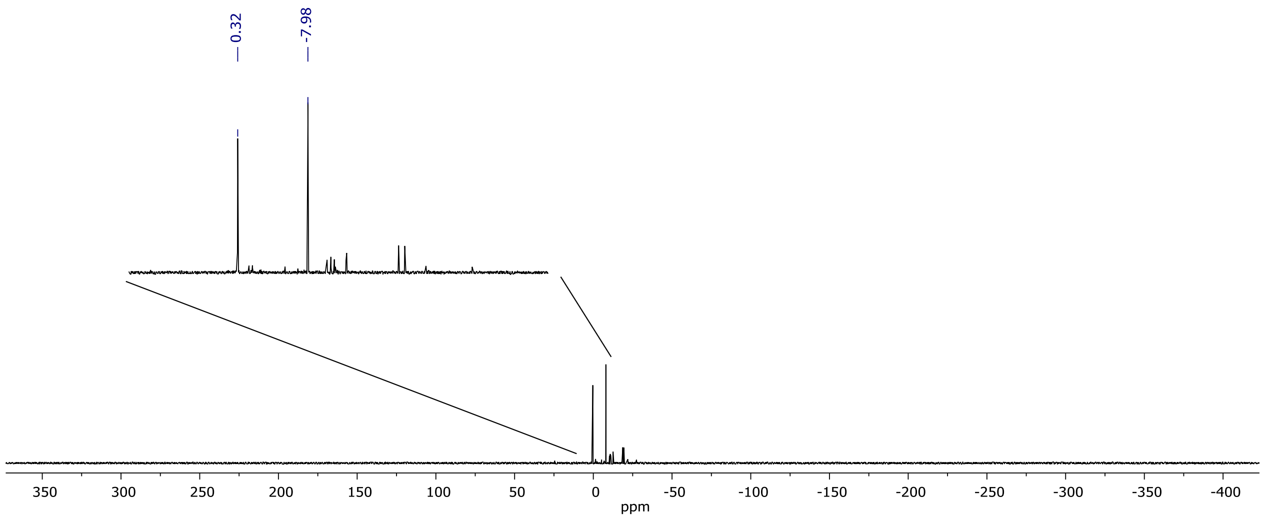

Figure S40: ^{31}P NMR spectrum (CD_2Cl_2, 300 K, 162 MHz) of **6** after storing for 3 hours at room temperature.

Tris(tetramethylguanidinyl)phosphine oxide 7: In a Schlenk flask a solution of phosphine **1** (500 mg, 1.34 mmol) in THF was pressurized with 2 bar N_2O as follows: A Schlenk flask containing the solution of **1** was frozen in liquid nitrogen and the argon atmosphere in the Schlenk flask was removed *in vacuo*. After warming the solution up to room temperature, the Schlenk flask was pressurized with 2 bar N_2O. After 10 minutes the pressure was released, and the volatiles were removed *in vacuo*. Compound **7** was isolated as a white solid in quantitative yield. Single crystals as colorless plates were obtained by vapor diffusion of *n*-hexane into a THF solution of **7** (*vide infra*).

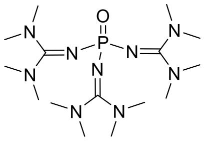

^{1}H NMR (400 MHz, 300 K, THF-d_8): δ = 2.79 (s, CH_3).

^{13}C{^{1}H} NMR (100.6 MHz, 300 K, THF-d_8): δ = 159.1 (d, N_2CN), 40.2 (s, CH_3).

^{31}P NMR (161.9 MHz, 300 K, THF-d_8): δ = –6.5 (s).

^{31}P{^{1}H} NMR (161.9 MHz, 300 K, THF-d_8): δ = –6.5 (s).

HRMS (ESI): m/z calculated for $[C_{15}H_{37}N_9OP]^+$ $(M+H)^+$ 390.28532, found 390.28547.

CHN analysis: calcd. for $C_{15}H_{36}N_9PO$: C, 46.26; H, 9.32; N, 32.37. Found: C, 46.21; H, 9.32; N, 32.21.

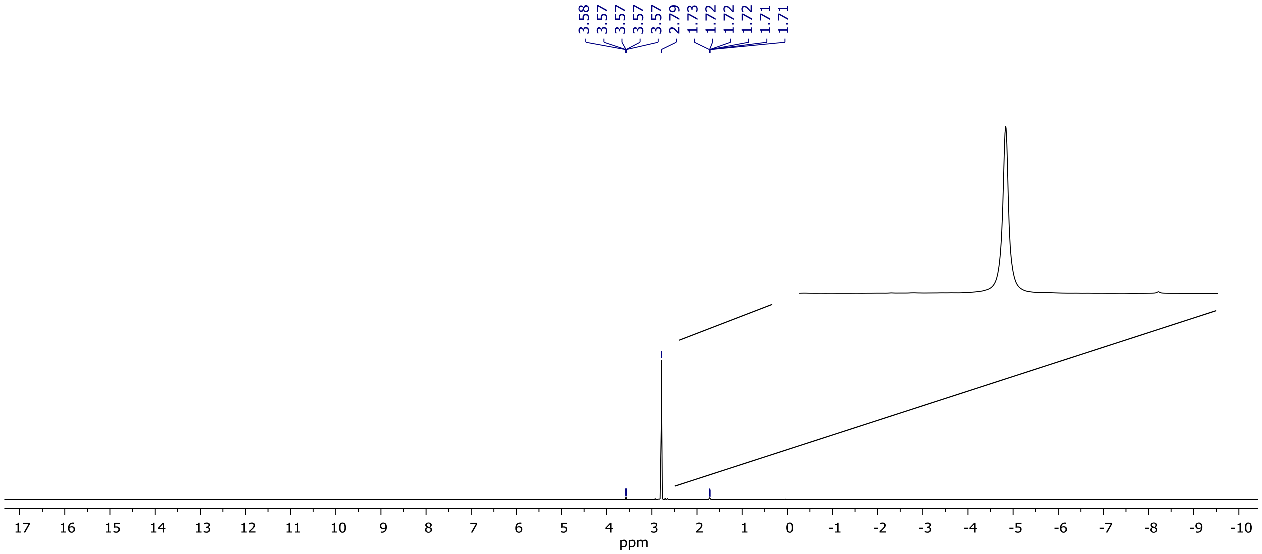

Figure S41: ^{1}H NMR spectrum (THF-d_8, 300 K, 400 MHz) of **7**.

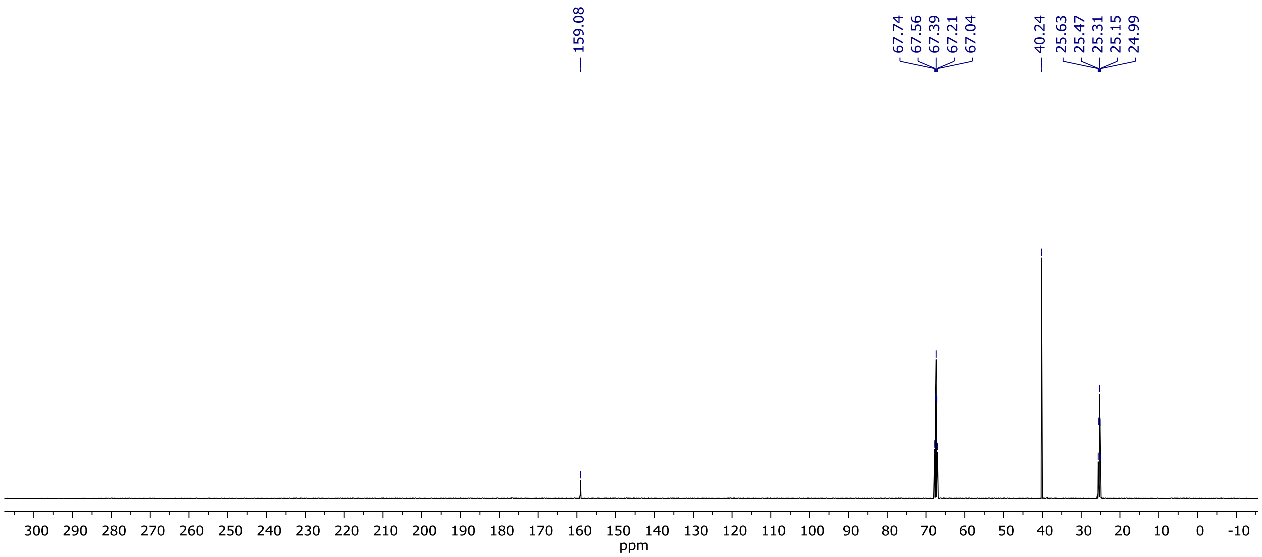

Figure S42: $^{13}C\{^{1}H\}$ NMR spectrum (THF-d_8, 300 K, 101 MHz) of **7**.

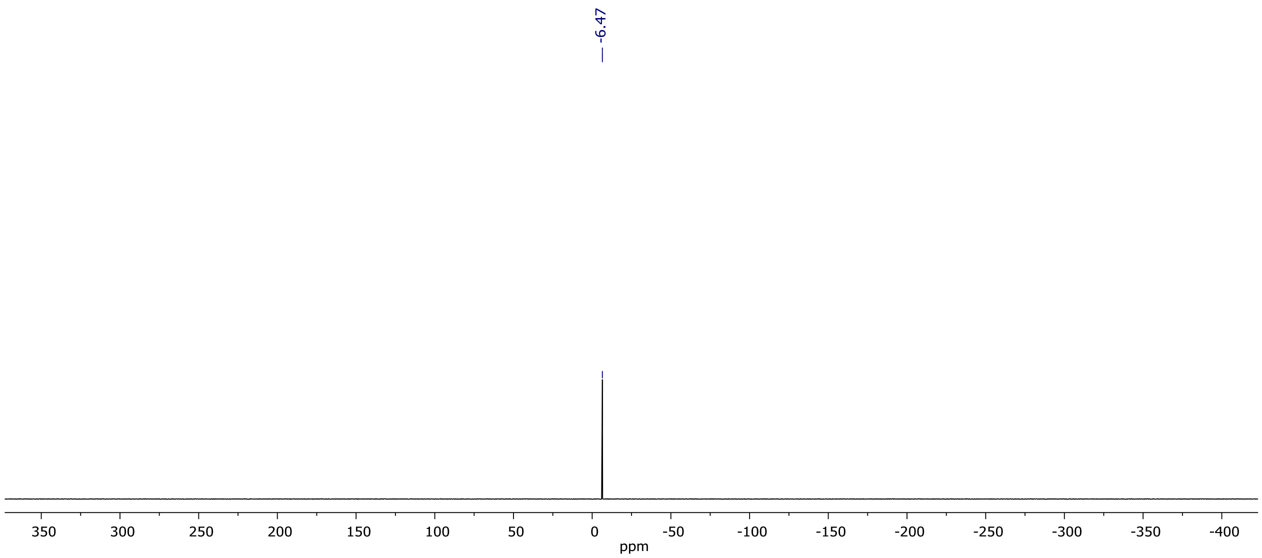

Figure S43: ^{31}P NMR spectrum (THF-d_8, 300 K, 162 MHz) of **7**.

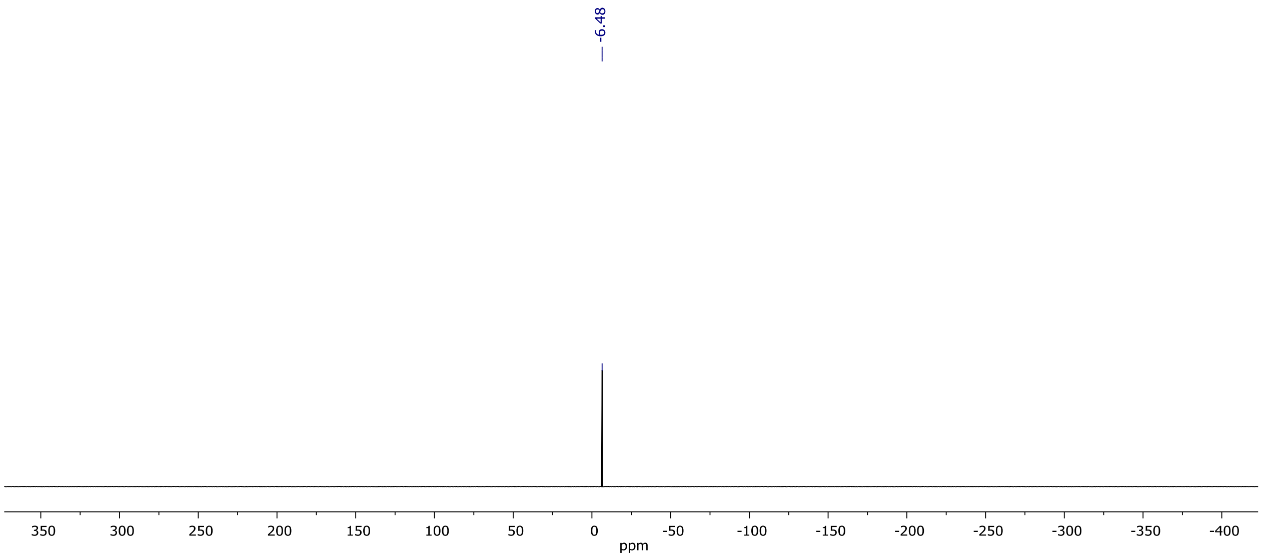

Figure S44: $^{31}P\{^{1}H\}$ NMR spectrum (THF-d_8, 300 K, 162 MHz) of **7**.

Tris(tetramethylguanidinyl)phosphine–Rh complex 8:

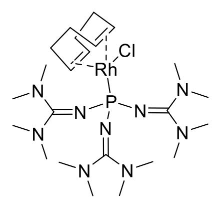

1 (209 mg, 0.56 mmol, 2 eq.) and [{Rh(cod)Cl}$_2$] (138 mg, 0.28 mmol, 1 eq.) were dissolved in THF (5 mL) and stirred for 1 h at room temperature. All volatile compounds were removed *in vacuo* and complex **8** was obtained as an orange solid in quantitative yield. Single crystals as yellow plates were obtained by diffusion of *n*-hexane into a THF solution of **8** at –40 °C (*vide infra*).

^{1}H NMR (400 MHz, 300 K, C_6D_6): δ = 4.95 (m, 2H, cod), 3.81 (m, 2H, cod), 2.87 (s, 36H, CH_3), 2.29 (m, 4H, cod), 2.17 (m, 4H, cod).

^{13}C{^{1}H} NMR (100.6 MHz, 300 K, C_6D_6): δ = 158.9 (d, N_2CN), 99.1 (cod), 69.2 (cod), 40.7 (s, CH_3), 33.9 (cod), 29.4 (cod).

^{31}P NMR (161.9 MHz, 300 K, C_6D_6): δ = 49.9 (d, $^1J_{PRh}$ = 179.5 Hz).

^{31}P{^{1}H} NMR (161.9 MHz, 300 K, C_6D_6): δ = 49.9 (d, $^1J_{PRh}$ = 179.5 Hz).

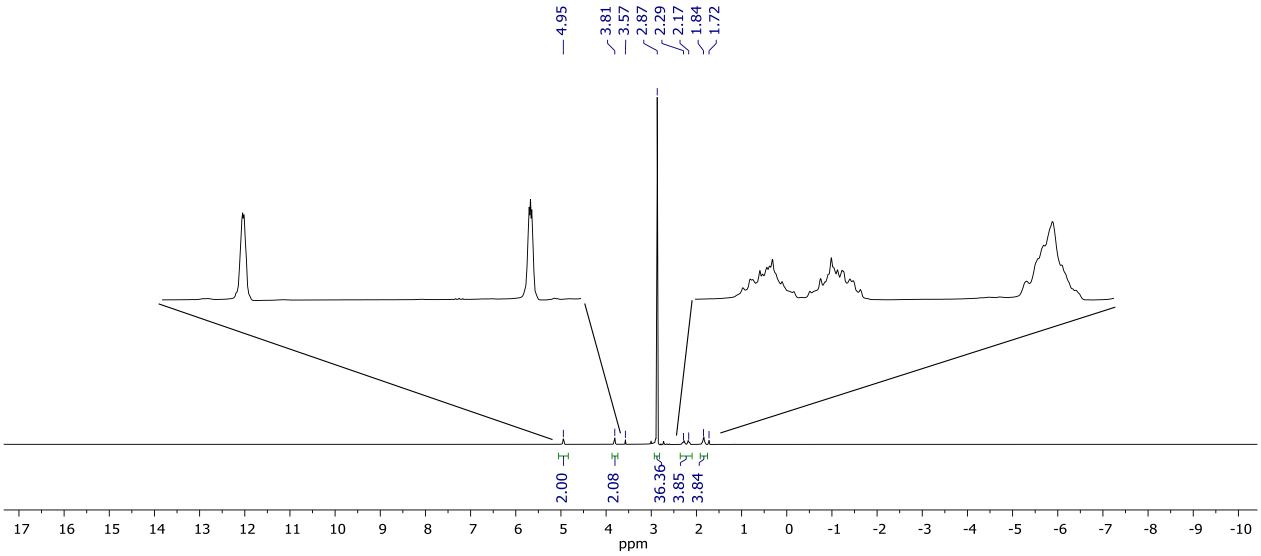

Figure S45: ^{1}H NMR spectrum (THF-d_8, 300 K, 400 MHz) of **8**.

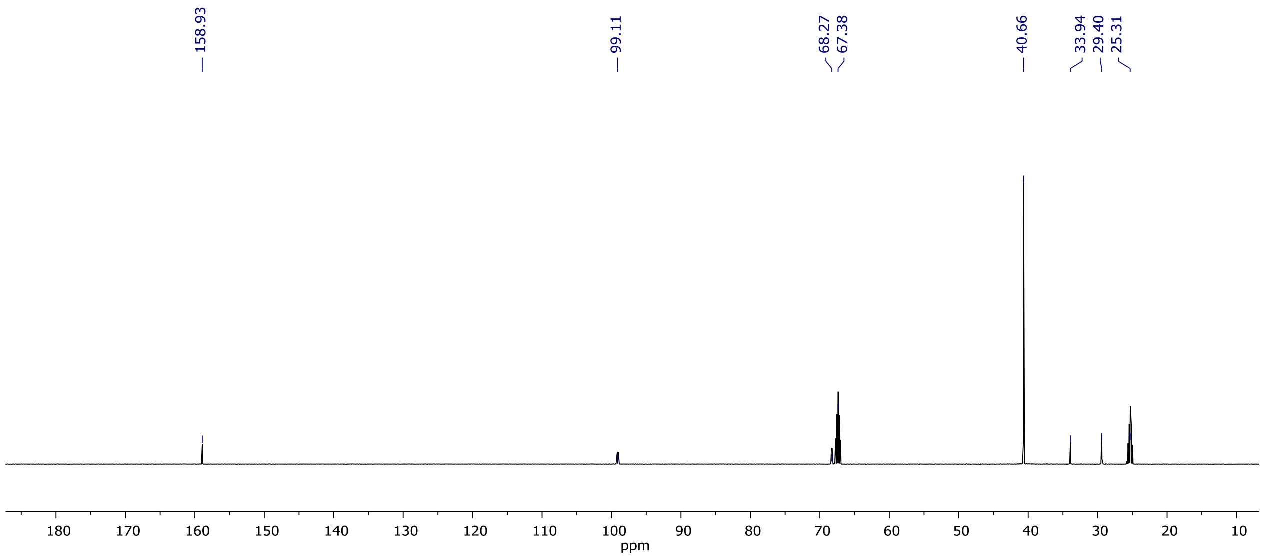

Figure S46: $^{13}C\{^{1}H\}$ NMR spectrum (THF-d_8, 300 K, 101 MHz) of **8**.

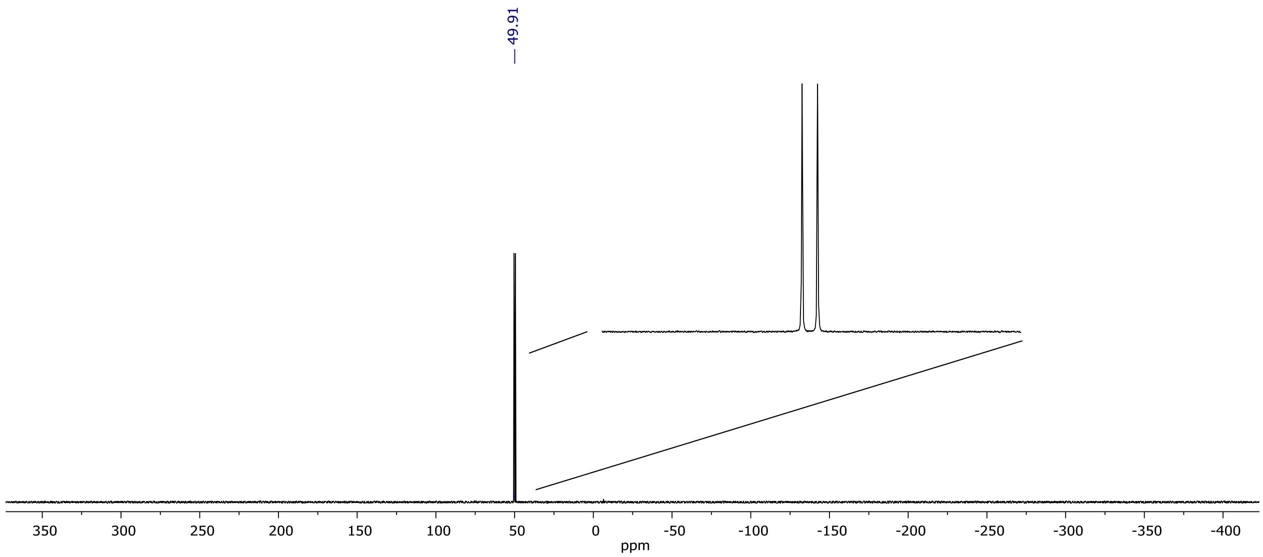

Figure S47: ^{31}P NMR spectrum (THF-d_8, 300 K, 162 MHz) of **8**.

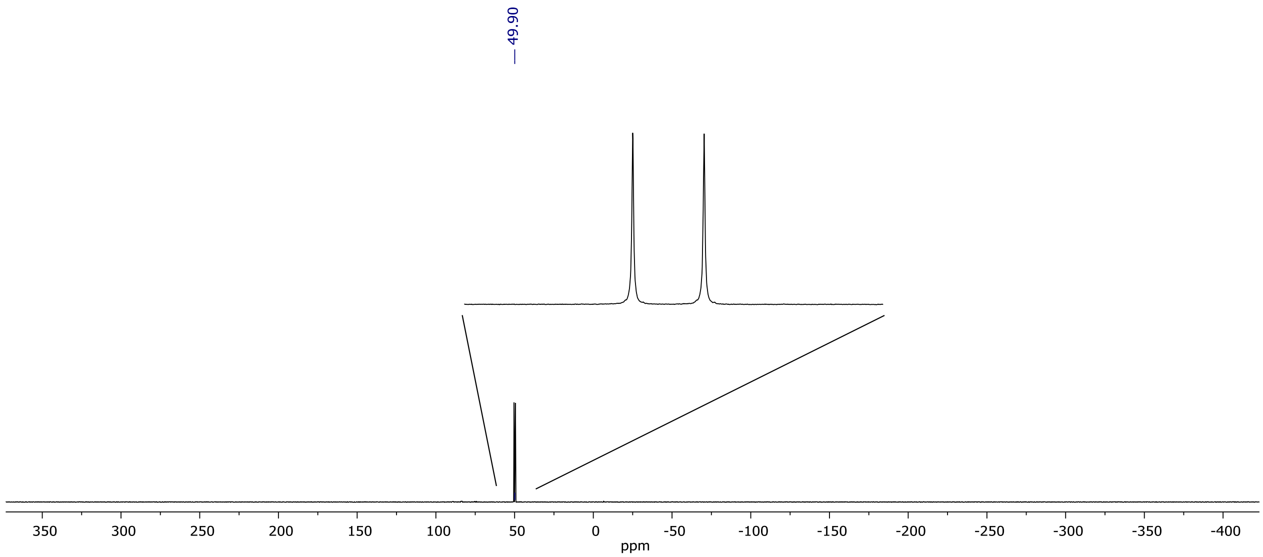

Figure S48: $^{31}P\{^{1}H\}$ NMR spectrum (THF-d_8, 300 K, 162 MHz) of **8**.

Tris(tetramethylguanidinyl)phosphine–Au complex 9:

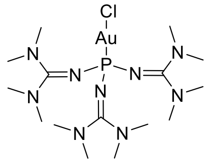

1 (145 mg, 0.39 mmol) and Au(tht)Cl (125 mg, 0.39 mmol) were dissolved in THF (4 mL) and stirred for 1 h at room temperature. All volatile compounds were removed *in vacuo* and complex **9** was obtained as an off-white solid in quantitative yield. Single crystals as colorless plates were obtained by diffusion of *n*-hexane into a THF solution of **9** (*vide infra*).

^{1}H NMR (400 MHz, 300 K, C_6D_6): δ = 2.68 (s, CH_3).

^{13}C{^{1}H} NMR (100.6 MHz, 300 K, C_6D_6): δ = 160.7 (d, N_2CN), 40.3 (s, CH_3).

^{31}P NMR (161.9 MHz, 300 K, C_6D_6): δ = 48.8 (s, br).

^{31}P{^{1}H} NMR (161.9 MHz, 300 K, C_6D_6): δ = 48.8 (s).

HRMS (ESI): m/z calculated for $[C_{15}H_{37}N_9PAuCl]^+$ $(M+H)^+$ 606.22636, found 606.22643.

CHN analysis: calcd. for $C_{15}H_{36}N_9P$: C, 29.69; H, 6.15; N, 20.77. Found: C, 29.77; H, 6.06; N, 20.60.

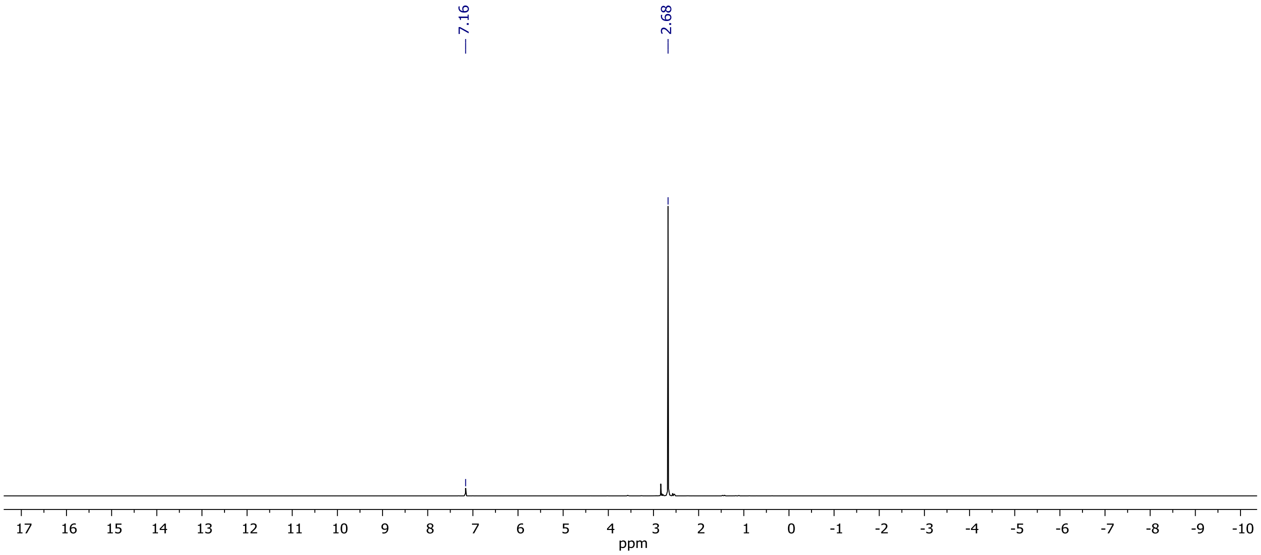

Figure S49: ^{1}H NMR spectrum (C_6D_6, 300 K, 400 MHz) of **9**.

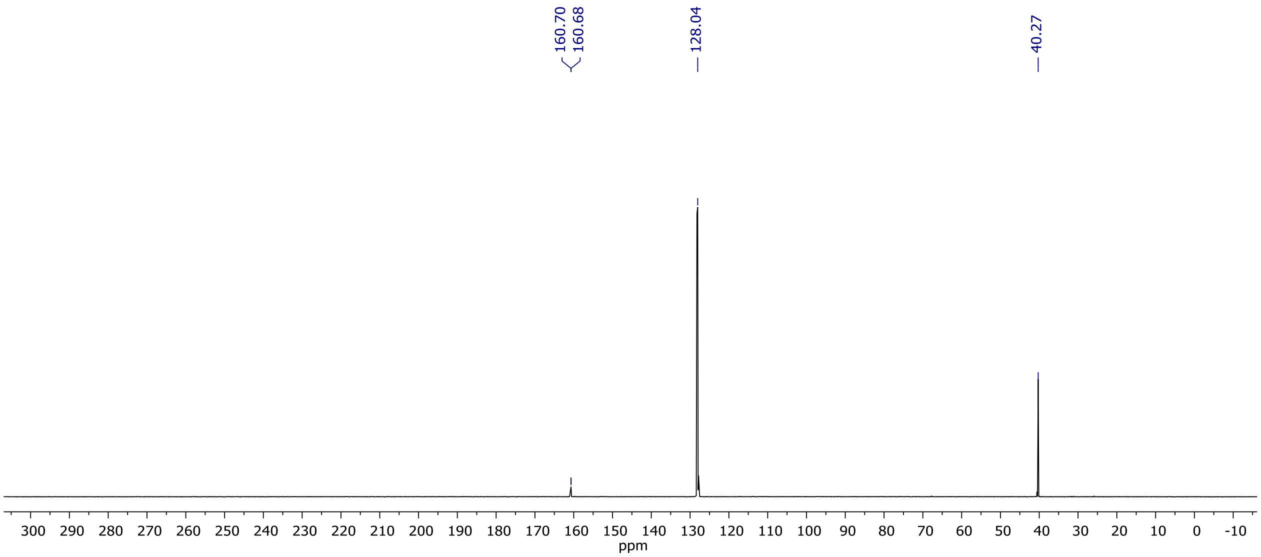

Figure S50: $^{13}C\{^{1}H\}$ NMR spectrum (C_6D_6, 300 K, 101 MHz) of **9**.

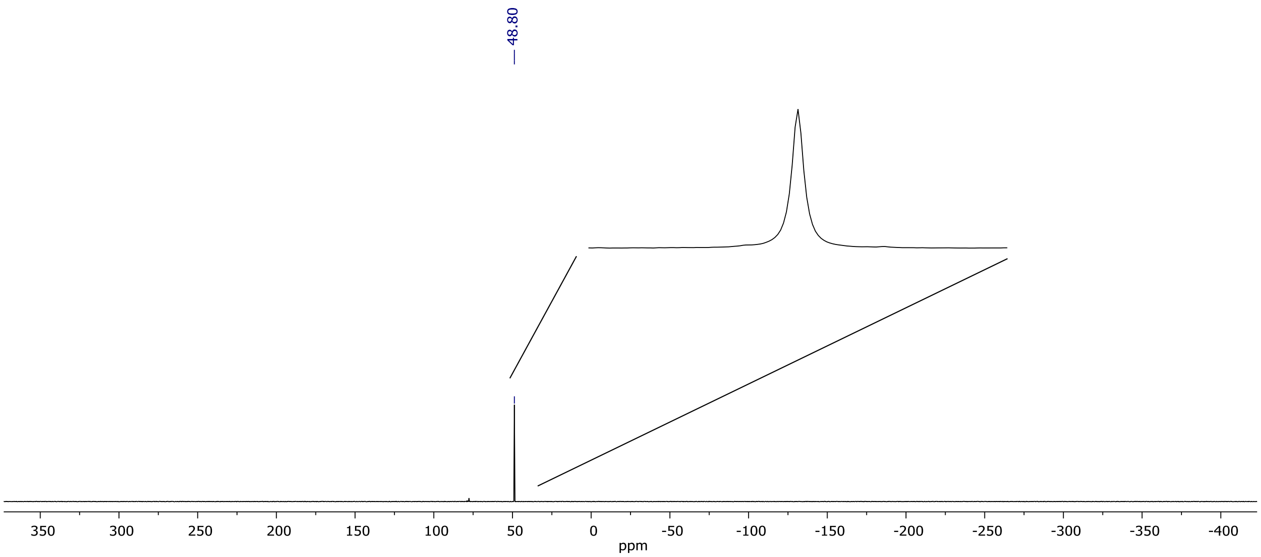

Figure S51: $^{31}P\{^{1}H\}$NMR spectrum (C_6D_6, 300 K, 162 MHz) of **9**.

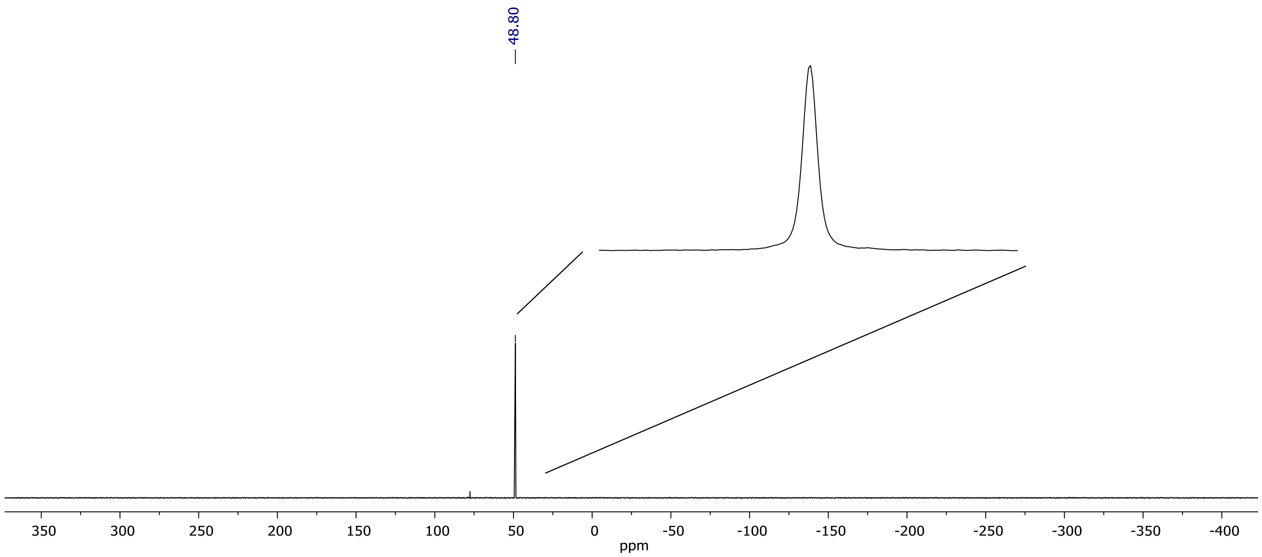

Figure S52: ^{31}P NMR spectrum (C_6D_6, 300 K, 162 MHz) of **9**.

Tris(tetramethylguanidinyl)phosphine–Pd complex 10:

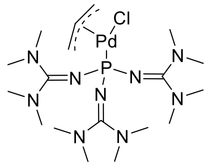

1 (209 mg, 0.56 mmol, 2 eq.) and [{Pd(allyl)Cl}$_2$] (102 mg, 0.28 mmol, 1 eq.) were dissolved in THF (5 mL) and stirred for 1 h at room temperature. All volatile compounds were removed *in vacuo* and complex **8** was obtained as a white solid in quantitative yield.

^{1}H NMR (400 MHz, 300 K, C_6D_6): δ = 5.28 (m, 1H, allyl-H), 4.37 (dd, $^3J_{HH}$ = 7.7 Hz, $^4J_{PH}$ = 7.8 Hz, 1 H, allyl-H), 3.53 (m, 1 H, allyl-H), 2.99 (br, 1 H, allyl-H), 2.78 (s, 36 H, CH_3), 2.63 (br, 1 H, allyl-H).

^{31}P NMR (161.9 MHz, 300 K, C_6D_6): δ = 60.0 (m).

HRMS (ESI): m/z calculated for $[C_{18}H_{43}N_9PClPd]^+$ $(M+H)^+$ 557.20969, found 557.20946.

CHN analysis: calcd. for $C_{18}H_{42}N_9PClPd$: C, 38.78; H, 7.59; N, 22.61. Found: C, 38.90; H, 7.35; N, 21.58.

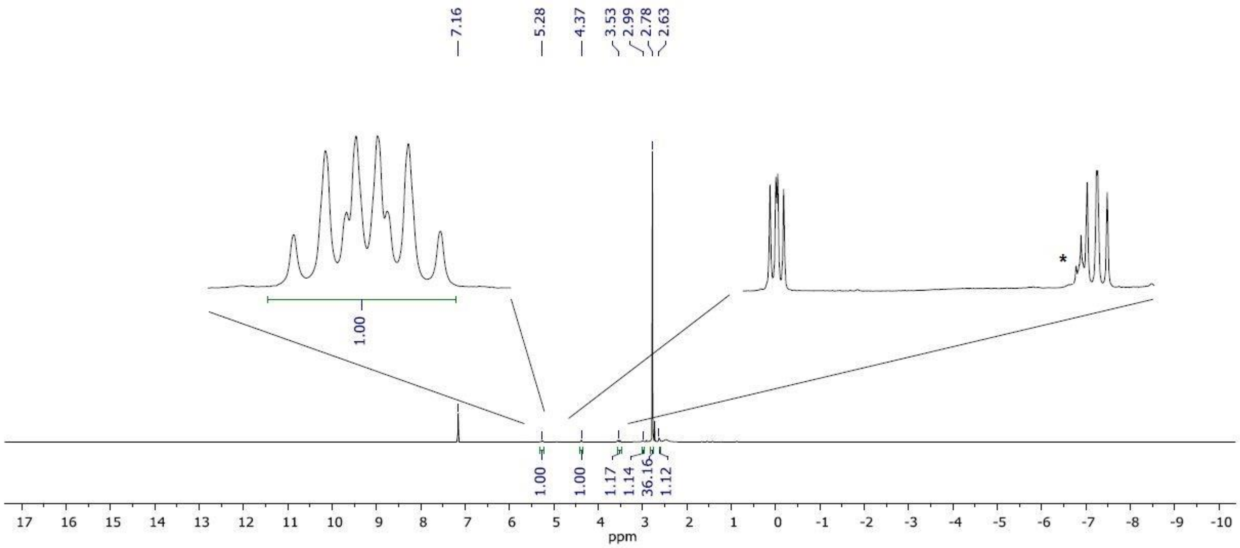

Figure S53: ^{1}H NMR spectrum (C_6D_6, 300 K, 400 MHz) of **8.** *THF

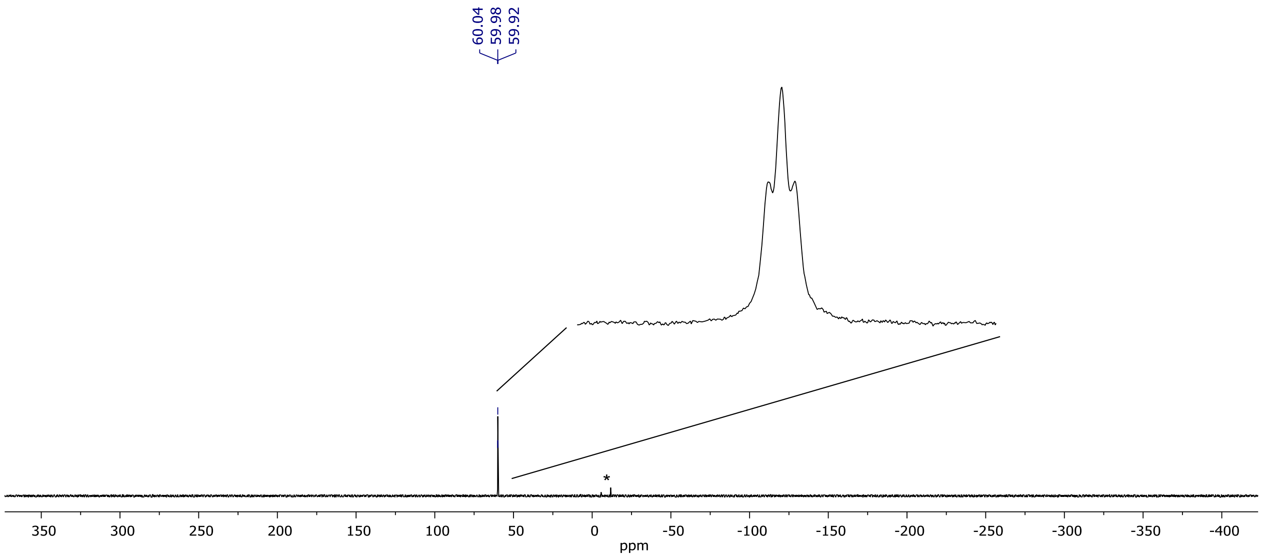

Figure S54: ^{31}P NMR spectrum (C_6D_6, 300 K, 162 MHz) of **8**. *impurity

Determination of $pK_{a(MeCN)}$ of 1 using phosphonium salt [(pyrr)$_3$PCH$_2$Ph][OTf]

For the experimental determination of the pK_{BH+} values a method by Morris et al. was carried out.[5] A solution of **1** (0.25 mL, 0.16 M standard solution in acetonitrile) and [(pyrr)$_3$PCH$_2$Ph][OTf] (0.25 mL, 0.16 M standard solution in acetonitrile) was prepared. Instantly, an ^{31}P{gated ^{1}H} NMR spectrum (T_1 time = 25 s) was recorded. The following species appear in the NMR spectrum: **1** (s, 93.2 ppm), P$_2$(tmg)$_5$OTF (92.4 ppm, d, $^{1}J_{PP}$ = 226 Hz and 1.02 ppm, d, $^{1}J_{PP}$ = 226 Hz), (pyrr)$_3$PCHPh (40.7 ppm, s), [(pyrr)$_3$PCH$_2$Ph][OTf] (40.1 ppm) and **1**·HOTf (s, –16.7 ppm).

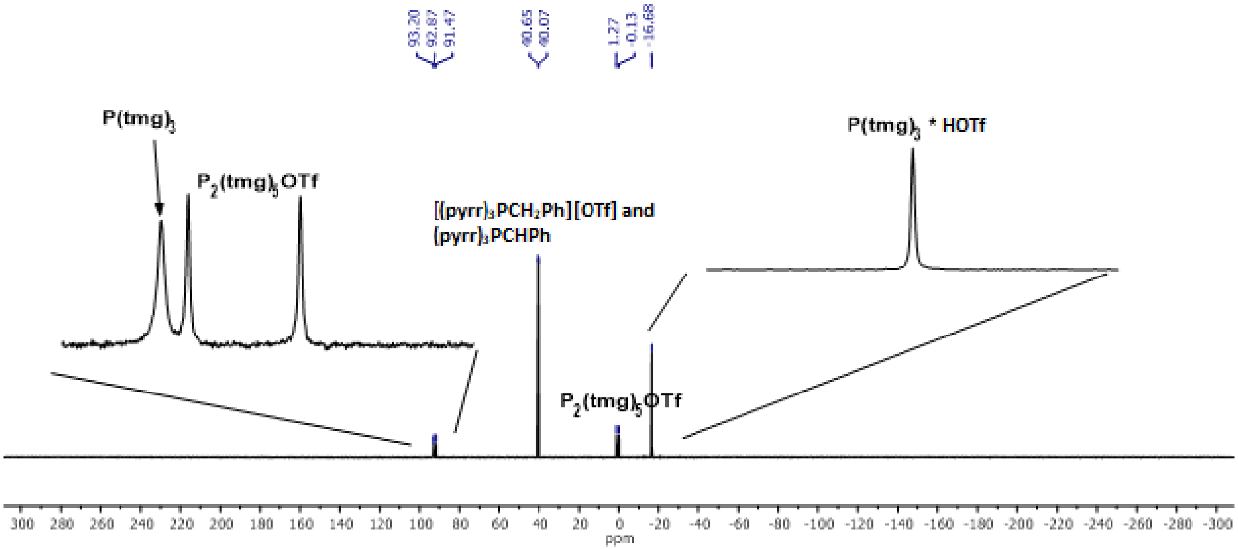

Figure S55: ^{31}P {gated ^{1}H} NMR spectrum (acetonitrile-d_3, T_1 time = 25 s, 300 K, 162 MHz) of the reaction mixture.

Generally, for an equilibrium:

$$\mathbf{1} + [(pyrr)_3PCH_2Ph][OTf] \leftrightharpoons \mathbf{1}\cdot HOTf + (pyrr)_3PCHPh$$

the equilibrium constant K and subsequently the pK_{BH+} value can be determined.

In our experiment, two equilibria are present in the reaction mixture:

$$\mathbf{1} + [(pyrr)_3PCH_2Ph][OTf] \leftrightharpoons \mathbf{1}\cdot HOTf + (pyrr)_3PCHPh \qquad (1)$$

$$\mathbf{1} + \mathbf{1}\cdot HOTf \leftrightharpoons [P_2(tmg)_5][OTf] + (tmg)H \qquad (2)$$

The equilibrium constant K_{12} of the total reaction can be determined to be K_{12} = 1.64 considering the ratio of [(pyrr)$_3$PCHPh]/[(pyrr)$_3$PCH$_2$PhOTf] = 1.12 and [P$_2$(tmg)$_5$][OTf]/[**1**] = 1.21. The particular equilibrium constant K_2 from the second equilibrium can be determined from the independent formation of P$_2$(tmg)$_5$Cl by the reaction of phosphine **1** and phosphonium salt **1**·HCl in acetonitrile (Figure S52). Using the ratio [P$_2$(tmg)$_5$][Cl]/[**1**] = 1.09 and [P$_2$(tmg)$_5$][Cl]/[**1**·HOTf] = 0.79 to determine K_2 = 0.86 under the assumption that the counterions do not affect the equilibrium. The particular equilibrium constant K_1 for the first equilibrium which is needed to determine the pK_{BH+} value can therefore be calculated from K_{12} and K_2 to be K_2 = 1.09. The pK_{BH+} (MeCN) value can be calculated according to the following formula[5]:

$$pK_{BH+}(\mathbf{1}) = pK_{BH+}((pyrr)_3PCHPh) - pK + \Delta pK_d$$

Due to the approximately equal distance of the ion pairs of the phosphorus cations the following applies $\Delta pK_d \approx 0$. Using the $pK_{BH+}((pyrr)_3PCHPh) = 32.5$ in MeCN[6] the basicity of **1** in MeCN can be determined to be $pK_{BH+}(\mathbf{1}) = 32.7$.

The independent formation of $P_2(tmg)_5Cl$ by the reaction of phosphine **1** (15 mg, 0.04 mmol, 1 eq.) and phosphonium salt **1**·HCl (16.5 mg, 0.04 mmol, 1 eq.) in acetonitrile-d_3 in an ^{31}P NMR experiment was performed (Figure S52) and an $^{31}P\{gated\ ^1H\}$ NMR spectrum (T_1 time = 25 s) was recorded.

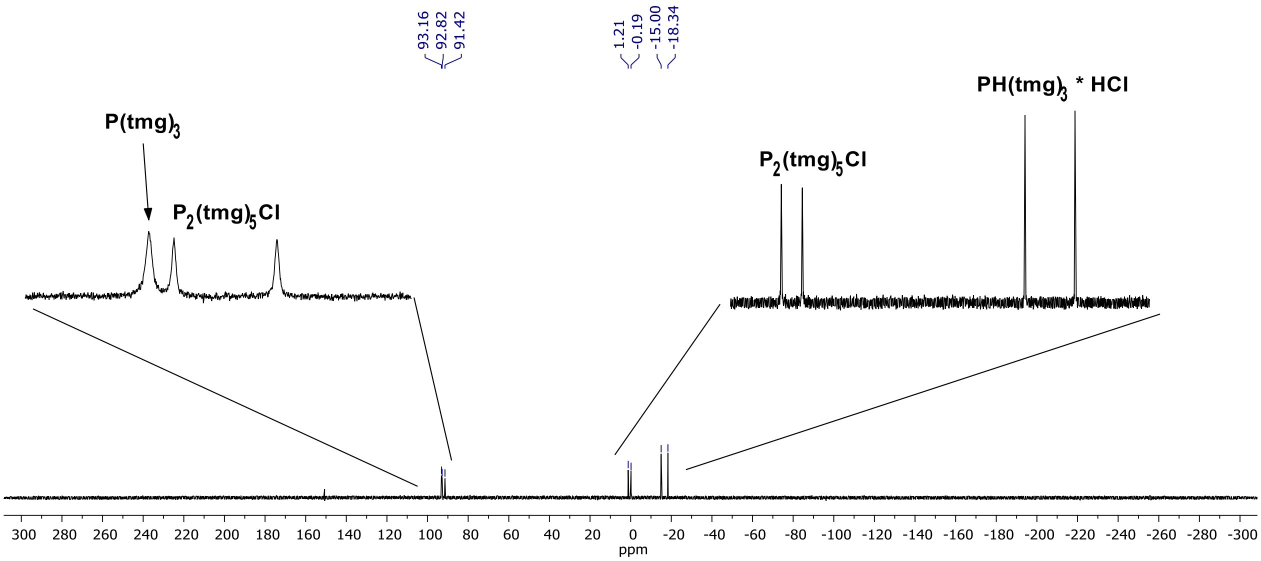

Figure S56: ^{31}P NMR spectrum (acetonitrile-d_3, 300 K, 162 MHz) of the reaction of phosphine **1** (1 eq.) and phosphonium salt **1**·HCl (1 eq.).

The independent formation of $P_2(tmg)_5Cl$ by the reaction of phosphine **1** (15 mg, 0.04 mmol, 1 eq.) and phosphonium salt **1**·HCl (16.5 mg, 0.04 mmol, 1 eq.) in THF-d_8 in an ^{31}P NMR experiment was performed (Figure S57) and addition to KHMDS (2 eq.) leads to the formation of the free phosphine **1** (Figure S58):

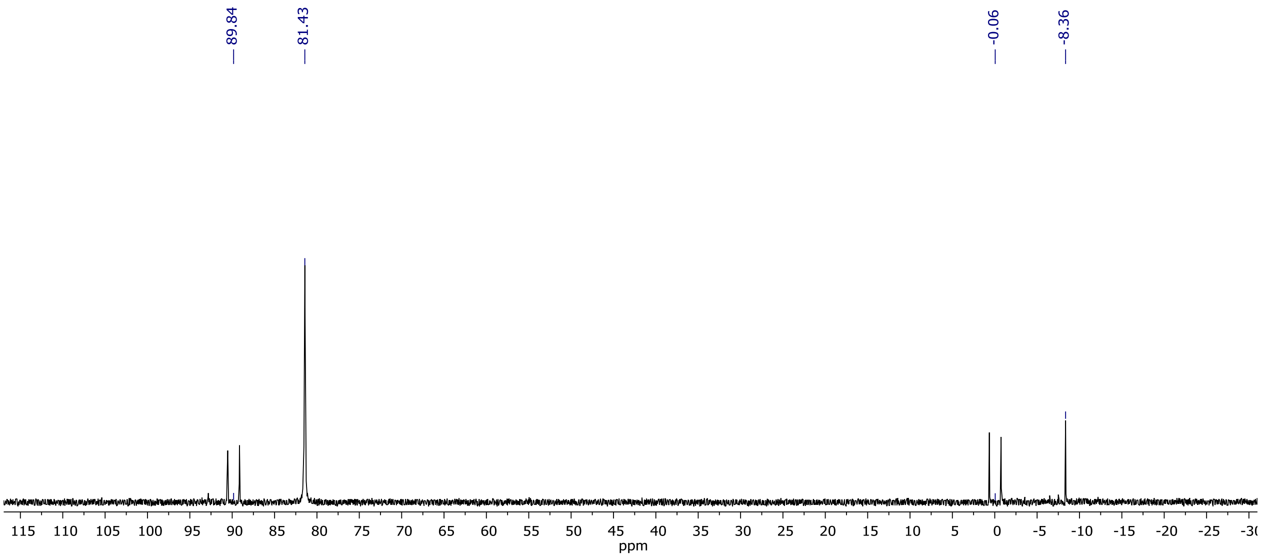

Figure S57: $^{31}P\{^{1}H\}$ NMR spectrum (THF-d_8, 300 K, 162 MHz) of the reaction of phosphine **1** (1 eq.) and phosphonium salt **1**·HCl (1 eq.).

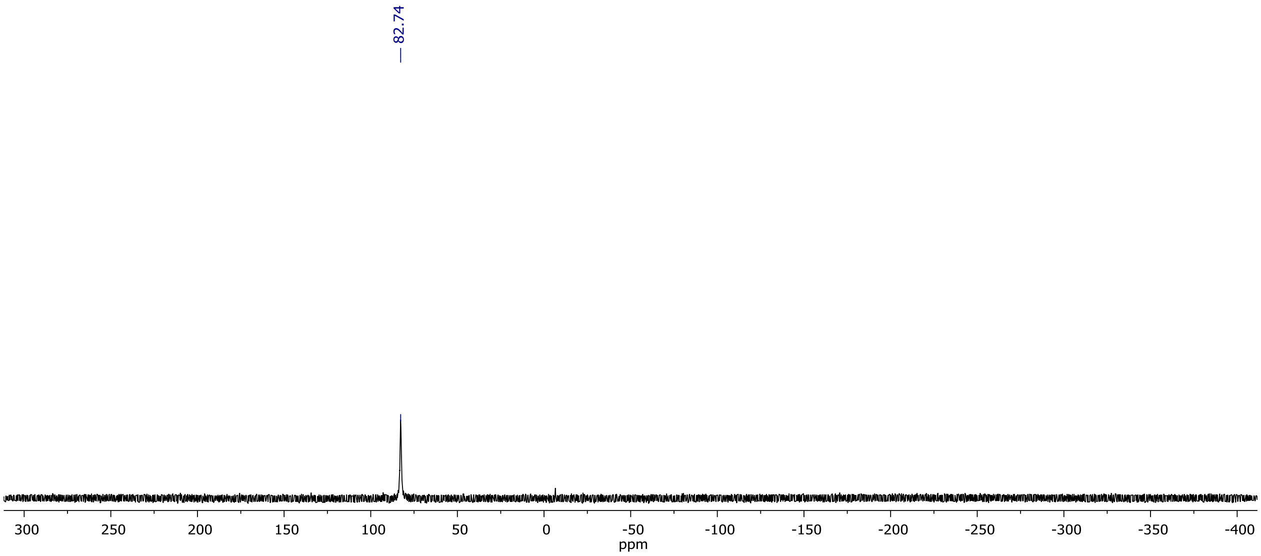

Figure S58: ^{31}P NMR spectrum (THF-d_8, 300 K, 162 MHz) of the reaction of phosphine **1** (1 eq.) and phosphonium salt **1**·HCl (1 eq.) after addition of KHMDS and heating at 80 °C for 1 hour.

X-ray Diffraction Studies

General:

Single-crystal X-ray diffraction data of **1**: Crystals were selected under oil, mounted on glass capillaries, and then immediately placed in a cold stream of N_2 on a diffractometer. Data were collected on a Bruker AXS detector using Mo-K_α radiation (λ = 0.71073 Å). Using Olex2,[7] the structures were solved with the Superflip[8] structure solution program using Charge Flipping and refined with the ShelXL[9] refinement package using Least Squares minimization.

Single-crystal X-ray diffraction data of **2**, **3** and **4**: Crystal data were collected on a Bruker D8 Venture Photon III Diffractometer system equipped with a micro focus tube Cu-K_α (λ = 1.54178 Å) and a MX mirror monochromator. The structure was solved and refined using the Bruker SHELXTL[10] Software Package.

Single-crystal X-ray diffraction data of **5** and **7**: Crystals were selected under oil, mounted on glass capillaries, and then immediately placed in a cold stream of N_2 on a diffractometer. Data were collected on a Bruker AXS detector using Mo-K_α radiation (λ = 0.71073 Å). Using Olex2,[7] the structures were solved with the ShelXT[10a] structure solution program using intrinsic phasing and refined with the ShelXL[10b] refinement package using Least Squares minimization.

Single-crystal X-ray diffraction data of **8** and **9**: Crystal data were collected on a Bruker D8 Venture Photon III Diffractometer system equipped with a micro focus tube Mo-K_α (λ = 0.71073 Å) and a MX mirror monochromator. The structure was solved and refined using the Bruker SHELXTL[10] Software Package.

Crystallographic data have been deposited with the Cambridge Crystallographic Data Centre as supplementary publication no. CCDC-2116781 (**1**), CCDC-2067476 (**2**), CCDC-2067477 (**3**), CCDC-2067478 (**4**), CCDC-2116782 (**5**), CCDC-2116780 (**7**), CCDC-2113987 (**8**), CCDC-2067479 (**9**). These data can be obtained free of charge via www.ccdc.cam.uk/data_request/cif (or from the CCDC, 12 Union Road, Cambridge CB2 1EZ, UK; fax: (+44) 1223-336-033; or deposit@ccdc.cam.ac.uk).

Single-crystal X-ray structure analysis of 1:

Single crystals were obtained by storing a THF solution of **1** for 12 hours at –40 °C. A Single-crystal X-ray structure analysis revealed that **1** crystallizes in the triclinic space group P-1. The asymmetric unit contains one molecule of **1**.

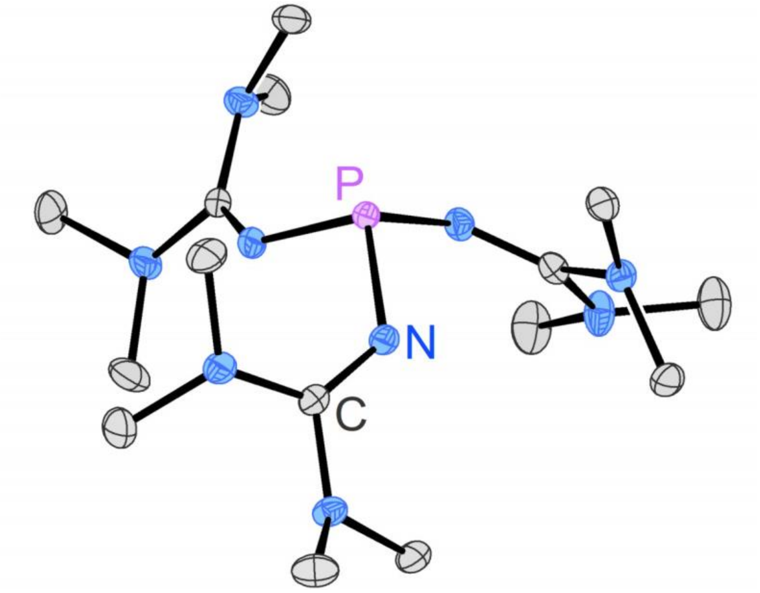

Figure S59: Molecular view of **1** in the solid state with thermal ellipsoid plot at the 50% levels of probability. Hydrogen atoms are omitted for clarity.

Table S1. Crystal data and structure refinement for **1**.

CCDC number	2116781	m/mm^{-1}	0.145
Empirical formula	$C_{15}H_{36}N_9P$	F(000)	408.0
Formula weight	373.50	Crystal size/mm^3	0.402 × 0.172 × 0.149
Temperature/K	100	Radiation	MoKα (λ = 0.71073)
Crystal system	triclinic	2Θ range for data collection	7.974 to 56.674
Space group	P-1	Index ranges	$-11 \leq h \leq 11$, $-14 \leq k \leq 14$, $-17 \leq l \leq 17$
a/Å	8.2833(4)	Reflections collected	14169
b/Å	10.6794(5)	Independent reflections	5193 [R_{int} = 0.0220, R_{sigma} = 0.0240]
c/Å	13.2693(6)	Data/restraints/parameters	5193/0/238
α/°	91.2000(10)	Goodness-of-fit on F^2	1.046
β/°	98.1050(10)	Final R indexes [I>=2σ (I)]	R_1 = 0.0335, wR_2 = 0.0880
γ/°	111.3460(10)	Final R indexes [all data]	R_1 = 0.0348, wR_2 = 0.0891
Volume/Å^3	1079.04(9)	Largest diff. peak/hole / e Å^{-3}	0.32/-0.32
ρ_{calc}mg/mm^3	1.150		

Single-crystal X-ray structure analysis of 2:

Single crystals were obtained by heating **2** for 3 hours in diethyl ether at 80 °C in a pressure tube and slowly cool down the mixture to room temperature. A Single-crystal X-ray structure analysis revealed that **2** crystallizes in the monoclinic space group $P2_1/c$. The asymmetric unit contains one molecule of **2**.

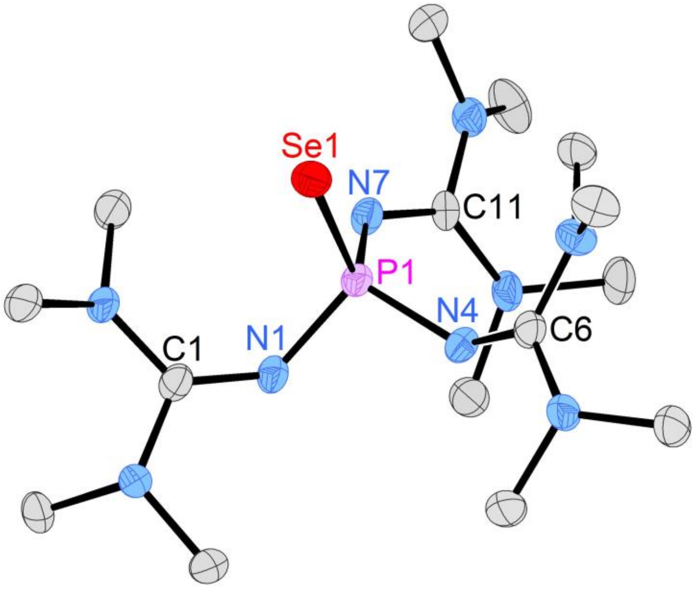

Figure S60: Molecular view of **2** in the solid state with thermal ellipsoid plot at the 50% levels of probability. Hydrogen atoms are omitted for clarity.

Table S2. Crystal data and structure refinement for **2**.

CCDC number	2067476	m/mm^{-1}	3.046
Empirical formula	$C_{15}H_{36}N_9PSe$	F(000)	952
Formula weight	452.46	Crystal size/mm^3	0.067 × 0.083 × 0.176
Temperature/K	102(2)	Radiation	CuKα (λ = 1.54178)
Crystal system	monoclinic	2Θ range for data collection	3.88 to 68.17
Space group	$P2_1/c$	Index ranges	$-13 \le h \le 13$, $-12 \le k \le 12$, $-22 \le l \le 22$
a/Å	11.4283(4)	Reflections collected	43070
b/Å	10.6278(3)	Independent reflections	4140 [R_{int} = 0.1112]
c/Å	18.7541(7)	Data/restraints/parameters	4140/0/247
α/°	90	Goodness-of-fit on F^2	1.028
β/°	93.802(2)	Final R indexes [I>=2σ (I)]	R_1 = 0.0460, wR_2 = 0.1074
γ/°	90	Final R indexes [all data]	R_1 = 0.0629, wR_2 = 0.1178
Volume/Å^3	2272.82(13)	Largest diff. peak/hole / e Å^{-3}	0.370/-0.86
ρ_{calc}mg/mm^3	1.322		

Single-crystal X-ray structure analysis of 3:

Single crystals were obtained by diffusion of *n*-hexane into a THF solution of **3**. A Single-crystal X-ray structure analysis revealed that **3** crystallizes in the monoclinic space group $P2_1/n$. The asymmetric unit contains one molecule of **3**.

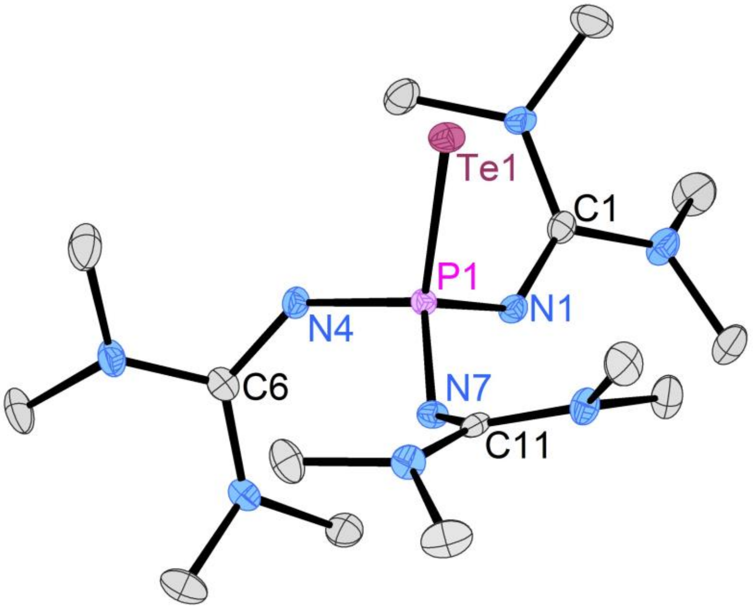

Figure S61: Molecular view of **3** in the solid state with thermal ellipsoid plot at the 50% levels of probability. Hydrogen atoms are omitted for clarity.

Table S3. Crystal data and structure refinement for **3**.

CCDC number	2067477	m/mm^{-1}	11.000
Empirical formula	$C_{15}H_{36}N_9PTe$	F(000)	1024
Formula weight	501.10	Crystal size/mm^3	0.029 × 0.123 × 0.153
Temperature/K	102(2)	Radiation	CuKα (λ = 1.54178)
Crystal system	monoclinic	2Θ range for data collection	2.90 to 68.29
Space group	$P2_1/n$	Index ranges	$-11 \leq h \leq 11$, $-9 \leq k \leq 9$, $-36 \leq l \leq 36$
a/Å	9.3081(2)	Reflections collected	45923
b/Å	8.1164(2)	Independent reflections	4017 [R_{int} = 0.0570]
c/Å	30.6452(7)	Data/restraints/parameters	4017/0/247
α/°	90	Goodness-of-fit on F^2	1.054
β/°	96.1080(10)	Final R indexes [I>=2σ (I)]	R_1 = 0.0223, wR_2 = 0.0517
γ/°	90	Final R indexes [all data]	R_1 = 0.0256, wR_2 = 0.0530
Volume/$Å^3$	2302.05(9)	Largest diff. peak/hole / e $Å^{-3}$	0.677/-0.466
$\rho_{calc}mg/mm^3$	1.446		

Single-crystal X-ray structure analysis of 4:

Single crystals were obtained by diffusion of *n*-hexane into a THF solution of **4**. A Single-crystal X-ray structure analysis revealed that **4** crystallizes in the trigonal space group P-3. The asymmetric unit contains 1/3 molecule of **4**. Note: A badly disordered half hexane molecule was found in the asymmetrical unit and could not be satisfactorily refined. The program SQUEEZE[11] was therefore used to remove mathematically the effect of the solvent. The quoted formula and derived parameters are not included the squeezed solvent molecule.

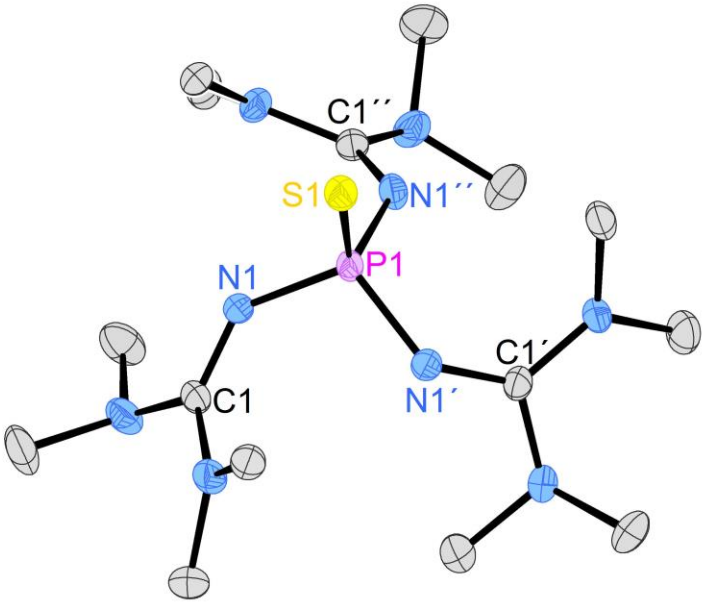

Figure S62: Molecular view of **4** in the solid state with thermal ellipsoid plot at the 50% levels of probability. Hydrogen atoms are omitted for clarity.

Table S4. Crystal data and structure refinement for **4**.

CCDC number	2067478	m/mm^{-1}	1.777
Empirical formula	$C_{15}H_{36}N_9PS$	F(000)	440
Formula weight	405.56	Crystal size/mm^3	0.048 × 0.064 × 0.239
Temperature/K	101(2)	Radiation	CuKα (λ = 1.54178)
Crystal system	trigonal	2Θ range for data collection	4.14 to 64.93
Space group	P-3	Index ranges	$-14 \le h \le 14$, $-14 \le k \le 14$, $-11 \le l \le 11$
a/Å	12.3391(5)	Reflections collected	25254
b/Å	12.3391(5)	Independent reflections	1520 [R_{int} = 0.2163]
c/Å	10.0568(9)	Data/restraints/parameters	1520/0/84
α/°	90	Goodness-of-fit on F^2	1.620
β/°	90	Final R indexes [I>=2σ (I)]	R_1 = 0.0977, wR_2 = 0.2335
γ/°	120	Final R indexes [all data]	R_1 = 0.1186, wR_2 = 0.2431
Volume/Å^3	1326.04(16)	Largest diff. peak/hole / e Å^{-3}	1.721/-0.512
ρ_{calc}mg/mm^3	1.016		

Single-crystal X-ray structure analysis of 5:

Single crystals were obtained by pressurizing a THF solution of **1** with 1 bar CO_2 for 3 hours. A Single-crystal X-ray structure analysis revealed that **5** crystallizes in the orthorombic space group *Pna*2_1. The asymmetric unit contains one molecule of **5**.

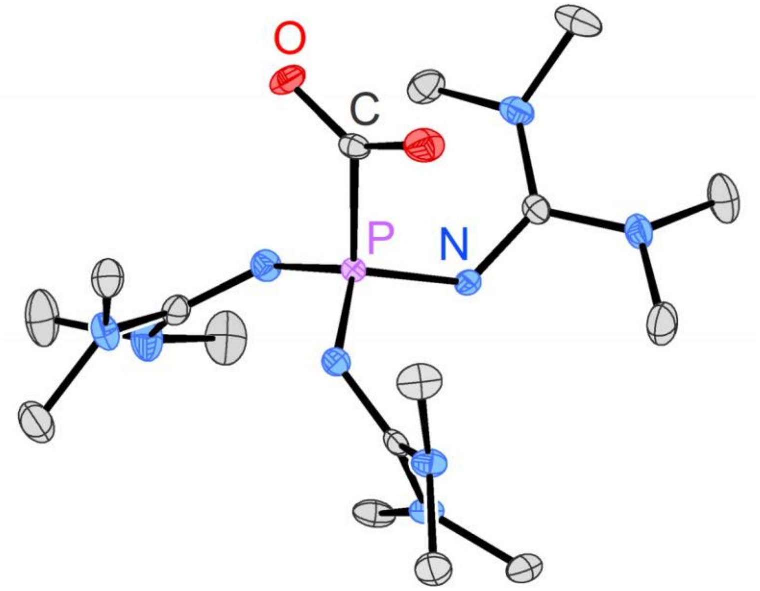

Figure S63: Molecular view of **5** in the solid state with thermal ellipsoid plot at the 50% levels of probability. Hydrogen atoms are omitted for clarity.

Table S5. Crystal data and structure refinement for **5**.

CCDC number	2116782	m/mm^{-1}	0.139
Empirical formula	$C_{20}H_{44}N_9O_3P$	F(000)	1064.0
Formula weight	489.61	Crystal size/mm^3	0.241 × 0.087 × 0.077
Temperature/K	100.0	Radiation	MoKα (λ = 0.71073)
Crystal system	orthorhombic	2Θ range for data collection	3.444 to 55.872
Space group	*Pna*2_1	Index ranges	$-11 \leq h \leq 11$, $-27 \leq k \leq 27$, $-18 \leq l \leq 18$
a/Å	9.0672(2)	Reflections collected	39976
b/Å	20.9180(5)	Independent reflections	6508 [R_{int} = 0.0707, R_{sigma} = 0.0452]
c/Å	14.3320(4)	Data/restraints/parameters	6508/1/310
α/°	90	Goodness-of-fit on F^2	1.043
β/°	90	Final R indexes [I>=2σ (I)]	R_1 = 0.0465, wR_2 = 0.1187
γ/°	90	Final R indexes [all data]	R_1 = 0.0526, wR_2 = 0.1243
Volume/Å^3	2718.32(12)	Largest diff. peak/hole / e Å^{-3}	0.76/-0.36
Z	4	Flack parameter	0.06(4)
ρ_{calc}mg/mm^3	1.196		

Single-crystal X-ray structure analysis of 7:

Single crystals were obtained by diffusion of *n*-hexane into a THF solution of **7**. A Single-crystal X-ray structure analysis revealed that **7** crystallizes in the monoclinic space group $P2_1/n$. The asymmetric unit contains one molecule of **7**.

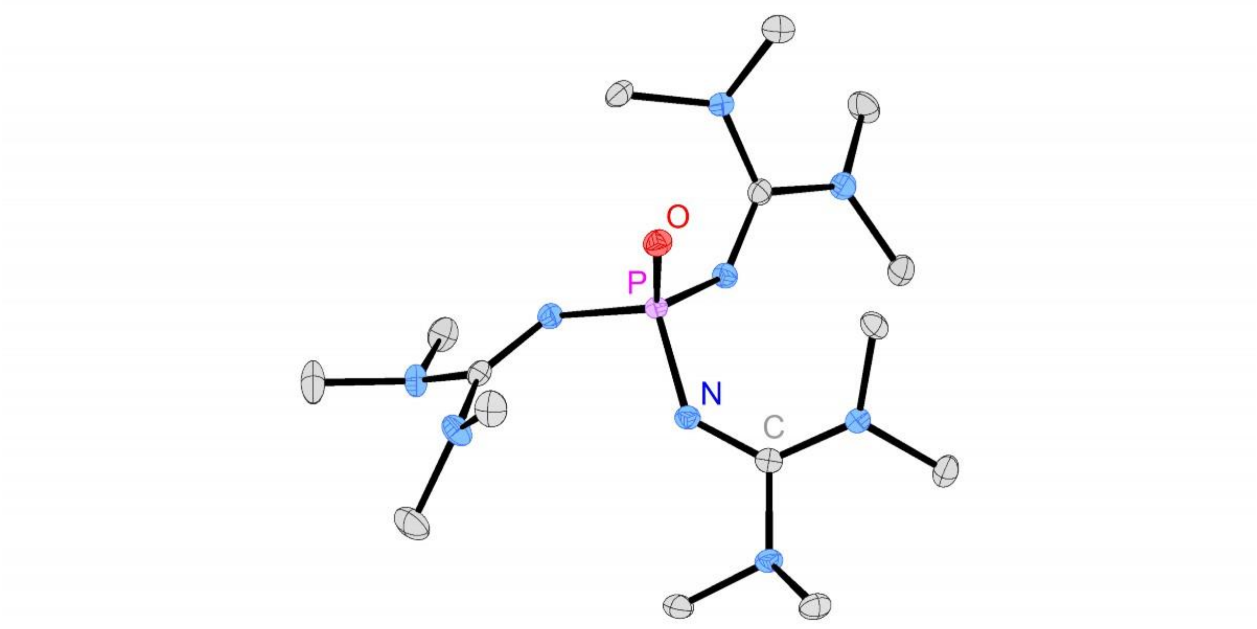

Figure S64: Molecular view of **7** in the solid state with thermal ellipsoid plot at the 50% levels of probability. Hydrogen atoms are omitted for clarity.

Table S6. Crystal data and structure refinement for **7**.

CCDC number	2116780	m/mm^{-1}	0.154
Empirical formula	$C_{15}H_{36}N_9OP$	F(000)	848.0
Formula weight	389.50	Crystal size/mm^3	0.519 × 0.148 × 0.087
Temperature/K	100	Radiation	MoKα (λ = 0.71073)
Crystal system	monoclinic	2Θ range for data collection	4.332 to 55.866
Space group	$P2_1/n$	Index ranges	$-14 \le h \le 14$, $-14 \le k \le 14$, $-23 \le l \le 23$
a/Å	10.9473(5)	Reflections collected	26635
b/Å	11.2184(5)	Independent reflections	5069 [R_{int} = 0.0526, R_{sigma} = 0.0282]
c/Å	17.8556(8)	Data/restraints/parameters	2505/0/247
α/°	90	Goodness-of-fit on F^2	1.048
β/°	105.1950(10)	Final R indexes [I>=2σ (I)]	R_1 = 0.0384, wR_2 = 0.0924
γ/°	90	Final R indexes [all data]	R_1 = 0.0474, wR_2 = 0.0967
Volume/Å^3	2116.20(17)	Largest diff. peak/hole / e Å^{-3}	0.41/-0.44
ρ_{calc}mg/mm^3	1.223		

Single-crystal X-ray structure analysis of 8:

Single crystals were obtained by diffusion of *n*-hexane into a THF solution of **8** at –40 °C. A Single-crystal X-ray structure analysis revealed that **8** crystallizes in the monoclinic space group *C*2/*c*. The asymmetric unit contains one molecule of **8**.

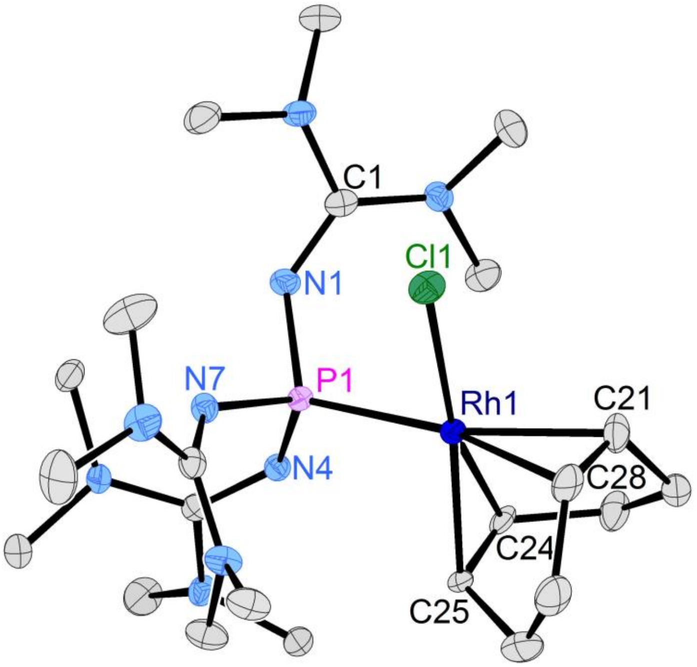

Figure S65: Molecular view of **8** in the solid state with thermal ellipsoid plot at the 50% levels of probability. Hydrogen atoms are omitted for clarity.

Table S7. Crystal data and structure refinement for **8**.

CCDC number	2113987	m/mm^{-1}	0.744
Empirical formula	$C_{23}H_{48}N_9PRhCl$	F(000)	2608
Formula weight	620.03	Crystal size/mm^3	0.057 × 0.116 × 0.170
Temperature/K	102(2)	Radiation	CuKα (λ = 1.54178)
Crystal system	monoclinic	2Θ range for data collection	3.04 to 26.74
Space group	*C*2/*c*	Index ranges	-27 ≤ h ≤ 27, -13 ≤ k ≤ 13, -32 ≤ l ≤ 32
a/Å	21.5739(7)	Reflections collected	56046
b/Å	11.0307(3)	Independent reflections	6317 [R_{int} = 0.0384]
c/Å	25.7251(8)	Data/restraints/parameters	6317/230/374
α/°	90	Goodness-of-fit on F^2	1.038
β/°	103.1020(10)	Final R indexes [I>=2σ (I)]	R_1 = 0.02357, wR_2 = 0.0526
γ/°	90	Final R indexes [all data]	R_1 = 0.0296, wR_2 = 0.0560
Volume/Å^3	5962.6(3)	Largest diff. peak/hole / e Å^{-3}	0.586/-0.614
ρ_{calc}mg/mm^3	1.381		

Single-crystal X-ray structure analysis of 9:

Single crystals were obtained by diffusion of *n*-hexane into a THF solution of **9**. A Single-crystal X-ray structure analysis revealed that **9** crystallizes in the monoclinic space group $P2_1/n$. The asymmetric unit contains one molecule of **9**.

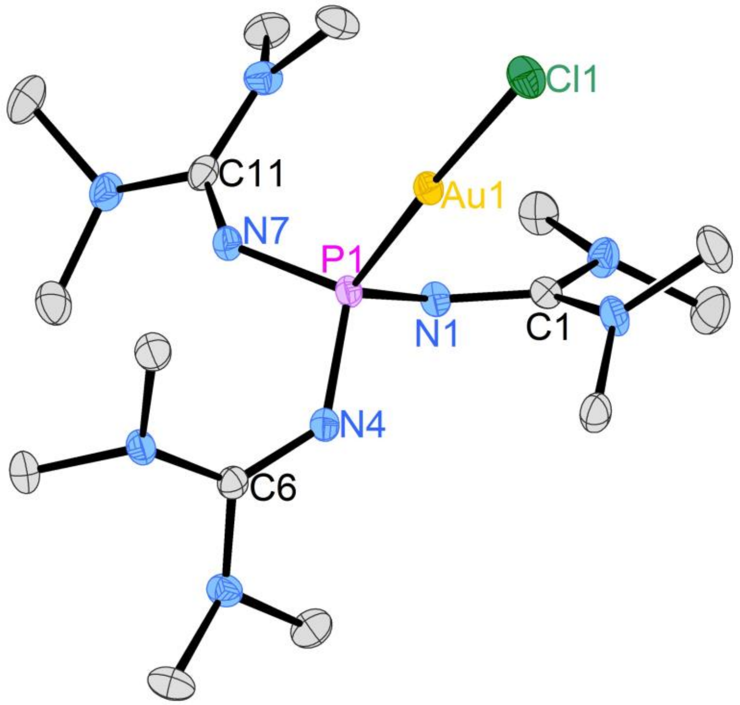

Figure S66: Molecular view of **9** in the solid state with thermal ellipsoid plot at the 50% levels of probability. Hydrogen atoms are omitted for clarity.

Table S8. Crystal data and structure refinement for **9**.

CCDC number	2067479	m/mm^{-1}	6.503
Empirical formula	$C_{15}H_{36}N_9PAuCl$	F(000)	1200
Formula weight	605.91	Crystal size/mm^3	0.033 × 0.108 × 0.163
Temperature/K	102(2)	Radiation	CuKα (λ = 1.54178)
Crystal system	monoclinic	2Θ range for data collection	2.26 to 27.50
Space group	$P2_1/n$	Index ranges	$-17 \le h \le 17$, $-14 \le k \le 14$, $-21 \le l \le 21$
a/Å	13.2818(4)	Reflections collected	75589
b/Å	10.9394(3)	Independent reflections	5283 [R_{int} = 0.0384]
c/Å	16.3193(4)	Data/restraints/parameters	5283/0/256
α/°	90	Goodness-of-fit on F^2	1.044
β/°	103.7800(10)	Final R indexes [I>=2σ (I)]	R_1 = 0.0127, wR_2 = 0.0291
γ/°	90	Final R indexes [all data]	R_1 = 0.0145, wR_2 = 0.0299
Volume/Å^3	2302.85(11)	Largest diff. peak/hole / e Å^{-3}	0.972/-0.247
ρ_{calc}mg/mm^3	1.748		

DFT Calculations

Calculation of Gas-phase basicity and proton affinity:

General: The geometry optimizations and frequency calculations were performed with Gaussian 09[12], using the wB97XD functional and the 6-31+G(d) basis set. The absence of any imaginary frequency confirmed that each optimized structure is at a local minimum. Single point energies were calculated using the 6-311+G(2df,p) basis set. (NIMe = 1,3-dimethylimidazolin-2-ylidenamino).

Structure	Sum of electronic and thermal Free Energies [E_h]	Sum of electronic and thermal Enthalpie [E_h]	GB[a] [kcal/mol]	PA[b] [kcal/mol]
$P(tmg)_3$ (**1**)	-1426.896597	-1426.803313	275.59	284.44
$HP(tmg)_3$	-1427.345802	-1427.254231		
$P(tmg)_2Me$	-1104.937164	-1104.862499	264.71	273.09
$HP(tmg)_2Me$	-1105.369020	-1105.295337		
$P(tmg)Ph_2$	-1166.327124	-1166.257464	246.56	253.96
$HP(tmg)Ph_2$	-1166.730058	-1166.659811		
$P(NIMe)_3$	-1423.381062	-1423.297395	276.77	283.93
$HP(NIMe)_3$	-1423.832140	-1423.747505		

[a] GB=[G(Phosphine)-G(Phosphine-H^+)+G(H^+)]·627.51 kcal·mol^{-1}·E_h^{-1}

[b] PA=[H(Phosphine)-H(Phosphine-H^+)+H(H^+)]·627.51 kcal·mol^{-1}·E_h^{-1}

Calculation of Tolman electronic parameter:

General: The Tolman electronic parameter was calculated and scaled as published by Gusev in 2009.[13] The geometry optimizations and frequency calculations were performed with Gaussian 09, using the mPW1PW91 functional and the 6-311+G(2d) basis set for Ni and 6-311+G(d,p) for all other atoms. The absence of any imaginary frequency confirmed that each optimized structure is at a local minimum.

Structure	Unscaled ν_{CO} (A1)	TEP [cm^{-1}]
$P(tmg)_3$ (**1**)	2142.80	2044.45
$P(tmg)_2Me$	2146.99	2048.44
$P(tmg)Ph_2$	2161.14	2061.94
$P(NIMe)_3$	2140.11	2041.88

XYZ Data of the optimized structures

P(tmg)$_3$

C	2.00608	4.23045	0.91951
N	1.71538	3.17365	-0.03324
H	1.30129	5.06741	0.79658
H	3.02318	4.61399	0.77156
H	1.91644	3.85479	1.93892
C	1.54784	3.64077	-1.39429
H	0.61249	4.20609	-1.52227
H	1.51873	2.79156	-2.07748
H	2.39281	4.29099	-1.64824
C	1.01217	2.03482	0.35394
N	1.39479	1.54737	1.59221
C	2.78780	1.37061	1.93945
H	3.42816	1.83594	1.18751
H	3.02722	0.29741	1.99044
H	3.01310	1.81841	2.91849
C	0.45584	0.88517	2.47577
H	0.58147	1.29348	3.48939
H	0.61120	-0.19907	2.48663
H	-0.56472	1.05918	2.13433
N	0.12224	1.55839	-0.44913
P	-0.35488	-0.06908	-0.69462
N	-1.68737	-0.22356	0.37848
N	0.75937	-1.02117	0.21217
C	1.69465	-1.73189	-0.30995
N	2.36214	-1.51612	-1.51075
N	2.13951	-2.86821	0.36843
C	2.53535	-0.18775	-2.05906
H	1.77329	0.06659	-2.80775
H	3.52530	-0.13035	-2.53193
H	2.49583	0.55378	-1.26000
C	2.66057	-2.60071	-2.42397
H	2.09444	-2.47459	-3.35792
H	2.38056	-3.55450	-1.97515
H	3.73118	-2.62829	-2.67556
C	1.34882	-3.30098	1.50362
H	0.28796	-3.21959	1.26683
H	1.54265	-2.68783	2.39744
H	1.60013	-4.34381	1.72764
C	3.56478	-3.09454	0.53826
H	4.12613	-2.62389	-0.26925
H	3.78089	-4.16932	0.53723
H	3.91781	-2.67336	1.49389
C	-2.91719	-0.26804	0.00231
N	-3.84979	-0.90004	0.83078
N	-3.46908	0.24561	-1.16128
C	-2.91017	1.39424	-1.84556
H	-3.72624	2.08478	-2.10420
H	-2.38988	1.10807	-2.76891
H	-2.19894	1.91649	-1.20365
C	-4.53571	-0.42606	-1.87369
H	-5.42881	0.21150	-1.95923
H	-4.80910	-1.34807	-1.35954
H	-4.20219	-0.67634	-2.89061
C	-5.06836	-0.19094	1.18743
H	-4.92460	0.39959	2.10690
H	-5.88262	-0.90424	1.35982
H	-5.36582	0.49091	0.39029
C	-3.30940	-1.68557	1.92397
H	-4.10006	-2.34277	2.30321
H	-2.94283	-1.05184	2.74588
H	-2.47196	-2.28590	1.56788

HP(tmg)$_3$

C	3.34397	3.74904	0.32106
N	2.60436	2.69807	-0.36893

H	3.12443	4.71086	-0.15548
H	4.42499	3.57724	0.27397
H	3.03595	3.80944	1.36495
C	2.50147	2.87132	-1.80928
H	1.77280	3.65030	-2.06694
H	2.18845	1.93957	-2.27919
H	3.48358	3.15893	-2.19501
C	1.70821	1.89691	0.27354
N	1.99861	1.57109	1.56646
C	3.35441	1.27891	2.00772
H	4.04589	1.30841	1.16504
H	3.37403	0.26940	2.43524
H	3.69370	1.98601	2.77414
C	0.95905	1.23596	2.52788
H	1.23488	1.66987	3.49494
H	0.84448	0.15146	2.63424
H	0.00340	1.65876	2.21587
N	0.63530	1.51892	-0.38706
P	-0.22599	0.15136	-0.27150
N	-1.60908	0.35198	0.55368
N	0.57862	-1.09367	0.40664
C	1.03528	-2.17442	-0.17952
N	1.49143	-2.23629	-1.47385
N	1.08262	-3.32829	0.53749
C	2.16192	-1.10999	-2.10014
H	1.51881	-0.59092	-2.82260
H	3.04724	-1.47615	-2.63249
H	2.48860	-0.39886	-1.33988
C	1.26925	-3.38894	-2.33379
H	0.81361	-3.05046	-3.27167
H	0.58496	-4.09005	-1.85472
H	2.20447	-3.90936	-2.57513
C	0.34322	-3.42063	1.78678
H	-0.56587	-2.82406	1.72318
H	0.94109	-3.05361	2.63063
H	0.08412	-4.46872	1.96228
C	2.13869	-4.31840	0.36856
H	2.89225	-3.95732	-0.33181
H	1.74196	-5.27458	0.00964
H	2.62530	-4.48658	1.33549
C	-2.83287	0.45331	0.09136
N	-3.85580	0.05115	0.89412
N	-3.17626	0.93607	-1.14577
C	-2.41802	1.98622	-1.80796
H	-3.10855	2.77481	-2.12981
H	-1.89315	1.60832	-2.69432
H	-1.68578	2.42077	-1.12691
C	-4.30248	0.41600	-1.90607
H	-5.11525	1.14796	-1.99311
H	-4.68786	-0.49100	-1.43913
H	-3.95988	0.16585	-2.91685
C	-5.13772	0.74476	0.93234
H	-5.32707	1.08488	1.95641
H	-5.96034	0.08894	0.62638
H	-5.11815	1.62166	0.28487
C	-3.55837	-0.75517	2.06753
H	-4.44761	-1.33891	2.32211
H	-3.28200	-0.12757	2.92409
H	-2.72954	-1.42865	1.85239
H	-0.54270	-0.17968	-1.60790

$P(tmg)_2Me$

C	3.95924	-0.88575	-1.62714
C	4.62409	1.32551	0.45548
N	3.15923	-0.96691	-0.42231
C	2.49037	0.13615	0.09499
N	3.23002	1.30940	0.04843
C	2.89931	-2.30874	0.05217
H	3.85458	-2.84491	0.14152
H	2.24001	-2.87315	-0.61954

H	2.43726	-2.26799	1.04060
C	2.52370	2.54904	0.31118
H	3.10351	3.37664	-0.11305
H	2.38908	2.72078	1.38933
H	1.53603	2.51263	-0.14901
H	3.99156	0.14418	-1.98479
H	3.51880	-1.51426	-2.41414
H	4.98841	-1.23191	-1.45070
H	5.04489	0.32022	0.41822
H	4.72117	1.70043	1.48609
H	5.20967	1.97748	-0.20401
N	1.30444	0.16908	0.60016
P	0.03807	-0.88964	0.12683
N	-1.13273	0.30742	-0.21131
C	-2.41097	0.22993	-0.17894
N	-3.19451	-0.92530	-0.18727
C	-4.30553	-1.09508	0.72570
H	-4.07449	-1.86588	1.47744
H	-5.21467	-1.40546	0.19100
H	-4.50574	-0.15611	1.24357
C	-2.73837	-2.12638	-0.84722
H	-2.24761	-2.83775	-0.16417
H	-2.03163	-1.87371	-1.63916
H	-3.60241	-2.63109	-1.29812
N	-3.16365	1.40490	-0.11950
C	-2.42657	2.63237	0.11278
H	-1.68318	2.47386	0.89482
H	-3.13314	3.40894	0.42588
H	-1.89745	2.97163	-0.79076
C	-4.29482	1.57207	-1.01767
H	-5.04026	2.23146	-0.55893
H	-4.76261	0.60985	-1.22825
H	-3.97910	2.01949	-1.97428
C	-0.49593	-1.39774	1.83227
H	-1.47155	-1.89493	1.80214
H	-0.56134	-0.51097	2.47264
H	0.23231	-2.09330	2.26424

$HP(tmg)_2Me$

C	2.49742	1.99658	-1.13556
C	4.67248	0.50780	0.29859
N	2.27122	0.55919	-1.21100
C	2.35769	-0.24581	-0.11194
N	3.38516	-0.01870	0.73848
C	1.62073	0.10033	-2.42495
H	2.11643	0.56826	-3.28072
H	0.55689	0.37343	-2.44893
H	1.73369	-0.98101	-2.52617
C	3.38744	-0.64487	2.05342
H	3.99372	-0.03255	2.72604
H	3.80714	-1.65658	2.00927
H	2.37052	-0.70631	2.43943
H	2.72563	2.29103	-0.11103
H	1.58146	2.51383	-1.44472
H	3.31702	2.31112	-1.79148
H	4.70239	0.57839	-0.78876
H	5.46165	-0.17937	0.62051
H	4.87220	1.49404	0.73139
N	1.53628	-1.24575	0.16289
P	-0.03629	-1.36945	-0.15841
N	-0.79298	0.06625	-0.18542
C	-2.06395	0.34605	0.03831
N	-3.10904	-0.44724	-0.34827
C	-4.32082	-0.60484	0.44635
H	-4.49702	-1.67373	0.61318
H	-5.19959	-0.18642	-0.05759
H	-4.20040	-0.12325	1.41715
C	-3.00559	-1.30727	-1.51499
H	-2.76355	-2.34700	-1.25320
H	-2.25009	-0.92076	-2.20122

H	-3.96834	-1.30954	-2.03506
N	-2.36395	1.50686	0.66689
C	-1.31560	2.23571	1.36679
H	-0.60545	1.53738	1.80837
H	-1.77769	2.83251	2.15759
H	-0.77401	2.90159	0.68389
C	-3.56674	2.27903	0.37239
H	-4.24074	2.32036	1.23457
H	-4.09549	1.85125	-0.47948
H	-3.27238	3.30090	0.11230
C	-0.72252	-2.48914	1.08270
H	-1.78633	-2.66852	0.90222
H	-0.59427	-2.03971	2.07160
H	-0.18145	-3.43881	1.05448
H	-0.21776	-2.05449	-1.37925

P(tmg)Ph_2

C	-2.53660	0.29609	-2.03549
C	-3.42713	-2.67468	0.50244
N	-2.57033	-0.25142	-0.69759
H	-3.56696	0.39920	-2.39937
H	-2.05936	1.28540	-2.07958
H	-2.00137	-0.38087	-2.70383
N	-2.10001	-2.09861	0.63420
H	-3.91422	-2.73970	1.48260
H	-3.36940	-3.68769	0.07659
H	-4.04075	-2.05922	-0.15548
C	-3.36184	0.49396	0.26342
H	-3.01546	1.53658	0.31605
H	-3.25829	0.04863	1.25398
H	-4.42382	0.48991	-0.01824
C	-1.62206	-1.17932	-0.28324
C	-1.13832	-2.95197	1.30214
H	-1.60774	-3.37321	2.19739
H	-0.26323	-2.36849	1.59269
H	-0.80028	-3.77548	0.65605
N	-0.38603	-1.24581	-0.64618
P	0.53799	0.02874	-1.30144
C	-0.15832	2.70445	-0.73399
C	-0.52757	3.77871	0.07849
C	-0.64162	3.60127	1.45406
C	-0.37496	2.35109	2.01675
C	-0.00125	1.28698	1.20360
C	0.10429	1.44872	-0.18372
H	0.21247	0.31635	1.64560
H	-0.45389	2.21059	3.09158
H	-0.72723	4.75041	-0.36523
H	-0.93190	4.43374	2.08927
H	-0.07766	2.84489	-1.81022
C	2.17208	-0.41020	-0.56290
C	3.19221	0.54902	-0.56482
C	4.45472	0.24464	-0.06550
C	4.72106	-1.03123	0.43223
C	2.44815	-1.68725	-0.06954
C	3.71582	-1.99477	0.42558
H	2.99616	1.54919	-0.94741
H	5.23245	1.00373	-0.06431
H	5.70742	-1.27126	0.81978
H	1.65947	-2.43344	-0.07467
H	3.91638	-2.99235	0.80818

HP(tmg)Ph_2

C	2.37143	0.06572	2.17828
C	3.42755	-2.72576	-0.62285
N	2.54597	-0.43566	0.82490

H	3.34783	0.08406	2.67219
H	1.96743	1.08670	2.19052
H	1.71982	-0.60058	2.74639
N	2.07722	-2.17181	-0.64614
H	4.01231	-2.39513	-1.48750
H	3.35572	-3.81734	-0.64726
H	3.94089	-2.43688	0.29422
C	3.62225	0.18273	0.05966
H	3.54477	1.27060	0.16712
H	3.52080	-0.06097	-0.99808
H	4.60907	-0.13665	0.41285
C	1.63581	-1.27862	0.26114
C	1.12713	-2.88139	-1.49440
H	1.62377	-3.13257	-2.43507
H	0.26144	-2.25158	-1.69706
H	0.78424	-3.80333	-1.01120
N	0.33985	-1.28801	0.56116
P	-0.57413	0.00195	0.86962
C	0.12157	2.70074	0.79882
C	0.55942	3.85926	0.16067
C	0.84660	3.83560	-1.20224
C	0.69158	2.65807	-1.93549
C	0.25028	1.50042	-1.30523
C	-0.03259	1.52043	0.06616
H	0.11951	0.58641	-1.88014
H	0.90859	2.64591	-2.99896
H	0.67472	4.77765	0.72749
H	1.18794	4.73916	-1.69788
H	-0.09946	2.72493	1.86362
C	-2.25848	-0.39245	0.39125
C	-3.18983	0.64007	0.23050
C	-4.50295	0.33172	-0.10786
C	-4.88294	-0.99841	-0.28488
C	-2.63647	-1.72684	0.21402
C	-3.95302	-2.02424	-0.12611
H	-2.89505	1.67948	0.35363
H	-5.22787	1.12874	-0.23843
H	-5.90856	-1.23528	-0.55067
H	-1.90241	-2.51740	0.33513
H	-4.25275	-3.05795	-0.26641
H	-0.60598	0.35885	2.23331

$P(NIMe)_3$

C	1.14484	-4.46867	-0.11781
C	-0.01286	-4.62451	0.86031
N	1.17709	-3.02943	-0.32355
H	0.93367	-4.99711	-1.06291
H	2.09807	-4.83017	0.28032
C	-0.09099	-2.50062	-0.07947
N	-0.87141	-3.53278	0.45190
H	0.32856	-4.49771	1.90254
H	-0.52121	-5.58976	0.77325
C	2.00608	-2.55155	-1.40948
H	2.99683	-3.00764	-1.31317
H	2.12453	-1.47021	-1.34630
H	1.58842	-2.81884	-2.39294
C	-2.01169	-3.19798	1.27208
H	-2.68680	-4.05844	1.32210
H	-2.52912	-2.34900	0.82239
H	-1.70963	-2.91849	2.29466
N	-0.57467	-1.33389	-0.24148
P	0.01161	0.12401	-0.88293
N	1.42586	0.32326	0.04447
C	2.31819	1.23563	0.04895
N	3.45679	1.11530	0.84924
N	2.44198	2.43476	-0.64828
C	4.44313	2.09897	0.45912

C	3.55305	3.19910	-0.10715
H	5.12283	1.70135	-0.31499
H	5.04147	2.42839	1.31429
H	4.04143	3.78744	-0.88999
H	3.21275	3.88252	0.68887
C	1.31719	3.18139	-1.17188
H	0.72348	3.64931	-0.37394
H	0.66237	2.53297	-1.75191
H	1.70641	3.95894	-1.83726
C	3.89044	-0.19251	1.27531
H	4.50773	-0.09912	2.17480
H	4.47804	-0.70192	0.49305
H	3.00692	-0.79465	1.49481
N	-1.01007	1.22789	-0.08288
C	-2.27956	1.33575	-0.03078
N	-3.30764	0.57058	-0.58248
N	-2.88347	2.36721	0.69882
C	-4.28892	2.07050	0.88038
C	-4.57810	1.24423	-0.36542
H	-5.38711	0.52063	-0.22533
H	-4.83067	1.89577	-1.21902
H	-4.88789	2.98461	0.93877
H	-4.45810	1.47815	1.79670
C	-3.15388	-0.12810	-1.84189
H	-4.06455	-0.70795	-2.02278
H	-2.31551	-0.82288	-1.79138
H	-3.00536	0.56691	-2.68270
C	-2.16093	2.97046	1.79328
H	-2.60316	3.94430	2.02728
H	-1.12015	3.10136	1.49520
H	-2.18554	2.33829	2.69592

$HP(NIMe)_3$

C	-3.85031	-2.78322	-0.26796
C	-4.29142	-1.95955	0.93964
N	-2.51464	-2.25105	-0.53068
H	-4.50507	-2.60925	-1.13416
H	-3.80908	-3.85556	-0.06462
C	-2.41129	-0.99401	-0.00446
N	-3.51430	-0.74340	0.75940
H	-4.02154	-2.45049	1.88648
H	-5.36323	-1.74777	0.94553
C	-1.82440	-2.67853	-1.73290
H	-2.04138	-3.73764	-1.88877
H	-0.74511	-2.57665	-1.60948
H	-2.16135	-2.11878	-2.61632
C	-3.54118	0.29583	1.76439
H	-4.56727	0.65240	1.88766
H	-2.90581	1.11888	1.43621
H	-3.17209	-0.07502	2.73071
N	-1.48374	-0.09058	-0.14775
P	0.00915	-0.07428	-0.76853
N	0.86121	-1.35679	-0.28993
C	2.13478	-1.53716	-0.05171
N	2.55970	-2.36241	0.94482
N	3.22144	-1.05424	-0.70765
C	3.98456	-2.62510	0.81975
C	4.44653	-1.42414	-0.01016
H	4.15684	-3.57511	0.29268
H	4.46302	-2.67462	1.80055
H	5.23662	-1.67635	-0.72152
H	4.79227	-0.59722	0.62560
C	3.22744	-0.06603	-1.76138
H	4.10387	-0.24003	-2.39170
H	3.25011	0.95581	-1.36866
H	2.33808	-0.18288	-2.38380
C	1.68456	-3.28617	1.62885

H	2.03448	-3.42407	2.65548
H	1.66325	-4.26187	1.12412
H	0.67707	-2.86902	1.64045
N	0.75822	1.29482	-0.35637
C	0.30909	2.49455	-0.09262
N	-0.69933	3.19932	-0.66876
N	0.90377	3.28004	0.84857
C	0.42512	4.64972	0.75070
C	-0.91657	4.45462	0.03946
H	-1.74603	4.35596	0.75342
H	-1.14814	5.26007	-0.66159
H	1.11807	5.25982	0.15264
H	0.32103	5.10170	1.73973
C	-1.65323	2.71109	-1.63780
H	-2.00029	3.55818	-2.23572
H	-2.50988	2.21954	-1.16433
H	-1.16759	2.00331	-2.31229
C	2.18998	2.97354	1.42892
H	3.00971	3.39861	0.83280
H	2.30324	1.88961	1.47313
H	2.23709	3.38453	2.44090
H	-0.10641	-0.13440	-2.17503

P(tmg)$_3$-Ni(CO)$_3$

Ni	0.50802	0.67152	2.19036
C	-4.59356	0.62274	-0.85749
C	-3.54151	3.36342	-1.73472
N	-3.32915	0.92710	-0.22620
C	-2.25688	1.43337	-0.92075
N	-2.62075	2.26739	-1.97800
N	-1.00686	1.24093	-0.69887
H	-4.53434	0.81085	-1.92701
H	-5.41341	1.21900	-0.43779
H	-4.83855	-0.43355	-0.70078
H	-4.19238	3.14006	-0.89317
H	-4.16207	3.54537	-2.61698
H	-2.99497	4.28821	-1.50397
P	-0.05329	0.08531	0.05648
N	-0.94686	-1.33381	-0.08069
C	-0.66270	-2.57218	-0.26866
N	0.53143	-3.21714	-0.03400
N	-1.65308	-3.41763	-0.77206
C	-1.99471	-4.61845	-0.03036
H	-2.30123	-5.41652	-0.71274
H	-2.82333	-4.42536	0.66501
H	-1.14595	-4.96841	0.55180
C	-2.79705	-2.79258	-1.39548
H	-3.28259	-3.52248	-2.04916
H	-2.47353	-1.93685	-1.98310
H	-3.53140	-2.44375	-0.65785
C	0.94703	-4.39460	-0.76327
H	0.95229	-5.29327	-0.13280
H	1.96679	-4.25192	-1.13602
H	0.28639	-4.56313	-1.61038
C	1.46841	-2.75353	0.95948
H	1.69057	-3.56112	1.66704
H	1.04944	-1.91759	1.51340

H	2.41069	-2.42533	0.50801
N	1.13686	-0.13086	-1.12718
C	2.28384	0.42497	-1.30958
C	-1.59382	2.58840	-2.94476
H	-2.07451	2.91601	-3.87056
H	-0.98571	1.70840	-3.14123
H	-0.92998	3.39009	-2.59456
C	-3.20710	0.48265	1.14132
H	-2.28825	0.86805	1.57669
H	-3.18317	-0.60814	1.21328
H	-4.05358	0.86630	1.72134
N	3.19672	-0.21591	-2.14540
N	2.75821	1.60014	-0.76752
C	2.88518	-1.56711	-2.55389
H	3.80512	-2.05565	-2.88609
H	2.15492	-1.59066	-3.37344
H	2.45775	-2.11246	-1.71880
C	3.85165	0.54588	-3.19402
H	3.30930	0.43954	-4.14350
H	4.87525	0.19135	-3.34541
H	3.88534	1.60277	-2.94487
C	4.15808	1.81401	-0.47206
H	4.71156	0.88324	-0.57146
H	4.25760	2.16748	0.55852
H	4.61132	2.56895	-1.12848
C	1.90932	2.74806	-0.53880
H	0.87451	2.51290	-0.78038
H	2.24209	3.58410	-1.16974
H	1.96176	3.06972	0.50460
C	-0.42186	-0.43325	3.25128
C	2.27444	0.47808	2.40567
C	-0.03550	2.37319	2.35820
O	-1.02461	-1.14426	3.91308
O	3.39944	0.33926	2.56172
O	-0.40286	3.45148	2.44897

$P(tmg)_2Me\text{-}Ni(CO)_3$

N	3.45102	0.17120	-0.54328
C	2.54659	1.07491	-0.02002
N	3.11360	2.27785	0.37679
N	1.27852	0.91456	0.11443
P	0.12692	-0.05164	-0.65231
N	-1.13201	1.02536	-0.95152
C	-2.20117	1.28644	-0.27520
N	-2.39032	1.16746	1.07955
N	-3.31496	1.73805	-0.96819
C	3.29802	-1.25101	-0.35576
H	3.28792	-1.78215	-1.31370
H	2.36751	-1.46675	0.16547
H	4.13032	-1.64940	0.23786
C	4.62378	0.57630	-1.28486
H	4.60164	1.64939	-1.46084

H	4.63808	0.06329	-2.25317
H	5.55509	0.32341	-0.76185
C	4.28382	2.27567	1.23572
H	4.95170	3.10268	0.97849
H	4.83299	1.34277	1.13620
H	3.99189	2.38566	2.28859
C	2.21746	3.38724	0.62131
H	1.42469	3.39092	-0.12314
H	2.78623	4.31825	0.55492
H	1.75279	3.33155	1.61424
C	-3.68270	0.88908	1.66901
C	-1.30993	1.30639	2.03115
H	-0.37669	1.53743	1.52197
H	-1.17291	0.38622	2.60599
H	-1.54583	2.11487	2.73508
C	-3.32126	1.56346	-2.40490
H	-2.94679	0.57516	-2.65948
H	-2.69569	2.30658	-2.91488
H	-4.34994	1.66067	-2.76062
C	-3.99439	2.94345	-0.53010
H	-3.78524	3.14537	0.51733
H	-5.07657	2.84720	-0.65703
H	-3.65640	3.80934	-1.11494
H	-3.58727	0.04021	2.35297
H	-4.40189	0.62979	0.89614
H	-4.06622	1.74065	2.24537
C	0.71649	-0.20545	-2.38265
H	0.92382	0.78769	-2.78576
H	-0.07784	-0.66942	-2.96860
H	1.60660	-0.83064	-2.44286
Ni	-0.42164	-2.03627	0.28895
C	0.31810	-3.29927	-0.74524
C	0.22280	-2.02635	1.96085
C	-2.21076	-2.14638	0.21136
O	0.78536	-4.09696	-1.41736
O	0.65102	-1.99987	3.02036
O	-3.34786	-2.22925	0.13724

P(tmg)Ph_2-$Ni(CO)_3$

N	2.93989	-0.63682	0.34729
C	2.31315	0.50519	0.79094
N	3.17322	1.48077	1.26252
N	1.04635	0.74725	0.79941
P	-0.26402	0.09033	-0.01348
C	2.29576	-1.92682	0.41868
H	2.00994	-2.29962	-0.57013
H	1.40474	-1.86276	1.03973
H	2.97869	-2.65107	0.87601
C	4.20536	-0.61039	-0.35398
H	4.54480	0.41578	-0.47548
H	4.08319	-1.05614	-1.34722
H	4.97823	-1.17733	0.17886

C	4.25229	1.13081	2.16894
H	5.15636	1.69829	1.93059
H	4.47900	0.06990	2.10341
H	3.97025	1.35467	3.20564
C	2.63945	2.80861	1.47712
H	1.95274	3.06739	0.67456
H	3.46830	3.52051	1.48861
H	2.09770	2.88559	2.42809
Ni	-1.54294	-1.39170	1.10817
C	-1.01659	-3.08669	0.86137
C	-1.35067	-0.85118	2.81319
C	-3.22154	-1.13989	0.50262
O	-0.70214	-4.17598	0.71793
O	-4.28458	-0.99118	0.11954
C	0.28981	-0.34613	-1.71827
C	-0.18316	-1.50284	-2.33434
C	0.21401	-1.82758	-3.62821
C	1.09549	-1.00055	-4.31159
C	1.57309	0.15753	-3.70360
C	1.16847	0.48570	-2.41730
H	-0.86755	-2.14871	-1.79542
H	-0.16464	-2.72764	-4.09869
H	1.40868	-1.25335	-5.31804
H	2.25508	0.80912	-4.23820
H	1.52838	1.39793	-1.95427
O	-1.21766	-0.50192	3.89022
C	-1.21890	1.62623	-0.35189
C	-1.19172	2.68170	0.55968
C	-2.06107	1.70799	-1.46177
C	-2.85145	2.83246	-1.66318
C	-1.98404	3.80528	0.35581
C	-2.81514	3.88451	-0.75533
H	-2.09532	0.89651	-2.18029
H	-0.54065	2.61585	1.42233
H	-1.95296	4.62084	1.06961
H	-3.49605	2.88656	-2.53298
H	-3.43301	4.76093	-0.91282

$P(NIMe)_3$-$Ni(CO)_3$

C	-1.92874	-4.24638	-1.40562
C	-2.61663	-4.45423	-0.06520
N	-1.51064	-2.86161	-1.31912
H	-2.59435	-4.40036	-2.25751
H	-1.06141	-4.91291	-1.51843
C	-1.39446	-2.47497	-0.00482
N	-1.90658	-3.50980	0.76362
H	-2.51788	-5.47408	0.31208
H	-3.68854	-4.20802	-0.12409
C	-0.87724	-2.25798	-2.46512
H	-1.50351	-2.44963	-3.34119
H	0.12250	-2.66748	-2.64472
H	-0.79190	-1.18243	-2.32616

C	-2.30519	-3.29805	2.12890
H	-2.20341	-4.22872	2.69160
H	-3.34671	-2.95483	2.20481
H	-1.65712	-2.53910	2.56147
N	-0.96715	-1.40359	0.56176
P	0.00253	-0.11951	0.09516
N	1.26066	-0.89485	-0.69421
C	2.47499	-0.56535	-0.93466
N	3.46099	-1.52499	-1.11408
N	3.06870	0.66753	-1.07176
C	4.76859	-0.90722	-1.05134
C	4.45928	0.52702	-1.45577
H	5.47854	-1.39325	-1.72398
H	5.17030	-0.95292	-0.02889
H	4.57075	0.67348	-2.54021
H	5.09001	1.25709	-0.94416
C	2.37368	1.86165	-1.47990
H	2.58195	2.08090	-2.53656
H	2.69266	2.71557	-0.87773
H	1.30147	1.72685	-1.36255
C	3.28895	-2.84790	-0.57069
H	3.63866	-2.90902	0.46890
H	3.84444	-3.57170	-1.17144
H	2.22839	-3.09027	-0.59369
N	-0.79092	0.70669	-1.15953
C	-1.76589	1.54110	-1.16588
N	-2.04752	2.29880	-2.29711
C	-2.92706	3.39377	-1.95137
C	-3.64753	2.83569	-0.73486
H	-3.60545	3.64050	-2.77074
H	-2.34997	4.29437	-1.69380
N	-2.68199	1.89475	-0.20060
H	-4.57165	2.31201	-1.02037
H	-3.90067	3.60291	-0.00017
C	-1.04646	2.50221	-3.31030
H	-1.52969	2.70213	-4.26925
H	-0.44741	1.59731	-3.39053
H	-0.38331	3.34546	-3.07056
C	-3.12291	1.05032	0.88276
H	-2.41595	0.24391	1.06041
H	-4.09780	0.60331	0.64241
H	-3.22650	1.63716	1.79828
Ni	0.60301	1.13612	1.87980
C	2.31705	0.74463	2.21795
O	3.40336	0.46504	2.44337
C	0.33358	2.84547	1.43005
O	0.16120	3.93066	1.10738
C	-0.45262	0.54078	3.19911
O	-1.13156	0.12046	4.01714

References

[1] J. Saame, T. Rodima, S. Tshepelevitsh, A. Kütt, I. Kaljurand, T. Haljasorg, I. A. Koppel, I. Leito, *J. Org. Chem.* **2016**, *81*, 17, 7349–7361.

[2] R. Uson, A. Laguna, M. Laguna, D. A. Briggs, H. H. Murray, J. P. Fackler. In *Inorganic Syntheses* (Ed.: H. D. Kaesz), John Whiley & Sons, New York, **1989**, pp 85-91.

[3] N. Marion, O. Navarro, J. Mei, E. D. Stevens, N. M. Scott, S. P. Nolan, *J. Am. Chem. Soc.* **2006**, *128*, 4101-4111.

[4] U. Berens, U. Englert, S. Gyser, J. Runsink, A. Salzer, *Eur. J. Org. Chem.* **2006**, 2100-2109.

[5] K. Abdur-Rashid, T. P. Fong, B. Greaves, D. G. Gusev, J. G. Hinman, S. E. Landau, A. J. Lough, R. H. Morris, *J. Am. Chem. Soc.* **2000**, *122*, 9155-9171.

[6] D. G. Gusev, *Organometallics* **2009**, *28*, 6458-6461.

[7] O. V. Dolomanov, L. J. Bourhis, R. J. Gildea, J. A. K. Howard, H. Puschmann, *.J. Appl. Crystallogr.* **2009**, *42*, 339–341.

[8] L. Palatinus, G. Chapuis, *J. Appl. Crystallogr.* **2007**, *40*, 786–790.

[9] M. Saitow, U. Becker, C. Riplinger, E. F. Valeev, F. Neese, *J. Chem. Phys.* **2017**, *146*, 164105.

[10] (a) G. M. Sheldrick, *Acta Cryst.* **2015**, *A71*, 3–8; (b) G. M. Sheldrick, *Acta Cryst.* **2015**, *C71*, 3–8.

[11] A. L. Spek, *Acta Cryst.* **2015**, *C71*, 9–18.

[12] M. J. Frisch, G. W. Trucks, H. B. Schlegel, G. E. Scuseria, M. A. Robb, J. R. Cheeseman, G. Scalmani, V. Barone, B. Mennucci, G. A. Petersson, H. Nakatsuji, M. Caricato, X. Li, H. P. Hratchian, A. F. Izmaylov, J. Bloino, G. Zheng, J. L. Sonnenberg, M. Hada, M. Ehara, K. Toyota, R. Fukuda, J. Hasegawa, M. Ishida, T. Nakajima, Y. Honda, O. Kitao, H. Nakai, T. Vreven, J. A. Montgomery, Jr., J. E. Peralta, F. Ogliaro, M. Bearpark, J. J. Heyd, E. Brothers, K. N. Kudin, V. N. Staroverov, R. Kobayashi, J. Normand, K. Raghavachari, A. Rendell, J. C. Burant, S. S. Iyengar, J. Tomasi, M. Cossi, N. Rega, J. M. Millam, M. Klene, J. E. Knox, J. B. Cross, V. Bakken, C. Adamo, J. Jaramillo, R. Gomperts, R. E. Stratmann, O. Yazyev, A. J. Austin, R. Cammi, C. Pomelli, J. W. Ochterski, R. L. Martin, K. Morokuma, V. G. Zakrzewski, G. A. Voth, P. Salvador, J. J. Dannenberg, S. Dapprich, A. D. Daniels, Ö. Farkas, J. B. Foresman, J. V. Ortiz, J. Cioslowski, D. J. Fox., Gaussian 09. Gaussian, Inc.: Wallingford CT, 2009.

[13] J. Saame, T. Rodima, S. Tshepelevitsh, A. Kütt, I. Kaljurand, T. Haljasorg, I. A. Koppel, I. Leito, *J. Org. Chem.* **2016**, 81, 7349-7361.
